# Valorization of Desugarized Sugar Beet Molasses to Develop a Culture Medium for the Enumeration of Yeasts and Moulds

**DOI:** 10.3390/foods15203760

**Published:** 2026-10-21

**Authors:** Olja Todorić, Verica Aleksić Sabo, Petar Knežević, Jovana Kos, Dragana Tomanić, Žarko Kevrešan, Ljubiša Šarić

**Affiliations:** 1 Institute of Food Technology in Novi Sad, University of Novi Sad, Bulevar cara Lazara 1, 21000 Novi Sad, Serbia; jovana.kos@fins.uns.ac.rs (J.K.); dragana.tomanic@fins.uns.ac.rs (D.T.); zarko.kevresan@fins.uns.ac.rs (Ž.K.); 2 Department of Biology and Ecology, Faculty of Sciences, University of Novi Sad, Trg Dositeja Obradovica 2, 21000 Novi Sad, Serbia; verica.aleksic@dbe.uns.ac.rs (V.A.S.); petar.knezevic@dbe.uns.ac.rs (P.K.)

**Keywords:** desugarized sugar beet molasses, osmophilic yeasts, xerophilic moulds, low water activity, fungal enumeration, method validation

## Abstract

Desugarized sugar beet molasses (DSBM), a nutrient-rich by-product of sugar production, is a promising and largely unexplored substrate for the production of microbiological media. This study aimed to develop and validate a ready-to-use (RTU) medium based on DSBM for the enumeration of yeasts and moulds in foods and feeds with water activity (a_w_) less than or equal to 0.95. DSBM from three production seasons was characterized physicochemically and microbiologically and used to prepare three medium variants, evaluated for productivity and specificity, and evaluated in a single-laboratory method comparison study conducted in accordance with the method comparison protocol described in ISO 16140-2:2016. The three DSBM batches obtained from different production seasons covered dry matter contents of 49.33–52.00%, total sugar contents of 9.46–11.88%, and nitrogen contents of 1.68–1.95%. Potassium and sodium predominated among the minerals. Microbial counts were below the limit of quantification, with no detectable *Salmonella* spp. or *Listeria monocytogenes*. All medium variants met the productivity criterion specified in ISO 21527-2:2008 and completely inhibited the growth of 21 tested bacteria. They showed close agreement with the reference DG18 agar across six food and one feed category (0.60 < a_w_ ≤ 0.95), with mean biases within ±0.025 log_10_ CFU/g and all results within the predefined acceptance limit of ±0.5 log_10_ CFU/g. All 80% β-expectation tolerance intervals in the AP were within the ±0.5 log_10_ CFU/g acceptance limit, and proficiency testing schemes confirmed acceptable performance. Therefore, the novel medium provides a new value-added application for DSBM, supporting the valorization of this underutilized resource from sugar beet processing.

## 1. Introduction

Microbiological control is a critical component of food safety, particularly in the context of extensive production systems, global trade, and increasingly complex supply chains [1]. Although pathogens remain a major public health concern worldwide and have traditionally been the primary focus of food safety efforts [2], spoilage microorganisms, particularly osmophilic yeasts and xerophilic moulds, also represent a significant challenge for the food industry. These fungi are capable of growing in products with reduced water activity (a_w_), including dried fruits, confectionery products, jams, cereals, nuts, spices, and other low-moisture foods, leading to undesirable sensory changes, reduced shelf life, and product rejection [3,4,5]. Furthermore, certain fungal species produce mycotoxins, toxic secondary metabolites associated with various adverse health effects, including carcinogenicity, immunotoxicity, hepatotoxicity, and neurotoxicity in humans and animals [6]. Therefore, reliable detection and enumeration of osmophilic yeasts and xerophilic moulds are important for assessing the microbiological quality and safety of low-a_w_ food products and depend on the availability of suitable selective microbiological media. Several selective media have been developed for the enumeration of fungi in foods with reduced a_w_. Among the most widely used are Dichloran 18% glycerol (DG18) agar, Malt yeast 50% glucose (MY50G) agar, and Potato dextrose agar (PDA) supplemented with high concentrations of sugars to enhance selectivity towards osmotolerant and xerotolerant fungi [3,7,8].

In general, commercial microbiological media are available as dehydrated formulations or ready-to-use (RTU) products. Dehydrated media require several preparation steps, including weighing, dissolution, sterilization, and quality control, whereas RTU media require little or no preparation before use, thereby reducing preparation time and laboratory workload [9,10]. However, the direct cost of RTU media may be higher than that of in-house preparation, which can limit their use in resource-constrained laboratories [11].

These limitations have led to increased interest in developing sustainable microbiological media from locally available industrial by-products and waste streams. Desugarized sugar beet molasses (DSBM) is a residual by-product generated during industrial sugar beet processing after sugar extraction [12,13,14]. Unlike conventional sugar beet molasses, DSBM contains lower concentrations of fermentable sugars while retaining a complex composition of organic and inorganic nutrients, including nitrogenous compounds, minerals, amino acids, raffinose, and betaine [12,14]. The large quantities of DSBM generated during sugar production, along with the need for further value-added applications, have stimulated interest in its valorization [13,14,15]. DSBM has been investigated for various applications, including polyhydroxyalkanoate (PHA) production, use in concentrated form as a source of supplemental energy and protein for cattle consuming low-quality forages, and biogas production [12,13,14,15,16,17].

However, its potential as a substrate for microbiological media remains largely unexplored, despite its nutrient-rich composition. The use of DSBM as a component of microbiological media could reduce the amount of conventional nutrients required and provide an additional application for this sugar processing by-product. However, the composition of industrial by-products may vary between production batches, and this variability could affect the performance of the resulting medium.

The aim of this study was to develop and evaluate a novel RTU medium based on DSBM for the enumeration of yeasts and moulds in food products with a_w_ of 0.95 or lower. To achieve this objective, DSBM obtained from three production seasons was characterized for physicochemical composition and microbiological quality. The resulting DSBM-based RTU medium was then evaluated in a single-laboratory method comparison study conducted in accordance with the method comparison protocol described in ISO 16140-2:2016 [18].

## 2. Materials and Methods

### 2.1. Sampling

DSBM samples from three production seasons (2019, 2020, and 2022) were obtained from Sunoko sugar factory (Pećinci, Serbia). One representative DSBM production batch from each season was included in the study. Prior to analysis, samples were stored in sealed, opaque glass bottles under refrigerated conditions (2–8 °C). DSBM is a moderately viscous, fully liquid material with a colour comparable to that of conventional sugar beet molasses.

### 2.2. Physicochemical Analyses of DSBM

The following physicochemical analyses were performed on DSBM samples obtained from three production seasons (2019, 2020, and 2022). All determinations were performed in triplicate.

#### 2.2.1. Determination of Dry Matter Content

The refractometric dry substance (RDS, %) of DSBM was determined according to ICUMSA GS4/3/8-13 [19].

#### 2.2.2. Determination of Sugar Content

The sugar content of DSBM was determined in accordance with the standard method SRPS E.L3.020:1963 [20] for molasses.

#### 2.2.3. Determination of Sucrose, Fructose, and Glucose Content

The sucrose content of DSBM was determined by high-performance anion-exchange chromatography with pulsed amperometric detection (HPAEC-PAD) according to the official ICUMSA method GS4-22 [21]. Glucose and fructose were determined by HPAEC-PAD using a Dionex ICS-6000 ion chromatography system (Thermo Fisher Scientific, San Jose, CA, USA) equipped with an electrochemical detector and a Thermo Scientific Dionex CarboPac PA20 column (3 × 150 mm) with a suitable guard column (Thermo Fisher Scientific, Sunnyvale, CA, USA). The column temperature was maintained at 30 °C. Samples were introduced in loop mode with a sample loading volume of 5000 µL (5.0 mL) at a delivery speed of 4.0 mL/min. Separation was performed isocratically using 10 mM NaOH (Centrohem, Stara Pazova, Serbia) at a flow rate of 0.500 mL/min for 30 min, followed by washing with 200 mM NaOH for 10 min and re-equilibration with 10 mM NaOH for 20 min. Detection was performed by pulsed amperometric detection (PAD) using a gold working electrode and an Ag/AgCl reference electrode with the carbohydrate quadruple-potential waveform. The chromatographic conditions were based on the approach described by Weitzhandler et al. [22] and Torbica et al. [23]. External calibration was performed using five calibration levels in the range of 2–72 mg/L. Linearity was evaluated by linear regression and will be reported as the coefficient of determination (R^2^) for each analyte. Recovery was not experimentally determined in the original analytical procedure and therefore no recovery value is reported. Sample preparation was performed based on a modification of previously described procedures [24,25]. Briefly, 100 ± 0.5 mg of desugared molasses was mixed with 0.5 mL of methanol (Fisher Scientific, Loughborough, UK) and kept for 5 min in a closed centrifuge tube to inactivate enzymes and minimize excessive starch hydrolysis during extraction. Subsequently, 20 mL of hot water (80 °C) containing 50 mg/L sodium azide (Thermo Fisher Scientific Chemicals, Inc., Ward Hill, MA, USA) was added, and the mixture was vortexed and continuously mixed in a Gyratory Water Bath Shaker (New Brunswick Scientific, Edison, NJ, USA) at 250 rpm for 10 min. The mixture was then centrifuged at 1520× *g* for 5 min at 20 °C, and the supernatant was transferred into a 100 mL volumetric flask. The extraction was repeated with an additional 20 mL of hot water (80 °C) containing 50 mg/L sodium azide, and the supernatants were combined. After cooling to room temperature, 200 µL each of Carrez I (150 mg/mL) and Carrez II (230 mg/mL) solutions were added for protein precipitation. The extracts were made up to 100 mL with ultrapure water and centrifuged at 3000× *g* for 10 min at 20 °C. The resulting supernatants were filtered through a 0.45 µm nylon syringe filter (Fisher Scientific, Pittsburgh, PA, USA) into HPLC vials prior to HPAEC-PAD analysis.

#### 2.2.4. Determination of Metal Content

The concentrations of calcium, copper, iron, magnesium, manganese, potassium, sodium, and zinc in DSBM were determined by atomic absorption spectrometry according to the standard method ISO 6869:2000 [26].

#### 2.2.5. Determination of Nitrogen Content

The nitrogen content of DSBM was determined using the Kjeldahl method according to ISO 1871:2009 [27].

#### 2.2.6. Determination of a_w_

The a_w_ of DSBM samples were determined according to ISO 18787:2017 [28].

#### 2.2.7. Determination of pH

The pH of DSBM was measured potentiometrically according to the modified standard method SRPS E.L3.020:1963 [20], as documented in method FINSLab-5.4-3M-031. The modifications involved sample preparation and electrode calibration. Briefly, DSBM was diluted 1:1 with distilled water and subjected to heating in a boiling water bath for 10 min with gentle mixing. The pH electrode was calibrated at pH 7.0 and 4.0 prior to measurement.

### 2.3. Determination of the Microbiological Quality of DSBM

Microbiological examinations of the DSBM samples were carried out by enumeration of total viable microorganisms [29], yeasts and moulds [7], coagulase positive *staphylococci* [30] (ISO), beta-glucuronidase-positive *Escherichia coli* [31], Enterobacteriaceae [32], coliform bacteria [33], sulfite-reducing *Clostridia* [34] and presumptive *Bacillus cereus* [35]. Detection of *Salmonella* spp. and *Listeria monocytogenes* was performed according to ISO 6579-1:2017 [36] and ISO 11290-1:2017 [37], respectively. All experiments were performed in triplicate.

### 2.4. Development and Physicochemical Characterization of the DSBM-Based RTU Medium

The DSBM-based RTU medium was developed as a selective formulation for the enumeration of yeasts and moulds in low-a_w_ food and feed samples. Its laboratory prototype was formulated from DSBM (<5% *w*/*w*), glucose (<4% *w*/*w*) (Himedia, Mumbai, India), anhydrous glycerol (<5% *w*/*w*) (Himedia, Mumbai, India), agar (<2% *w*/*w*) (Himedia, Mumbai, India), citric acid (<0.2% *w*/*w*) (Ensign Industry, Weifang, China), and deionized water. Chloramphenicol (<0.2% *w*/*w*) (Himedia, Mumbai, India) was incorporated into the formulation prior to sterilization. The glucose content in the medium was optimized using the strains *Zygosaccharomyces rouxii* ATCC 28253 and *Zygosaccharomyces bisporus* ATCC 34890 (Microbiologics, Saint Cloud, MN, USA). Optimization was performed by comparing the growth of these two yeast strains on the newly developed medium and DG18 agar (Himedia, Mumbai, India). Three DSBM-based RTU medium variants were prepared using DSBM from three production seasons (2019, 2020, and 2022). These variants were designated DSBM-I, DSBM-II, and DSBM-III, respectively, and were further evaluated as alternative medium in the validation study. The laboratory prototype was initially developed in 2019 using DSBM obtained in the same production season, and this batch was also used for participation in a proficiency testing (PT) scheme in 2019. The DSBM batches obtained from the 2019, 2020, and 2022 production seasons were stored in sealed, opaque glass bottles under refrigerated conditions (2–8 °C) prior to use. At the time of physicochemical characterization and evaluation in 2025, the respective DSBM batches had therefore been stored for approximately 6, 5, and 3 years, respectively. The target a_w_ of 0.95 was adjusted by varying the proportions of DSBM and deionized water, followed by fine adjustment after incorporation of the remaining medium components. The pH was set to 5.1 ± 0.1 with citric acid prior to autoclaving. Agar was added at the concentration required to obtain a suitable gel consistency for microbiological analysis and handling. The medium components were weighed on analytical (Sartorius, Göttingen, Germany) and technical (Boeco, Hamburg, Germany) balances. The formulation was manually homogenized during heating until all components were completely dissolved in deionized water obtained from a laboratory water purification system (Adrona, Riga, Latvia). The a_w_ and pH were measured with an a_w_ metre (Novasina, Lachen, Switzerland) and a pH metre (Xylem Analytics, Weilheim, Germany), following ISO 18787:2017 [28] and the protocol described in Section 2.2.7, respectively. The pH of the medium was also measured after sterilization. The prepared medium was sterilized by autoclaving at 121 °C for 15 min. Following sterilization, the medium was aseptically dispensed into sterile Petri dishes at 20 mL per plate using a laboratory dispenser (Eppendorf, Hamburg, Germany).

The final quantitative composition of the medium is not disclosed due to ongoing intellectual property protection procedures.

### 2.5. Microbiological Performance Evaluation of the DSBM-Based RTU Medium

Three independent DSBM-based RTU medium variants (DSBM-I, DSBM-II and DSBM-III) were evaluated for productivity and selectivity according to the performance criteria established for DG18 agar in ISO 21527-2:2008 [7]. All selectivity and productivity assessments were performed in triplicate for each medium variant.

#### 2.5.1. Productivity Assessment

Productivity was assessed by calculating the productivity ratio (PR) using the following reference strains: *Aspergillus caesiellus* ATCC 42693 (formerly classified as *A. restrictus* ATCC 42693), *Eurotium rubrum* ATCC 42690, *Wallemia mellicola* ATCC 42694 (formerly classified as *W. sebi* ATCC 42694), and *Saccharomyces cerevisiae* ATCC 9763 (Microbiologics, Saint Cloud, MN, USA). These strains correspond to the reference strains specified in ISO 21527-2:2008 [7] for productivity testing of DG18 agar.

(a) Inoculum preparation: Mould suspensions were prepared from 7-day-old cultures grown on PDA (Himedia, Mumbai, India) plates, which were aseptically overlaid with 10 mL of sterile 0.1% (*w*/*v*) peptone water (Himedia, Mumbai, India). Conidia were released by gently scraping the colony surface with a sterile 10 µL inoculating loop (JiangSu Huida Medical Instruments Co., Ltd., Yancheng, Jiangsu, China), and the suspension was adjusted to a final volume of 10 mL with sterile 0.1% (*w*/*v*) peptone water [38]. Yeast cells were aseptically transferred into sterile 0.1% (*w*/*v*) peptone water and homogenized using a vortex mixer (Velp Scientifica Srl, Usmate Velate, Italy). The yeast suspension was prepared from 3-day-old culture grown on PDA plate and adjusted to a 0.5 McFarland standard using a densitometer (DEN-1, Biosan, Riga, Latvia) [39]. Decimal serial dilutions of the individual yeast and mould suspensions were then prepared in sterile 0.1% (*w*/*v*) peptone water. To verify inoculum concentration, the counts of viable yeast cells and mould spores in the individual working suspensions were determined by surface plating 0.1 mL of appropriate decimal dilutions onto PDA. The inoculated plates were incubated aerobically at 25 ± 1 °C for 3 and 7 days for yeasts and moulds, respectively, after which colonies were enumerated.

(b) Productivity testing: Productivity was assessed quantitatively. Aliquots (0.1 mL) of each working suspension were inoculated onto the surface of the DSBM-based RTU medium (test medium) and Sabouraud dextrose agar (SDA) (Himedia, Mumbai, India) (reference medium) and evenly distributed using a sterile Drigalski spatula (Paul Marienfeld GmbH & Co. KG, Lauda-Königshofen, Germany). A multiple-dilution approach was used, and colony counts were determined using the most appropriate dilution according to the applicable colony-counting criteria. The inoculated plates were incubated aerobically at 25 ± 1 °C for 5 days. After incubation, colonies of test microorganisms were enumerated.

(c) PR calculation: The productivity ratio (PR) was calculated as PR = Ns/No, where Ns and No represent the colony counts obtained on the DSBM-based RTU medium and SDA, respectively, in accordance with ISO 11133:2014 [9].

#### 2.5.2. Selectivity Assessment

A panel of Gram-positive and Gram-negative bacterial strains, including *Enterococcus faecalis* ATCC 19433, *Enterococcus faecalis* ATCC 29212, *Staphylococcus epidermidis* ATCC 12228, *Escherichia coli* ATCC 8739, *Escherichia coli* ATCC 10536, *Rhodococcus equi* ATCC 6939, *Bacillus spizizenii* ATCC 6633, *Pseudomonas aeruginosa* ATCC 27853, *Pseudomonas aeruginosa* ATCC 10145, *Proteus hauseri* ATCC 13315, *Klebsiella aerogenes* ATCC 13048, *Kocuria rhizophila* ATCC 9341, *Listeria innocua* ATCC 33090, *Listeria ivanovii* ATCC 19119, *Salmonella enterica* subsp. *enterica* ser. *Enteritidis* ATCC 13076, *Salmonella enterica* subsp. *enterica* ser. *Typhimurium* ATCC 14028, *Listeria monocytogenes* ATCC 19111, *Streptococcus mutans* ATCC 25175, *Staphylococcus aureus* ATCC 25923, *Bacillus cereus* ATCC 11778, and *Yersinia enterocolitica* ATCC 27729 (Microbiologics, Saint Cloud, MN, USA), was used to assess selectivity. The bacterial strains are also summarized in Supplementary Table S2, which provides information on their respective roles in the selectivity evaluation. Additional bacterial strains not specified in ISO 21527-2:2008 [7] were included to broaden the bacterial panel and provide a more comprehensive assessment of the selectivity of the developed medium toward bacteria relevant to food and feed. Selectivity testing was performed qualitatively [9]. The bacterial suspensions were prepared from active working cultures of the respective reference strains, and the inoculum density was standardized to 0.5 McFarland, as measured with a DEN-1 densitometer (Biosan, Riga, Latvia). The surface of the test medium was inoculated with each test bacterium as a single straight line using a 1 µL inoculation loop (JiangSu Huida Medical Instruments Co., Ltd., Yancheng, Jiangsu, China). Since 1 µL of each suspension was used for inoculation, the approximate inoculum applied to each plate was 1.5 × 10^5^ CFU. The inoculated plates were incubated aerobically at 25 ± 1 °C for 5 days, after which the presence or absence of visible bacterial growth was assessed [7].

### 2.6. Evaluation of the DSBM-Based RTU Medium

The DSBM-based RTU medium was evaluated in a single-laboratory method comparison study conducted in accordance with the protocol described in ISO 16140-2:2016 [18], using a study design comprising relative trueness and accuracy profile (AP) assessments. The alternative method consisted of the replacement of DG18 agar with the DSBM-based RTU medium, while all other analytical steps remained unchanged and were performed according to ISO 21527-2:2008 [7]. To assess the effect of raw material variability on medium performance, three DSBM-based RTU medium variants (DSBM-I, DSBM-II, and DSBM-III) were evaluated. Each alternative medium variation was compared with DG18 agar as the reference medium under identical analytical conditions described in ISO 21527-2:2008 [7].

#### 2.6.1. Relative Trueness Study

The relative trueness study was conducted as a comparative assessment between the reference and alternative media using both naturally and artificially contaminated samples, in accordance with ISO 16140-2:2016 [18]. The study was designed to evaluate the applicability of the DSBM-based RTU medium across a wide range of food and feed matrices with different a_w_ levels. It included six food categories and one feed category defined according to a_w_. Although ISO 16140-2:2016 [18] gives preference to naturally contaminated samples for relative trueness studies when available, the low a_w_ values of the selected food and feed matrices limited the availability of naturally contaminated samples. Therefore, artificially contaminated samples were additionally included to ensure sufficient sample numbers and adequate representation of the tested product categories. The tested product types and the number of samples included in each category are summarized in Table 1. Each category comprised three product types, with five samples analyzed per product type, resulting in a total of 15 samples per category.

A total of 105 samples were analyzed, comprising 90 food and 15 feed samples, of which 35 were naturally contaminated and 70 artificially contaminated (Table 1).

##### Artificial Contamination of Foods Using Spiking Protocol

The reference strains used in this study were *Aspergillus caesiellus* ATCC 42693, *Eurotium rubrum* ATCC 42690, *Wallemia mellicola* ATCC 42694 and *Saccharomyces cerevisiae* ATCC 9763. Reference strains were reconstituted according to the manufacturer’s instructions and maintained as stock cultures on PDA slants. Working cultures were prepared under aseptic conditions by subculturing the reference strains onto PDA in sterile Petri dishes. Yeasts were incubated for 3 days and moulds for 7 days at 25 ± 1 °C under aerobic conditions.

In accordance with the spiking protocol described in ISO 16140-2:2016 [18], mixed cultures were exposed to sublethal stress conditions prior to inoculation into food and feed matrices. The stress conditions were selected to simulate those encountered by yeasts and moulds in the tested products.

(a) Preparation of working suspensions of test microorganisms: according to the protocol described in Section 2.5.1.

(b) Preparation of mixed inoculum and adjustment of concentration: As ISO 16140-2:2016 [18] describes injury protocols primarily for pure cultures, the protocol was adapted in the present study by applying stress treatments to mixed cultures containing the target yeast and mould strains. The individual suspensions of *S. cerevisiae*, *A. caesiellus*, *E. rubrum*, and *W. mellicola* were combined to obtain approximately equal contributions of each strain to the final mixed inoculum based on viable counts. The resulting mixed inoculum was then adjusted to the target contamination level for each experimental trial prior to injury induction, with the concentration verified by enumeration of viable cells. This approach ensured that the artificially contaminated samples covered the range of contamination levels representative of the tested food and feed matrices, in accordance with ISO 16140-2:2016 [18].

(c) Application of injury protocols: The prepared mixed inoculum, adjusted to the target contamination level for each experimental trial, was subjected to selected stress conditions in sterile test tubes to induce sublethal injury while maintaining microbial culturability. Detailed stress parameters are provided in Supplementary Table S1.

(d) Injury measurement: The efficiency of injury induction was evaluated by comparing viable counts of the mixed inoculum before and after application of the stress treatments. Enumeration was performed in parallel on DG18 agar and non-selective PDA. A reduction exceeding 0.5 log_10_ CFU/mL compared with the corresponding pre-stress counts was considered indicative of sufficient stress application. The inoculated plates were incubated aerobically at 25 ± 1 °C for 7 days prior to enumeration.

(e) Preparation of inoculum for food and feed spiking: The mixed inoculum obtained after stress exposure was diluted, if necessary, with sterile 0.1% (*w*/*v*) peptone water to achieve the target contamination level. The inoculum was aseptically added directly into a sterile Stomacher bag containing the test portion. The inoculum volume was minimized to avoid significant changes in sample a_w_ [18]. Sterile 0.1% (*w*/*v*) peptone water was then added as required, and the sample was homogenized using a Stomacher homogenizer (AES Chemunex, Bruz, France) to ensure uniform distribution of the inoculum.

(f) Enumeration of yeasts and moulds in artificially contaminated samples: Artificially contaminated food and feed samples were enumerated by spread-plating appropriate dilutions onto the alternative DSBM-based RTU medium and DG18 agar. Plates were incubated aerobically at 25 ± 1 °C and examined after 3 and 7 days for yeasts and moulds, respectively, according to ISO 21527-2:2008 [7]. For validation enumeration, the 7-day incubation period for moulds was selected to ensure adequate development of slow-growing moulds, particularly *Wallemia mellicola*, which produces small colonies on both DG18 agar and the DSBM-based RTU medium.

##### Calculation and Interpretation of Relative Trueness

Relative trueness was evaluated in accordance with ISO 16140-2:2016 [18] (Clause 6.1.2.3) by comparing the quantitative results obtained using the DSBM-based RTU medium (DSBM-I, DSBM-II, and DSBM-III) with those obtained using the reference DG18 agar. Results obtained with both media were expressed as log_10_ CFU/g prior to statistical analysis. For each tested sample, the difference between the result obtained with the alternative medium and that obtained with the reference medium was calculated. Method agreement was evaluated using Bland–Altman analysis, including calculation of the mean difference (bias) and the corresponding 95% limits of agreement. Bland–Altman difference plots were used to assess the distribution of individual differences and identify potential systematic or proportional bias across the investigated concentration range. Scatter plots including the identity line (X = Y) were generated to visualize the agreement between the alternative and reference methods. The performance of each DSBM-based RTU medium was interpreted in accordance with the acceptance criteria defined in ISO 16140-2:2016 [18]. Particular attention was given to the presence of systematic bias and to the consistency of agreement between the alternative and reference media across the investigated concentration range, food and feed categories, and a_w_ levels.

#### 2.6.2. AP Study

The AP study was performed to further characterize the performance of the developed DSBM-based RTU medium variations by evaluating the trueness and precision across predefined contamination levels and representative low-a_w_ food and feed matrices.

Artificially contaminated samples prepared as described in Section Artificial Contamination of Foods Using Spiking Protocol were used for the study. One representative product type was selected from each of the seven aw categories, as listed in Table 1. For each category, six samples of the selected product type were analyzed, with two samples prepared at each contamination level (low, intermediate, and high), covering the expected contamination range of the selected product type. For each sample, five replicate determinations were performed using five independent test portions from the same sample. The results were used to calculate the central value in accordance with ISO 16140-2:2016 [18].

According to ISO 16140-2:2016 [18], APs were constructed using 80% β-expectation tolerance intervals (β-ETIs), integrating bias and variability to provide an overall assessment of quantitative method performance. An acceptability limit (AL) of ±0.5 log_10_ CFU/g was applied as the maximum acceptable deviation for quantitative microbiological methods. The DSBM-based RTU medium variations were considered equivalent to the reference DG18 agar when the upper and lower β-ETI limits remained within the predefined ALs throughout the tested contamination range. Calculations and construction of the APs were performed using the ISO 16140-2 AP spreadsheet.

### 2.7. External Quality Assessment

The DSBM-based RTU medium was evaluated in non-pathogen PT schemes organized by HPA/FEPTU (NP063) in 2019 and UK Health Security Agency/FEPTU (NP080) in 2025. In the 2019 PT scheme, DSBM-I was evaluated, whereas the 2025 PT scheme included all three DSBM-based RTU medium variations (DSBM-I, DSBM-II and DSBM-III). PT samples were analyzed using the DSBM-based RTU medium according to the analytical procedure described in ISO 21527-2:2008 [7]. The obtained results were evaluated according to the criteria established by the PT providers. The PT samples consisted of freeze-dried food containing target yeasts and moulds together with accompanying bacterial populations at assigned concentrations.

### 2.8. Statistical Analysis

Physicochemical data were expressed as the mean ± standard deviation (SD). Microbiological results were expressed as log_10_ CFU/g. Differences in the productivity of the medium variants prepared from three different DSBM batches were evaluated by one-way analysis of variance (ANOVA), followed by Duncan’s multiple range test. Statistical significance was accepted at *p* < 0.05. Statistical analyses were performed using STATISTICA version 10 (StatSoft Inc., Tulsa, OK, USA). Statistical calculations related to novel medium evaluation, including relative trueness and AP study, were performed according to ISO 16140-2:2016 [18] and are described in the Section Calculation and Interpretation of Relative Trueness and Section 2.6.2, respectively.

## 3. Results and Discussion

### 3.1. Physicochemical Characterization of DSBM and Its Relevance for Medium Development

The physicochemical characteristics of three DSBM batches obtained from different production seasons are presented in Table 2.

Dry matter content ranged from 49.33% to 52.00%, while total sugar content varied between 9.46% and 11.88% among the three DSBM batches obtained from different production seasons. Although DSBM is obtained through substantial removal of sucrose from conventional sugar beet molasses during sugar production [14], residual sugars remained a notable component of the by-product (Table 2). Schmid et al. [14] reported sucrose contents of 17.6% and 13.2% and dry matter contents of 70.7% and 71.6% in DSBM obtained from the 2016 and 2017 production seasons, respectively. Variations in the chemical composition of DSBM may arise from differences in the origin of the sugar beets, the quality and consistence of the molasses, and the efficiency of the desugarization process [14]. Variation in dry matter may affect the concentration of nutrients and other soluble components introduced into the formulation, whereas the contribution of sugars is of direct relevance to the intended application of the developed medium. The investigated DSBM batches showed relatively narrow ranges of dry matter and total sugar content, which should be considered in the context of their contribution to the final medium formulation, where DSBM accounted for less than 5.0% (*w*/*w*). (The proportion of DSBM in the final medium is not disclosed due to ongoing intellectual property protection procedures.)

Sugars serve as available sources of carbon and energy for fungal growth [40,41], while their concentration also influences the osmotic environment of the cultivation medium [42]. This is particularly relevant for osmophilic yeasts and xerophilic moulds, which are adapted to growth under conditions of reduced a_w_ associated with high concentrations of soluble sugars [4]. Fungal growth is also influenced by species- and strain-specific differences in the ability to utilize individual carbohydrates [43,44]. Sucrose was clearly the predominant individual sugar in DSBM, whereas glucose and fructose were present at substantially lower concentrations (Table 2). For example, *Schizosaccharomyces osmophilus* ferments glucose, whereas sucrose utilization has been reported to be absent or delayed [45]. Furthermore, certain moulds, such as *Rhizopus nigricans*, have been reported to be unable to utilize sucrose [46]. Glucose was therefore included in the formulation to provide an additional readily available carbon source for fungi with limited ability to utilize sucrose. The total sugar content was determined using a standard method for molasses and therefore should not be interpreted as the arithmetic sum of the individual sugars quantified by HPLC.

Nitrogen (N) content ranged from 1.68% to 1.95% among the three DSBM batches obtained from different production seasons (Table 2), with values comparable to those reported by Schmid et al. [14] for DSBM from the 2016 and 2017 production seasons (1.8% and 2.1%, respectively). N is an essential nutrient for fungal growth, being required for the synthesis of amino acids, proteins, and nucleotides, while its availability also affects fungal development and secondary metabolism [47,48]. Considering that DSBM accounted for less than 5.0% (*w*/*v*) of the final formulation (the proportion of DSBM in the final medium is not disclosed due to ongoing intellectual property protection procedures), its contribution to the total nitrogen content of the developed medium was estimated at less than 0.84–0.97 g N/L. Mineral analysis showed that potassium (K) and sodium (Na) were the predominant elements, followed by calcium (Ca), magnesium (Mg), and iron (Fe), while manganese (Mn), zinc (Zn), and copper (Cu) were present only at trace levels, with Cu being detected only in trace amounts or not detected at all depending on the production season (Table 2). K is an essential cation for fungi, and contributes to cellular homeostasis and growth [46,49,50,51,52]. In contrast, Na is generally required in more limited amounts, and excessive intracellular Na^+^ can disrupt cellular homeostasis [51]. Essential mineral elements such as Mg, Ca, Fe, Zn, and Mn are required by fungi for growth [40,46]. Although K and Na were present in high contents in the investigated by-product (Table 2), the concentrations contributed by DSBM to the final formulation were considerably lower. Calculated concentrations in the developed medium indicated K levels below 1.74–2.28 g/kg, Na levels of 1.82–2.05 g/kg, Mg levels of 5.85–13.05 mg/kg, Ca levels of 9.33–17.35 mg/kg, Fe levels of 1.84–3.52 mg/kg, and Zn and Mn levels in the sub-mg/kg range. Trace elements such as Fe, Zn, and Mn are required in low concentrations, primarily as cofactors of enzymes and other functional proteins [46]. The measured a_w_ and pH values indicated consistency among the investigated DSBM batches (Table 2).

### 3.2. The Microbiological Quality of DSBM

Counts of total viable microorganisms, yeasts and moulds, coagulase-positive staphylococci, β-glucuronidase-positive *Escherichia coli*, Enterobacteriaceae, coliform bacteria, sulfite-reducing *Clostridia*, and presumptive *Bacillus cereus* in tested DSBM samples were below the limits of quantification (<1 CFU/mL), while *Salmonella* spp. and *Listeria monocytogenes* were not detected. These results are consistent with the physicochemical characteristics of DSBM presented in Table 2. Although the pH values were close to neutrality (6.96–7.13), which alone would not restrict microbial growth, DSBM exhibited high dry matter content (49.33–52.00%) and low a_w_ (0.77–0.78). The a_w_ is one of the main determinants of microbial survival and growth, and values around or below 0.80 generally restrict the growth of most bacteria, whereas selected xerophilic moulds and osmophilic yeasts can grow at lower a_w_ values [4]. In addition, the relatively high concentrations of dissolved minerals, particularly K and Na (Table 2), may contribute to the osmotic stress associated with the matrix. Therefore, the combined effects of low a_w_, high solids content, and osmotic stress could explain the absence of the tested microorganisms in all analyzed samples. In addition, chromatographic fractionation and concentration by water evaporation during DSBM production may further reduce its microbial load [14,15]. The results indicate that the DSBM batches used in this study were microbiologically stable.

### 3.3. Characteristics of the Developed DSBM-Based RTU Medium

**Figure 1:**
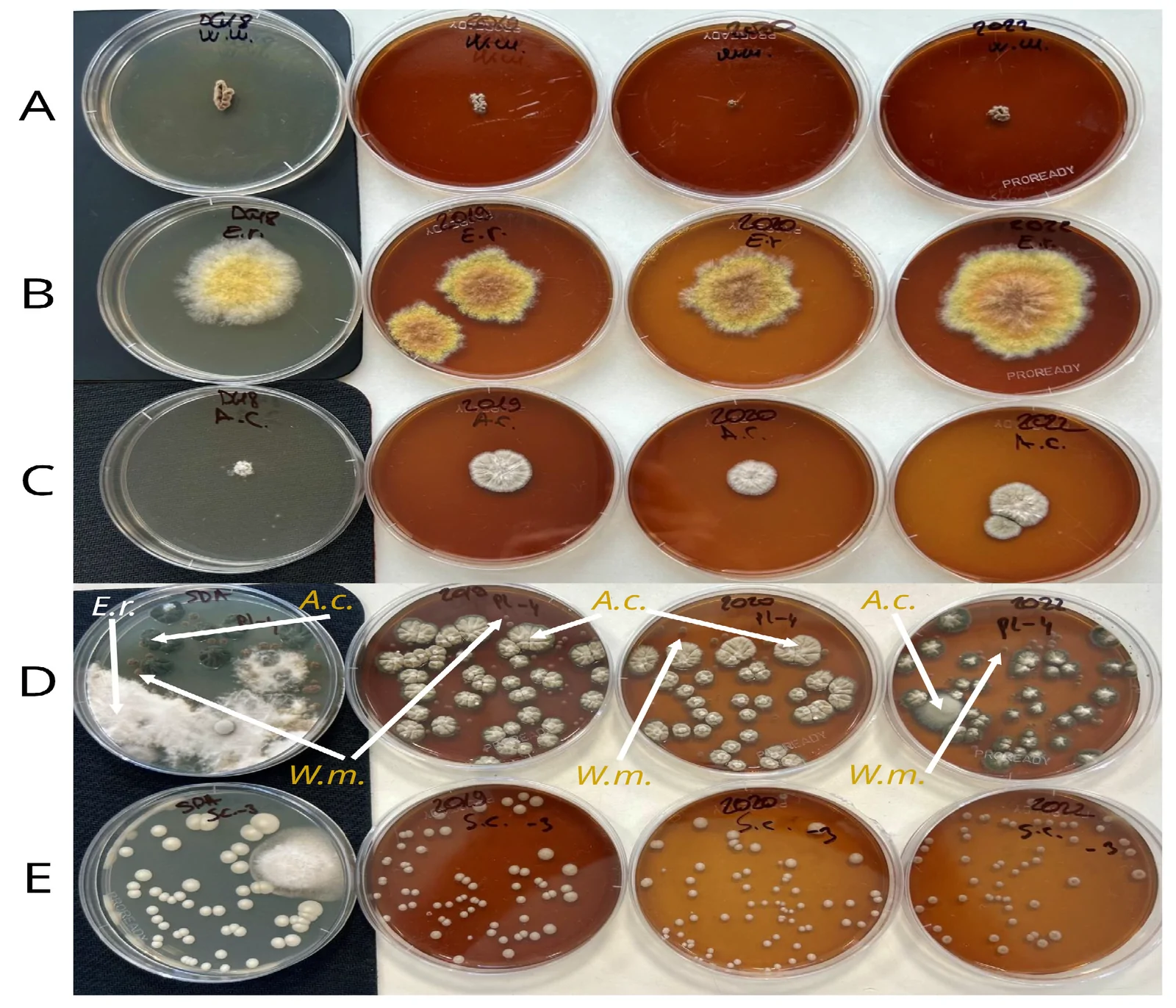
Growth of selected fungal strains on DSBM-based medium and conventional DG18 and SDA media. Note: Panels A–E present representative growth of reference cultures: (**A**) *Wallemia mellicola*; (**B**) *Eurotium rubrum*; (**C**) *Aspergillus caesiellus*; (**D**) mixed culture comprising reference mould strains (A.c., *Aspergillus caesiellus*; W.m., *Wallemia mellicola*); and (**E**) *Saccharomyces cerevisiae*. SDA, Sabouraud dextrose agar; DG18, Dichloran 18% glycerol.

The developed DSBM-based RTU medium exhibited a uniform appearance and homogeneous gel structure suitable for microbiological analysis (Figure 1). Although some differences in colony morphology were observed between the DSBM-based and reference media, these differences did not interfere with the recognition and routine enumeration of colonies. The characteristic dark brown colour of the DSBM-based RTU medium is primarily attributed to melanins, melanoidins, and alkaline degradation products of hexoses (ADHs), which are the major colourants formed during sugar beet processing [53]. The formulated medium was homogeneous, with no visible phase separation, precipitation, or syneresis. It achieved the target a_w_ of 0.95 after adjustment of the DSBM-to-water ratio, followed by fine-tuning after incorporation of the remaining components.

In addition to naturally occurring minerals and organic compounds derived from DSBM (Table 2), glucose was added as an additional readily available carbon source. Although sucrose represents the predominant carbohydrate in DSBM (Table 2), its utilization depends on invertase activity and may vary among yeast strains [54,55]. Glycerol was included as an a_w_-limiting solute to facilitate the recovery of *Wallemia* species under reduced-a_w_ conditions [56,57]. The soluble mineral and organic components naturally present in DSBM, together with glycerol and the other formulation components, may contribute to the overall osmotic effect and were therefore considered during formulation development. Experimental optimization showed that DSBM itself was the predominant factor influencing the a_w_ of the formulation, with DSBM in water resulting in an a_w_ close to the target value even before the addition of the remaining formulation components. Thus, the final a_w_ of the developed medium was primarily determined by the soluble components originating from DSBM, while glycerol and glucose made comparatively smaller contributions to a_w_ reduction. The resulting a_w_ was consistent with the requirements for fungal enumeration media intended for the recovery of xerophilic moulds and osmophilic yeasts from low a_w_ matrices [7]. The reduced a_w_ also contributes to the selectivity of the medium against bacterial growth [58]. After autoclaving, the pH of the formulated medium remained within the target range of 5.1 ± 0.1, slightly lower than that of DG18 agar (pH 5.6 ± 0.2). This acidic pH is consistent with the conditions generally used for the cultivation of yeasts and moulds and may favour their growth while limiting the growth of many bacterial populations [59].

Unlike DG18 agar, the DSBM-based formulation contains nutrients originating from a food-processing by-product, including residual carbohydrates, minerals, and other organic compounds. Since DSBM composition varies between production batches (Table 2), medium prepared with DSBM from three production seasons was included in the performance evaluation.

### 3.4. Microbiological Performance of the DSBM-Based RTU Medium

The productivity ratios (PRs) obtained for the developed DSBM-based RTU medium, calculated relative to SDA, met the minimum acceptance criterion specified in ISO 21527-2:2008 [7] for all tested reference strains, with several PR values exceeding 1.0 (Table 3).

PR values above 1.0 were obtained for some strains, particularly *S. cerevisiae* and *A. caesiellus* (Table 3). Although this may reflect favourable growth conditions on the DSBM-based medium, variability associated with inoculum preparation, fungal propagule distribution, and colony enumeration should also be considered when interpreting these results [7]. No statistically significant differences were observed among the three DSBM-based RTU medium variations prepared using DSBM from the three production seasons (*p* > 0.05) (Table 3). All three variations fulfilled the ISO 21527-2:2008 [7] acceptance criterion for each tested strain, and comparable productivity was observed for the three medium variants under the conditions investigated.

Selectivity testing demonstrated complete inhibition of all 21 tested bacterial strains. The additional strains extended the selectivity assessment beyond those specified in ISO 21527-2:2008 [7]. The observed selectivity may be attributed to the combined effects of reduced a_w_, acidic pH, and chloramphenicol supplementation, which suppressed bacterial growth while preserving fungal recovery [7,60]. This balance is important for reliable detection and enumeration of yeasts and moulds, as bacterial overgrowth can interfere with the growth and appearance of fungal colonies [61,62]. Alongside the production of antifungal compounds, antagonistic bacteria can compete with fungi for nutrients and space within the growth medium [63]. As reported in the literature, bacterial overgrowth may interfere with the recognition of fungal colonies, whereas the absence of bacterial growth facilitates their recognition based on characteristic morphological features, including colony margins, pigmentation, and sporulation [7,64,65]. All three DSBM-based medium variants met the productivity and selectivity requirements applied in this study and were included in the evaluation.

### 3.5. Method Comparison Study Results

The results of the relative trueness and AP studies are presented below to assess the equivalence of the alternative method across a broad range of food and feed matrices and contamination levels.

#### 3.5.1. Relative Trueness Study Results

The relative trueness study was performed to evaluate the agreement between the results obtained on the alternative medium variations (DSBM-I, DSBM-II and DSBM-III) and those obtained on the reference medium (DG18 agar), using the same procedure described in ISO 21527-2:2008 [7], with the only difference being the medium used. The comparison included products covering a broad range of a_w_ values (0.60 < a_w_ ≤ 0.95) and matrix characteristics, including dairy products (hard cheeses), fermented and cured meat products, fruit-based products (marmalades and fruit fillings), confectionery products (candies and dried fruits), cereals, flours, legumes, and feed.

**Figure 2:**
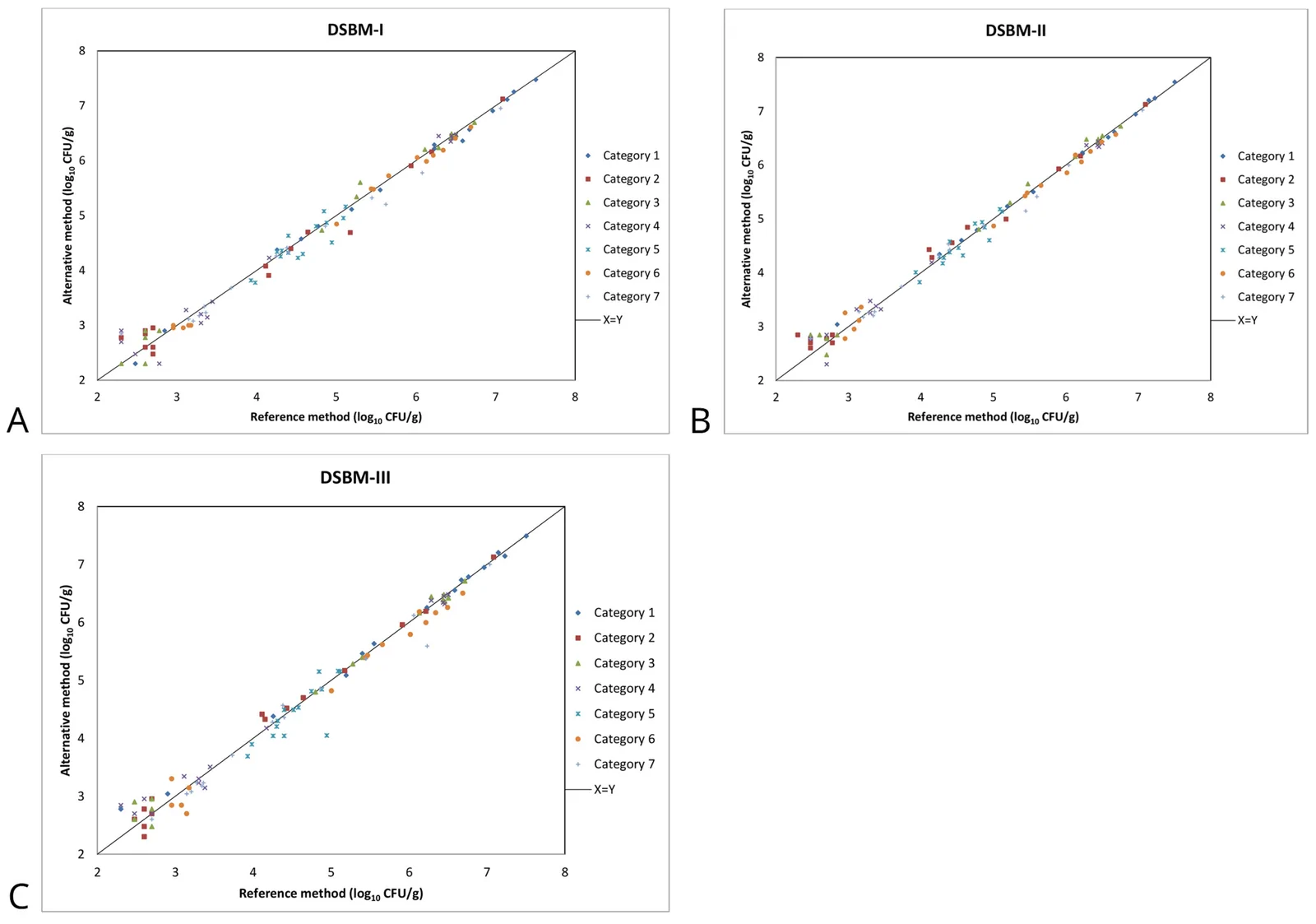
A scatter plot of results obtained with the reference DG18 medium and the alternative DSBM medium across all tested categories. (**A**) DSBM-I medium variation, (**B**) DSBM-II medium variation, (**C**) DSBM-III medium variation. Note: The solid line represents the identity line (X = Y). Results are expressed as log_10_ CFU/g. Each point represents one product category comprising three product types, with five samples analyzed per product type (15 samples per category). The figure was generated based on the tabulated results and calculations performed in accordance with ISO 16140-2:2016, Clause 6.1.2.3. The categories included in the study are listed in Table 1.

**Figure 3:**
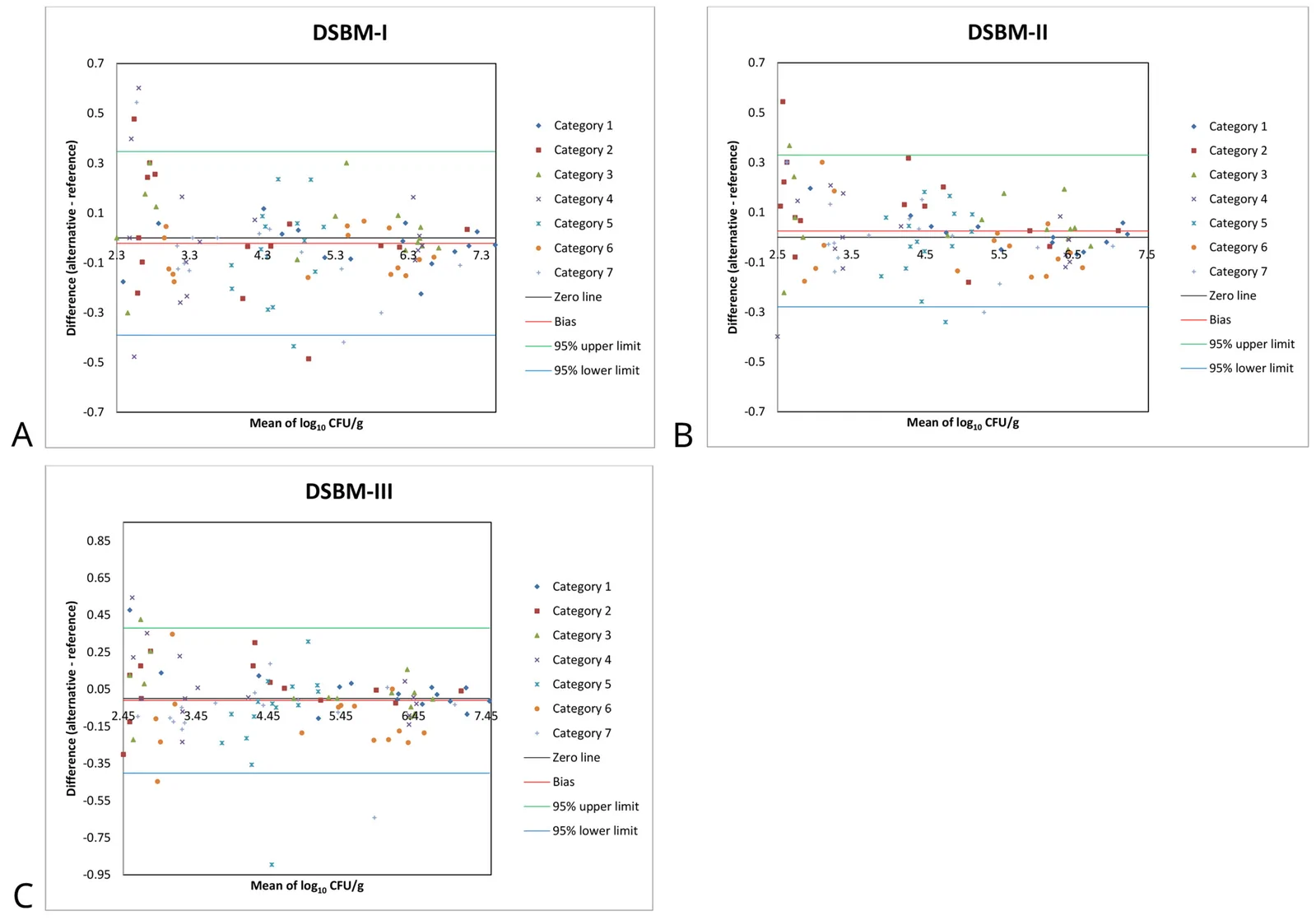
A Bland–Altman plot comparing the alternative DSBM medium and the reference DG18 medium across all tested categories. (**A**) DSBM-I medium variation, (**B**) DSBM-II medium variation, (**C**) DSBM-III medium variation. Note: The horizontal red line represents the mean bias, while the green and blue lines indicate the 95% limits of agreement. Each point represents one product category comprising three product types, with five samples analyzed per product type (15 samples per category). The figure was generated based on the tabulated results and calculations performed in accordance with ISO 16140-2:2016, Clause 6.1.2.3. The categories included in the study are presented in Table 1.

No clear matrix-dependent differences in agreement between the DSBM-based medium variations and DG18 agar were observed (Figure 2 and Figure 3). For all three DSBM-based medium variations, scatter plots (Figure 2) showed that the results were closely distributed around the identity line (X = Y) throughout the investigated concentration range, indicating that the use of alternative medium did not exhibit systematic bias compared with DG18 agar. Slightly greater dispersion was observed at lower contamination levels, which is consistent with the greater relative uncertainty of plate counts at low microbial concentrations. However, this pattern was consistent across all variations and was not associated with any particular food or feed category.

Bland–Altman analysis (Figure 3) confirmed these observations. For the DSBM-I, the mean bias was –0.022 log_10_ CFU/g, with 95% limits of agreement ranging from –0.391 to 0.347 log_10_ CFU/g (Figure 3). The DSBM-II showed a mean bias of 0.025 log_10_ CFU/g and slightly narrower limits of agreement (–0.279 to 0.330 log_10_ CFU/g), whereas the DSBM-III produced a mean bias of –0.011 log_10_ CFU/g with limits of agreement between –0.402 and 0.380 log_10_ CFU/g. In all cases, the observed biases were close to zero, all limits of agreement remained within the generally accepted ±0.5 log_10_ CFU/g criterion for quantitative microbiological method evaluation [18], and no proportional bias was evident across the investigated concentration range. Agreement between the two media was comparable across the seven product categories, with no consistent positive or negative differences associated with a particular a_w_ range or matrix type.

Minor isolated deviations exceeding approximately ±0.4 log_10_ CFU/g were observed only in a small number of individual samples (Figure 3), predominantly at low microbial counts, and are consistent with the inherent variability of quantitative microbiological enumeration rather than systematic differences between media.

The relative trueness results showed close agreement between all three DSBM-based medium variants and DG18 agar across the tested food and feed matrices.

#### 3.5.2. Accuracy Profile Study Results

All three DSBM-based RTU medium variations fulfilled the predefined AP acceptance criteria across all investigated product categories (Table 4).

**Figure 4:**
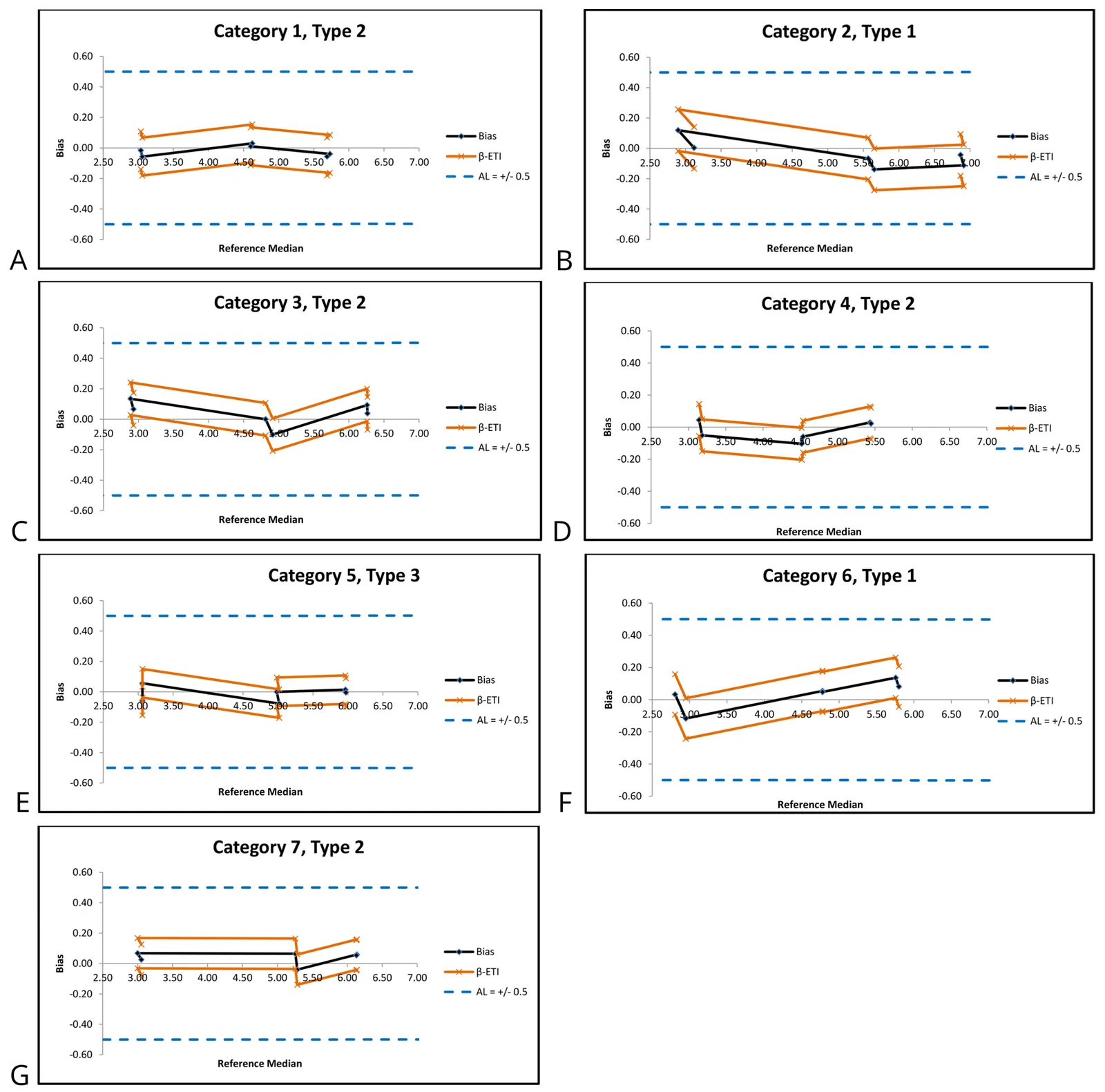
The accuracy profile comparing DSBM-I and reference DG18 media across the seven tested categories, each represented by one selected product type. (**A**) Category 1, Type 2, (**B**) Category 2, Type 1, (**C**) Category 3, Type 2, (**D**) Category 4, Type 2, (**E**) Category 5, Type 3, (**F**) Category 6, Type 1, (**G**) Category 7, Type 2. Note: Bias is expressed as log_10_ CFU/g. Black lines indicate bias, orange lines indicate the 80% β-expectation tolerance intervals (β-ETI), and blue dashed lines indicate the acceptability limits (AL) of ±0.5 log_10_ CFU/g. Two samples were analyzed at each contamination level (low, intermediate, and high), with five replicate determinations performed per sample. The categories and selected product types included in the study are listed in Table 1.

**Figure 5:**
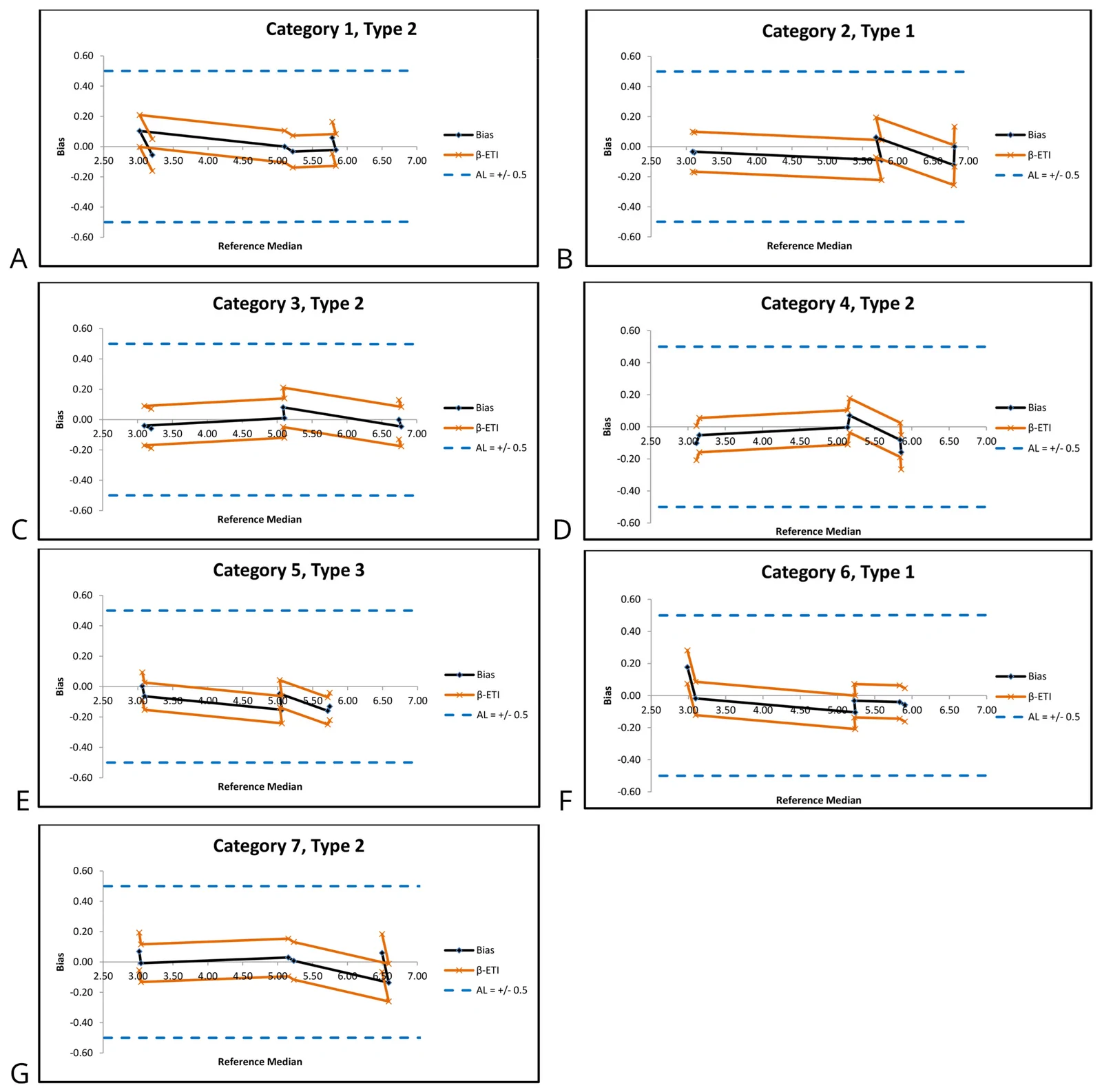
The accuracy profile comparing DSBM-II and reference DG18 media across the seven tested categories, each represented by one selected product type. (**A**) Category 1, Type 2, (**B**) Category 2, Type 1, (**C**) Category 3, Type 2, (**D**) Category 4, Type 2, (**E**) Category 5, Type 3, (**F**) Category 6, Type 1, (**G**) Category 7, Type 2. Note: Bias is expressed as log_10_ CFU/g. Black lines indicate bias, orange lines indicate the 80% β-expectation tolerance intervals (β-ETI), and blue dashed lines indicate the acceptability limits (AL) of ±0.5 log_10_ CFU/g. Two samples were analyzed at each contamination level (low, intermediate, and high), with five replicate determinations performed per sample. The categories and selected product types included in the study are listed in Table 1.

**Figure 6:**
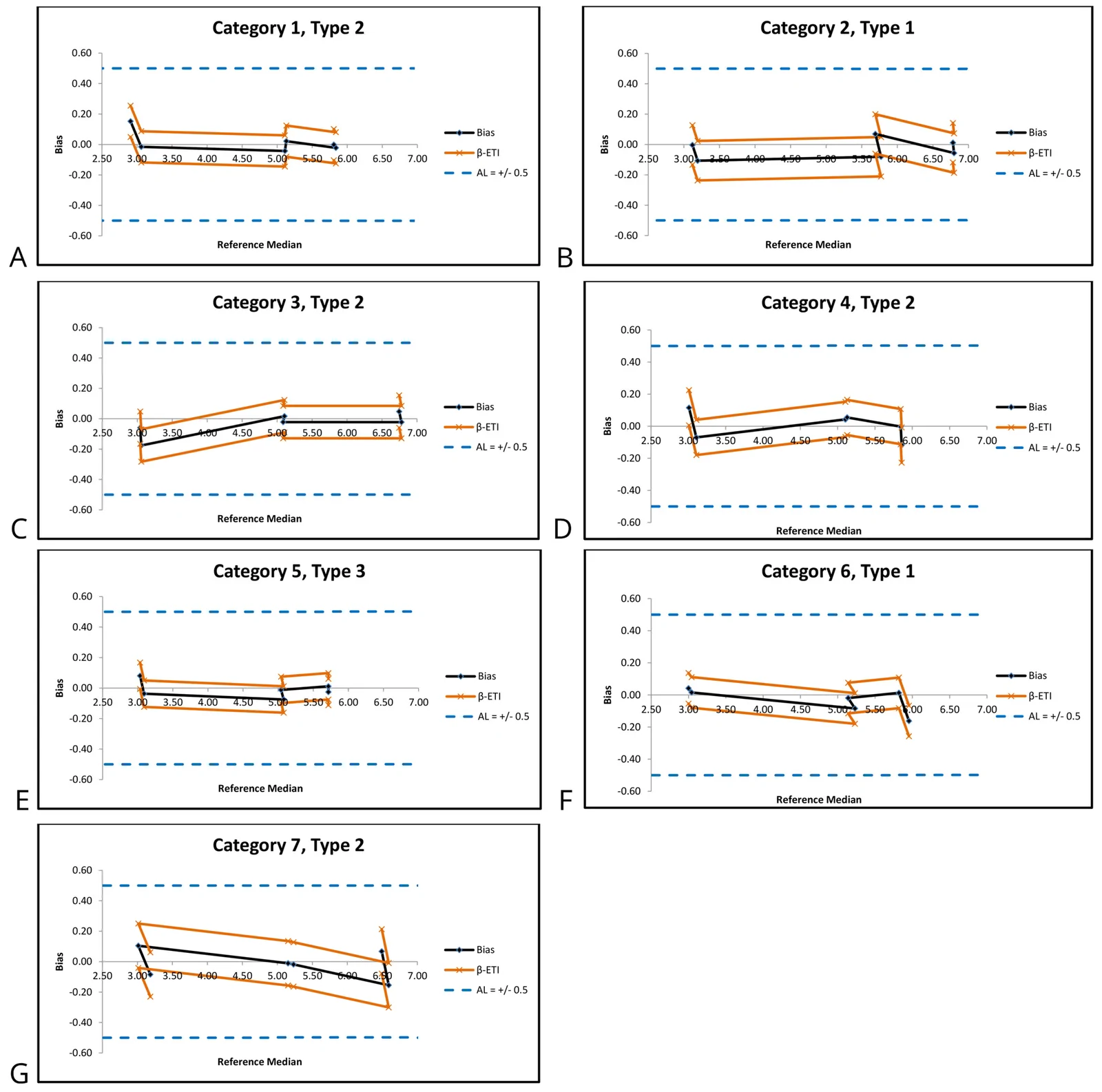
The accuracy profile comparing DSBM-III and reference DG18 media across the seven tested categories, each represented by one selected product type. (**A**) Category 1, Type 2, (**B**) Category 2, Type 1, (**C**) Category 3, Type 2, (**D**) Category 4, Type 2, (**E**) Category 5, Type 3, (**F**) Category 6, Type 1, (**G**) Category 7, Type 2. Note: Bias is expressed as log_10_ CFU/g. Black lines indicate bias, orange lines indicate the 80% β-expectation tolerance intervals (β-ETI), and blue dashed lines indicate the acceptability limits (AL) of ±0.5 log_10_ CFU/g. Two samples were analyzed at each contamination level (low, intermediate, and high), with five replicate determinations performed per sample. The categories and selected product types included in the study are listed in Table 1.

For all tested matrices and medium variants, the lower and upper limits of the 80% β-ETI remained within the ±0.5 log_10_ CFU/g acceptability limits. The developed medium maintained acceptable performance across matrices with different compositional characteristics and a_w_ levels, including challenging low-a_w_ products such as flours, candies, dried fruits, and feeds. This is particularly relevant for low-a_w_ foods, where reduced water availability may affect fungal viability and contribute to heterogeneous microbial distribution. Representative AP plots of DSBM I-III medium variations are presented in Figure 4, Figure 5 and Figure 6.

For the evaluated matrices, AP showed low bias across the investigated concentration range, with no consistent concentration-dependent tendency toward positive or negative bias in the DSBM-based RTU medium (Figure 4, Figure 5 and Figure 6). Although some variation in bias direction was observed between contamination levels, all values remained within the predefined acceptance limits. Across all tested categories, bias ranged from −0.139 to 0.136 log_10_ CFU/g for DSBM-I, from −0.160 to 0.178 log_10_ CFU/g for DSBM-II, and from −0.176 to 0.152 log_10_ CFU/g for DSBM-III (Table 4). Comparable AP parameters were obtained for all three medium variants, indicating that the differences in DSBM composition among production seasons had no measurable effect on medium performance.

The precision of the developed media, expressed as the repeatability standard deviation (SD), was consistent across all investigated categories, with values ranging from 0.060 to 0.101 log_10_ CFU/g. Although minor differences were observed among matrices, the variability remained within acceptable limits, probably reflecting the expected contribution of individual analytical steps, including inoculum preparation, plating, and colony enumeration.

The relative trueness and AP results demonstrate that DSBM-based RTU medium provides performance equivalent to the reference DG18 agar for enumeration of yeasts and moulds in low-a_w_ food and feed products, fulfilling the performance requirements of ISO 16140-2:2016 [18]. The similar performance of the three variants indicates that DSBM from different production seasons can be used without adversely affecting the analytical performance of the medium.

### 3.6. External Quality Assessment Results

The performance of the DSBM-based RTU media was also evaluated through independent external proficiency testing for yeast and mould enumeration (Table 5 and Table 6). All reported results obtained with the evaluated medium variations achieved satisfactory performance according to the PT assessment criteria, demonstrating agreement with the assigned values.

All calculated z-scores were within the acceptance criteria of the respective PT schemes (Table 5 and Table 6). No fungal growth was observed in samples without assigned target yeast or mould levels, with the exception of sample NP0215 analyzed using DSBM-II in the UKHSA/FEPTU NP080 proficiency testing scheme (Table 6). The assigned value for this sample was 0 log_10_ CFU/g, whereas 0.3 log_10_ CFU/g was obtained with DSBM-II. This result corresponded to only two mould colonies. Given the very low number of colonies observed and the absence of an assigned target level, this finding was most likely attributable to incidental contamination during the analytical procedure rather than to a lack of an inadequate performance of the medium. Further support for this interpretation is provided by the satisfactory performance of DSBM-II for the other PT sample (NP0216), for which a z-score of 0.73 was reported (Table 6). Three analysts participated in the proficiency testing scheme, with each analyst working with a different DSBM-based medium variant.

## 4. Limitations

A limitation of the present study is that the storage stability and shelf life of the prepared RTU plates were not evaluated within the scope of this manuscript. Therefore, although the medium was prepared and used in the present study as an RTU formulation, the present work does not provide experimental evidence for its defined shelf life or storage period.

Another limitation is that the interlaboratory study required to assess the reproducibility of the developed medium across independent laboratories has not yet been conducted. The present study therefore provides evidence from a single-laboratory method comparison study, while interlaboratory reproducibility remains to be established.

## 5. Conclusions

This study demonstrated that DSBM can be used as a raw material for formulating a selective RTU medium for the enumeration of yeasts and moulds in low-a_w_ food and feed products. Despite seasonal variation in DSBM composition, all three medium variants showed satisfactory productivity and complete inhibition of the bacterial strains included in the selectivity assessment. Evaluation against reference DG18 agar in a single-laboratory method comparison study conducted in accordance with the method comparison protocol of ISO 16140-2:2016 demonstrated close agreement between the two media across a broad range of food and feed matrices, with low biases and all β-expectation tolerance intervals within the predefined acceptability limits. The results from independent proficiency testing provided additional evidence of the performance of the developed medium. Across the investigated food and feed matrices, no excessive spreading or overgrowth of mould colonies that interfered with reliable enumeration was observed, despite the absence of dedicated spreading-growth inhibitors in the formulation. These findings indicate that additional spreading-growth inhibitors were not necessary for the developed formulation under the conditions investigated. Therefore, the DSBM-based RTU medium represents a promising alternative to DG18 agar for routine enumeration of yeasts and moulds in low-a_w_ food and feed products, while providing a new value-added application for the sugar beet processing by-product. An interlaboratory study has not yet been conducted and will be performed to assess the reproducibility of the developed medium across independent laboratories and to provide additional evidence to support its wider application as an alternative culture medium within the method described in ISO 21527-2:2008. Further studies should focus on assessing the storage stability and shelf life of the prepared RTU plates under defined refrigerated storage conditions to further characterize their suitability for routine laboratory use.

## Figures and Tables

**Table 1:** Food and feed matrices included in the relative trueness study.

Category	a_w_	Product Types	Number of Samples		
			Naturally Contaminated	Artificially Contaminated	Total
Food Category 1	0.91 < a_w_ ≤ 0.95	hard cheeses	5	0	5
		fruit fillings	0	5	5
		dry-cured meat products	5	0	5
Food Category 2	0.87 < a_w_ ≤ 0.91	dry-fermented sausages	5	0	5
		cooked sausages	5	0	5
		dry-cured meat products	0	5	5
Food Category 3	0.80 < a_w_ ≤ 0.87	dry-fermented sausages	5	0	5
		dry-cured meat products	5	0	5
		fruit jams	0	5	5
Food Category 4	0.75 < a_w_ ≤ 0.80	dry-cured meat products	5	0	5
		fruit jams	0	5	5
		confectionery products	0	5	5
Food Category 5	0.65 < a_w_ ≤ 0.75	beans	0	5	5
		dried and candied fruits	0	5	5
		flours	0	5	5
Food Category 6	0.60 < a_w_ ≤ 0.65	candies	0	5	5
		dried and candied fruits	0	5	5
		flours	0	5	5
Feed Category 7	0.60 < a_w_ ≤ 0.95	pet food sausages	0	5	5
		starter feeds for young animals	0	5	5
		grain-based feeds	0	5	5

Note: Each product type represents samples obtained from different manufacturers. The number of samples indicates the total number of analyzed samples within each product type (n = 5). a_w_, water activity.

**Table 2:** Physicochemical characteristics of three DSBM batches originating from different production seasons.

Parameter	Unit	2019	2020	2022
Dry Matter (RDS)	%	52.00 (0.02)	50.23 (0.01)	49.33 (0.01)
Total Sugar Content	%	9.46 (0.05)	11.88 (0.05)	9.89 (0.10)
Sucrose	g/100 g	9.27 (0.04)	11.62 (0.06)	9.71 (0.05)
Glucose	g/100 g	0.04 (0.004)	0.06 (0.003)	0.05 (0.003)
Fructose	g/100 g	0.06 (0.003)	0.05 (0.004)	0.03 (0.004)
Nitrogen	%	1.74 (0.01)	1.68 (0.01)	1.95 (0.02)
Calcium	mg/kg	346.80 (0.76)	186.50 (3.97)	206.00 (8.04)
Copper	mg/kg	n.d.	0.90 (0.02)	n.d.
Iron	mg/kg	70.40 (0.25)	36.80 (0.88)	37.10 (0.76)
Magnesium	mg/kg	176.90 (1.25)	261.00 (0.51)	117.00 (0.62)
Manganese	mg/kg	3.50 (0.03)	3.15 (0.05)	6.20 (0.32)
Potassium	mg/kg	34,882.91 (284.3)	38,555.95 (204.63)	45,600.00 (319.2)
Sodium	mg/kg	39,709.29 (235.1)	41,081.19 (41,63)	36,470.00 (20.82)
Zinc	mg/kg	7.33 (0.38)	7.70 (0.14)	8.10 (0.25)
a_w_	-	0.77 (0.02)	0.78 (0.02)	0.77 (0.02)
pH	-	7.10 (0.08)	6.96 (0.08)	7.13 (0.08)

Results were expressed as mean ± standard deviations of triplicate analyses for all measurements. n.d.: not detected.

**Table 3:** Productivity ratios (PRs) of developed DSBM-based RTU medium.

Reference Strain	DSBM-I	DSBM-II	DSBM-III	Acceptance Criterion
*A. caesiellus*	1.24 ± 0.36 ^a^	1.23 ± 0.10 ^a^	1.06 ± 0.22 ^a^	≥0.5
*E. rubrum*	0.82 ± 0.27 ^a^	1.15 ± 0.39 ^a^	0.81 ± 0.10 ^a^	
*W. mellicola*	0.81 ± 0.04 ^a^	0.83 ± 0.08 ^a^	0.88 ± 0.06 ^a^	
*S. cerevisiae*	1.06 ± 0.07 ^a^	1.11 ± 0.09 ^a^	0.94 ± 0.09 ^a^	

Values are expressed as the mean ± standard deviation (n = 3). Means sharing the same superscript letter are not significantly different (one-way ANOVA, *p* > 0.05). DSBM-I–III, DSBM-based RTU media prepared using DSBM originating from three production seasons.

**Table 4:** Summary of AP parameters for DSBM-based RTU media.

Medium	Category/Product Type	Bias Range (Δ log_10_ CFU/g)	80% β-ETI Range (Δ log_10_ CFU/g)	Repeatability SD (log_10_ CFU/g)
DSBM-I	1/fruit fillings	–0.057 to 0.030	−0.181 to 0.154	0.086
	2/dry-fermented sausages	−0.139 to 0.119	−0.276 to 0.257	0.095
	3/dry-cured meat products	−0.101 to 0.135	−0.208 to 0.242	0.074
	4/fruit jams	−0.104 to 0.045	−0.203 to 0.144	0.069
	5/flours	−0.078 to 0.057	−0.171 to 0.150	0.065
	6/candies	−0.117 to 0.136	−0.242 to 0.262	0.087
	7/starter feeds for young animals	−0.040 to 0.068	−0.140 to 0.168	0.069
DSBM-II	1/fruit fillings	−0.056 to 0.104	−0.161 to 0.209	0.073
	2/dry-fermented sausages	−0.090 to 0.123	−0.255 to 0.194	0.092
	3/dry-cured meat products	−0.059 to 0.081	−0.189 to 0.211	0.090
	4/fruit jams	−0.158 to 0.071	−0.265 to 0.178	0.074
	5/flours	−0.160 to 0.04	−0.249 to 0.093	0.062
	6/candies	−0.105 to 0.178	−0.208 to 0.282	0.072
	7/starter feeds for young animals	−0.136 to 0.069	−0.260 to 0.193	0.086
DSBM-III	1/fruit fillings	−0.042 to 0.152	−0.145 to 0.255	0.071
	2/dry-fermented sausages	−0.107 to 0.069	−0.237 to 0.199	0.090
	3/dry-cured meat products	−0.176 to 0.047	−0.283 to 0.154	0.074
	4/fruit jams	−0.117 to 0.115	−0.227 to 0.225	0.076
	5/flours	−0.075 to 0.081	−0.161 to 0.167	0.060
	6/candies	−0.162 to 0.041	−0.257 to 0.054	0.066
	7/starter feeds for young animals	−0.155 to 0.104	−0.301 to 0.250	0.101

DSBM-I–III: DSBM-based RTU media prepared using DSBM originating from three production seasons; category numbers 1–7 correspond to the product categories defined in Table 1 and are category identifiers, not sample numbers; β-ETI: β-expectation tolerance interval; AL: acceptability limit; SD: repeatability standard deviation of the alternative method.

**Table 5:** Performance of DSBM-based RTU media in external proficiency testing for yeast enumeration.

PT Scheme	Year	Number of Participants	Medium Evaluated	Sample	Assigned Value	Obtained Value	z-Score	Performance Evaluation
HPA/FEPTU NP063	2019	83	DSBM-I	NP0181	Not Assigned	<1	-	Satisfactory
						<1	-	Satisfactory
						<1	-	Satisfactory
				NP0182	4.54	4.23	−0.88	Satisfactory
						4.34	−0.56	Satisfactory
						4.28	−0.75	Satisfactory
UKHSA/FEPTU NP080	2025	68	DSBM-I	NP0215	3.52	3.48	−0.12	Satisfactory
			DSBM-II			3.56	0.10	Satisfactory
			DSBM-III			3.54	0.07	Satisfactory
		67	DSBM-I	NP0216	3.46	3.51	0.13	Satisfactory
			DSBM-II			3.53	0.20	Satisfactory
			DSBM-III			3.56	0.28	Satisfactory

All counts are expressed as log_10_ CFU/mL reconstituted sample. PT: proficiency testing; z-score values were calculated by the PT providers according to the scheme-specific evaluation criteria. Satisfactory performance corresponds to the acceptance criteria defined by the PT providers. <1 indicates results below the quantification level of the method. Therefore, z-score calculation was not applicable.

**Table 6:** Performance of DSBM-based RTU media in external proficiency testing for mould enumeration.

PT Scheme	Year	Number of Participants	Medium Evaluated	Sample	Assigned Value	Obtained Value	z-Score	Performance Evaluation
HPA/FEPTU NP063	2019	83	DSBM-I	NP0181	3.53	3.52	−0.03	Satisfactory
						3.41	−0.33	Satisfactory
						3.20	−0.93	Satisfactory
				NP0182	4.49	4.38	−0.31	Satisfactory
						4.41	−0.21	Satisfactory
						4.20	−0.82	Satisfactory
UKHSA/FEPTU NP080	2025	68	DSBM-I	NP0215	0	0	-	Satisfactory
			DSBM-II			0.3	-	Unsatisfactory
			DSBM-III			0	-	Satisfactory
		67	DSBM-I	NP0216	3.00	3.23	0.66	Satisfactory
			DSBM-II			3.26	0.73	Satisfactory
			DSBM-III			3.15	0.42	Satisfactory

All counts are expressed as log_10_ CFU/mL reconstituted sample. PT: proficiency testing; z-score values were calculated by the PT providers according to the scheme-specific evaluation criteria. Satisfactory performance corresponds to the acceptance criteria defined by the PT providers. <1 indicates results below the quantification level of the method. Therefore, z-score calculation was not applicable.

## Data Availability

The original contributions presented in this study are included in the article and Supplementary Material. Further inquiries can be directed to the corresponding authors.

## Supplementary Materials

The following supporting information can be downloaded at https://www.mdpi.com/article/10.3390/foods15203760/s1, Table S1: Matrix-specific stress conditions applied during injury induction. Table S2: Reference strains used for evaluation of selectivity. Table S3. Raw data used for the accuracy profile assessment obtained using DSBM-I and DG18 media for the enumeration of yeasts and moulds across selected food and feed categories and types. Table S4. Raw data used for the accuracy profile assessment obtained using DSBM-II and DG18 media for the enumeration of yeasts and moulds across selected food and feed categories and types. Table S5. Raw data used for the accuracy profile assessment obtained using DSBM-III and DG18 media for the enumeration of yeasts and moulds across selected food and feed categories and types. Table S6. Raw data and statistical analysis of the agreement between the reference DG18 medium and the alternative DSBM-I medium variation for the enumeration of yeasts and moulds across selected food and feed categories and types. Table S7. Raw data and statistical analysis of the agreement between the reference DG18 medium and the alternative DSBM-II medium variation for the enumeration of yeasts and moulds across selected food and feed categories and types. Table S8. Raw data and statistical analysis of the agreement between the reference DG18 medium and the alternative DSBM-III medium variation for the enumeration of yeasts and moulds across selected food and feed categories and types. Figure S1. Bias and β-ETI limits, compliance with predefined acceptability criteria, and graphical presentation of the accuracy profile (AP) study results for the DSBM-I medium variation. Figure S2. Bias and β-ETI limits, compliance with predefined acceptability criteria, and graphical presentation of the accuracy profile (AP) study results for the DSBM-II medium variation. Figure S3. Bias and β-ETI limits, compliance with predefined acceptability criteria, and graphical presentation of the accuracy profile (AP) study results for the DSBM-III medium variation. Figure S4. Bias and β-ETI limits, compliance with predefined acceptability criteria, and graphical presentation of the accuracy profile (AP) study results for moulds enumeration using the DSBM-I medium variation. Figure S5. Bias and β-ETI limits, compliance with predefined acceptability criteria, and graphical presentation of the accuracy profile (AP) study results for moulds enumeration using the DSBM-II medium variation. Figure S6. Bias and β-ETI limits, compliance with predefined acceptability criteria, and graphical presentation of the accuracy profile (AP) study results for moulds enumeration using the DSBM-III medium variation. Figure S7. Bias and β-ETI limits, compliance with predefined acceptability criteria, and graphical presentation of the accuracy profile (AP) study results for yeast enumeration using the DSBM-I medium variation. Figure S8. Bias and β-ETI limits, compliance with predefined acceptability criteria, and graphical presentation of the accuracy profile (AP) study results for yeast enumeration using the DSBM-II medium variation. Figure S9. Bias and β-ETI limits, compliance with predefined acceptability criteria, and graphical presentation of the accuracy profile (AP) study results for yeast enumeration using the DSBM-III medium variation.

## Abbreviations

The following abbreviations are used in this manuscript:

DG18	Dichloran 18% glycerol agar
MY50G	Malt yeast 50% glucose agar
PDA	Potato dextrose agar
RTU	Ready-to-use products
DSBM	Desugarized sugar beet molasses
PHA	Polyhydroxyalkanoate
RDS	Refractometric dry substance
HPLC	High-performance liquid chromatography
PR	Productivity ratio
SD	Standard deviation
a_w_	Water activity
AP	Accuracy profile
PT	Proficiency testing
LOQ	Limit of quantification
CFU	Colony-forming units
SDA	Sabouraud dextrose agar
ISO	International Organization for Standardization
ATCC	American Type Culture Collection
ANOVA	Analysis of variance
AL	Acceptability limit
β-ETI	β-expectation tolerance interval
