# Supplementary material for: Valorization of Desugarized Sugar Beet Molasses to Develop a Culture Medium for the Enumeration of Yeasts and Moulds
Source: Foods. 2026 Oct 21;15(20):3760. doi: 10.3390/foods15203760 (PMC13704253; doi:10.3390/foods15203760)
Supplement: Supplementary file 1 [file foods-15-03760-s001.zip › foods-4576101-supplementary Table S1.pdf]

Supplementary Table S1. Matrix-specific stress conditions applied during injury induction

Food matrix group	Stress type	Applied conditions	Purpose
[1,2]Hard cheese	Thermal stress	50 °C, 15 min water bath (Raypa, Italy)	Simulation of mild heat treatment and temperature fluctuations during cheese production and ripening
	Osmotic stress	10% NaCl (w/w), 25±1 °C, 24 h	Simulation of salting during cheese manufacture
	Acid stress	pH 5 (lactic acid), 25±1 °C, 24 h	Simulation of fermentation-associated acidity
	Reduced oxygen availability	25±1 °C, 48 h, anaerobic incubation,	Simulation of reduced oxygen availability in cheese matrix
	Cold stress	4±1 °C, 7 days	Simulation of refrigerated storage conditions
	Reduced water activity stress	15% sucrose, 25±1 °C, 24 h	Simulation of reduced water availability
[1,3]Cured meat matrices, including fermented sausages, dry-cured loin, and bacon	Osmotic stress	2.4-3% NaCl, 25±1 °C, 24 h	Simulation of salting process
	Acid stress	pH 5 (lactic acid), +25±1 °C, 24 h	Simulation of fermentation acidity
	Reduced water activity stress	15% sucrose, 25±1 °C, 24 h	Simulation of drying and moisture loss
	Oxygen limitation	Anaerobic incubation, 25±1 °C, 24-48 h	Simulation of vacuum/MAP conditions
[1,4,5,6]Marmalade, fruit fillings, confectionery products	Osmotic stress	35% sucrose, 25±1 °C, 30-60 min	Simulation of high sugar content
	Acid stress	pH 3.0-3.5 (citric acid), 25±1 °C, 30-60 min	Simulation of acidic fruit environment
	Mild heat stress	50 °C, 15 min	Simulation of sublethal heat during cooking and hot filling
[1,7,8,9,10]Beans, flours, dried fruit, feed mixtures	Reduced water activity stress	15% sucrose, 25±1 °C, 24 h	Simulation of low-moisture storage conditions
[1,6,11]Candies, candied fruit confectionery products	Osmotic stress	35% sucrose, 25±1 °C, 30-60 min	Simulation of high sugar concentration.
	Acid stress (only for fruit-based products)	pH 5 (citric acid), 25±1 °C, 24 h	Simulation of acidic fruit environment
	Reduced water activity stress	40% sucrose, 25±1 °C, 24 h	Simulation of moisture limitation
[12]Dog/cat sausage	Reduced water activity stress	40% sucrose, 25±1 °C, 24 h	Simulation of semi-moist product conditions
	Osmotic stress	6-10% NaCl, 25±1 °C, 24 h	Simulation of salt preservation
	Mild heat stress	50 °C, 15 min	Simulation of processing heat

[1]International Organization for Standardization. ISO 16140-2:2016. Microbiology of the Food Chain—Method Validation—Part 2: Protocol for the Validation of Alternative (Proprietary) Methods against a Reference Method; International Organization for Standardization: Geneva, Switzerland, 2016.

[2]Hayaloglu, A.A. Cheese: Microbiology of Cheese. In Reference Module in Food Science; Elsevier: Amsterdam, The Netherlands, 2016. https://doi.org/10.1016/B978-0-08-100596-5.00675-2.

[3]Vuković, I.K. Fundamentals of Meat Technology, 4th ed.; Veterinary Chamber of Serbia: Belgrade, Serbia, 2012; ISBN 978-86-82301-87-5.

[4]O'Beirne, D. Jams and Preserves: Chemistry of Manufacture. In Encyclopedia of Food Sciences and Nutrition, 2nd ed.; Elsevier: Amsterdam, The Netherlands, 2003.

[5]Alam, M. Hydrocolloid-Based Fruit Fillings: A Comprehensive Review on Formulation, Techno-Functional Properties, Synergistic Mechanisms, and Applications. J. Texture Stud. 2024, 55, e12861. https://doi.org/10.1111/jtxs.12861.

[6]Hartel, R.W.; von Elbe, J.H.; Hofberger, R. Confectionery Science and Technology; Springer: Cham, Switzerland, 2018. https://doi.org/10.1007/978-3-319-61742-8.

[7]Sharma, H.K.; Kumar, N., Eds. Agro-Processing and Food Engineering: Operational and Application Aspects; Springer: Singapore, 2022. https://doi.org/10.1007/978-981-16-7289-7.

[8]Catterall, P.; Cauvain, S.P. Flour Milling. In Technology of Breadmaking, 2nd ed.; Cauvain, S.P., Young, L.S., Eds.; Springer: Boston, MA, USA, 2007; pp. 333–369. https://doi.org/10.1007/0-387-38565-7.

[9]Radojčin, M.; Pavkov, I.; Bursać Kovačević, D.; Putnik, P.; Wiktor, A.; Górska, A.; Stamenković, Z. Effect of Selected Drying Methods and Emerging Drying Intensification Technologies on the Quality of Dried Fruit: A Review. Processes 2021, 9, 132. https://doi.org/10.3390/pr9010132.

[10]Rojas, O.J. (Bio)Technological Processing of Poultry and Pig Feed: Impact on the Composition, Digestibility, Anti-Nutritional Factors and Hygiene. Anim. Feed Sci. Technol. 2021, 273, 114850. https://doi.org/10.1016/j.anifeedsci.2020.114850.

[11]Edwards, W.P. The Science of Sugar Confectionery, 2nd ed.; Royal Society of Chemistry: Cambridge, UK, 2018. https://doi.org/10.1039/9781788011334.

[12]Tanprasertsuk, J.; Tate, D.E.; Tarr, D.S.; Shmalberg, J. Does the Definition of Human Ultra-Processed Foods Apply to Dog and Cat Foods? A Review of Pet Food Processing Techniques, Their Impact on Health, and a Call for Pet Food Processing Classification. Front. Vet. Sci. 2026, 13, 1690420. https://doi.org/10.3389/fvets.2026.1690420
