# Supplementary material for: Valorization of Desugarized Sugar Beet Molasses to Develop a Culture Medium for the Enumeration of Yeasts and Moulds
Source: Foods. 2026 Oct 21;15(20):3760. doi: 10.3390/foods15203760 (PMC13704253; doi:10.3390/foods15203760)
Supplement: Supplementary file 1 [file foods-15-03760-s001.zip › foods-4576101-supplementary.pdf]

Table S2. Reference strains used for evaluation of selectivity

Organism	Strain	Role in evaluation
Enterococcus faecalis	ATCC 19433, ATCC 29212	Indicator of fecal contamination and opportunistic pathogen [1]
Staphylococcus epidermidis	ATCC 12228	Commensal and pathogen [2]
Escherichia coli	ATCC 8739, ATCC 10536	Indicator of fecal contamination and pathogen [3]
Rhodococcus equi	ATCC 6939	Environmental contaminant and pathogen [4]
Bacillus spizizenii	ATCC 6633	Spore-forming indicator of environmental contamination [5]
Pseudomonas aeruginosa	ATCC 27853, ATCC 10145	Spoilage/indicator [6]
Proteus hauseri	ATCC 13315	Spoilage organism [7]
Klebsiella aerogenes	ATCC 13048	Enteric indicator [8]
Kocuria rhizophila	ATCC 9341	Environmental Gram-positive [9]
Listeria innocua	ATCC 33090	Listeria surrogate [10]
Listeria ivanovii	ATCC 19119	Listeria surrogate [11]
Salmonella enterica subsp. enterica ser. Enteritidis	ATCC 13076	Pathogen [12]
Salmonella enterica subsp. enterica ser. Typhimurium	ATCC 14028	Pathogen [13]
Listeria monocytogenes	ATCC 19111	Pathogen [14]
Streptococcus mutans	ATCC 25175	Gram-positive indicator [15]
Staphylococcus aureus	ATCC 25923	Pathogen [16]
Bacillus cereus	ATCC 11778	Spore-former [17]
Yersinia enterocolitica	ATCC 27729	Foodborne pathogen [18]

1. Boehm, A.B.; Sassoubre, L.M. Enterococci as indicators of environmental fecal contamination. **2014**.
2. Otto, M. Staphylococcus epidermidis—the'accidental'pathogen. *Nat Rev Microbiol*. **2009**, *7*, 555-567.
3. Nowicki, S.; Delaurent, Z.R.; de Villiers, E.P.; Githinji, G.; Charles, K.J. The utility of Escherichia coli as a contamination indicator for rural drinking water: Evidence from whole genome sequencing. *PLOS ONE* **2021**, *16*, e0245910.

4. Takai, S. Epidemiology of Rhodococcus equi infections: a review. *Vet. Microbiol.* **1997**, *56*, 167-176.
5. Ringleben, L.; Weise, T.; Truong, H.T.T.; Anh, L.H.; Pfaff, M. Experimental and model-based characterisation of Bacillus spizizenii growth under different temperature, pH and salinity conditions prior to aquacultural wastewater treatment application. *Biochem. Eng. J.* **2022**, *187*, 108630.
6. Li, X.; Gu, N.; Huang, T.Y.; Zhong, F.; Peng, G. Pseudomonas aeruginosa: A typical biofilm forming pathogen and an emerging but underestimated pathogen in food processing. *Front. Microbiol.* **2023**, *13*, 1114199.
7. Dewi, F.R.; Stanley, R.; Powell, S.M.; Burke, C.M. Application of electrolysed oxidising water as a sanitiser to extend the shelf-life of seafood products: a review. *J Food Sci Technol.* **2017**, *54*, 1321-1332.
8. Djisalov, M.; Pavlović, M.; Janjušević, L.; Šašić Zorić, L.; Popović, Ž.D.; Gadjanski, I. A Rapid and Sensitive LAMP Assay for the Detection of Klebsiella aerogenes in Food Matrices. *Foods* **2026**, *15*, 1277.
9. Tang, J.S.; Gillevet, P.M. Reclassification of ATCC 9341 from Micrococcus luteus to Kocuria rhizophila. *Int J Syst Evol Microbiol.* **2003**, *53*, 995-997.
10. Friedly, E.; Crandall, P.; Ricke, S.; O'bryan, C.; Martin, E.; Boyd, L. Identification of Listeria innocua surrogates for Listeria monocytogenes in hamburger patties. J *Food Sci.* **2008**, *73*, M174-M178.
11. Espí-Malillos, A.; López-Almela, I.; Ruiz-García, P.; López-Mendoza, M.C.; Carrón, N.; González-Torres, P.; Meza-Torres, J.; Pizarro-Cerdá, J.; Quereda, J.J. Impact of virulence factors overexpression on Listeria monocytogenes F2365 epidemic strain fitness and the limitations of surrogate species in UHT and raw milk. *NPJ Sci Food* **2025**, *10*, 15.
12. Bermudez-Aguirre, D.; Carter, J.; Niemira, B.A. An investigation about the historic global foodborne outbreaks of Salmonella spp. in eggs: From hatcheries to tables. *Compr Rev Food Sci Food Saf.* **2025**, *24*, e70202.
13. Cochrane, R.A.; Huss, A.R.; Aldrich, G.C.; Stark, C.R.; Jones, C.K. Evaluating chemical mitigation of Salmonella Typhimurium ATCC 14028 in animal feed ingredients. *J Food Prot.* **2016**, *79*, 672-676.
14. Osek, J.; Lachtara, B.; Wieczorek, K. Listeria monocytogenes in foods—From culture identification to whole-genome characteristics. *Food Sci. Nutr.* **2022**, *10*, 2825-2854.
15. Piwat, S.; Teanpaisan, R. 16S rRNA PCR-denaturing gradient gel electrophoresis of oral Lactobacillus casei group and their phenotypic appearances. *ISRN Microbiol.* **2013**, *2013*, 342082.
16. Nunes Nascimento, J.C.; Alvarenga, V.O.; Paulino, B.N.; de Cerqueira e Silva,

A.B.; Matos, J.R.; Rosário, I.L.d.S.; Andrade, I.H.P.; Silva, J.G.; Costa, M. Staphylococcus aureus in food safety: antimicrobial resistance, detection technologies, and future perspectives. *Crit. Rev. Food Sci. Nutr.* **2026**, *66*, 607-630.
17. Bottone, E.J. Bacillus cereus, a volatile human pathogen. *Clin Microbiol Rev.* **2010**, *23*, 382-398.
18. Zadernowska, A.; Chajęcka-Wierzchowska, W.; Łaniewska-Trokenheim, Ł. Yersinia enterocolitica: a dangerous, but often ignored, foodborne pathogen. *Food Res. Int.* **2014**, *30*, 53-70.

A

(Food) Category	1
(Food) Type	2

			Reference method					Alternative method				
Sample Name	(Food) item	Level	rep 1	rep 2	rep 3	rep 4	rep 5	rep 1	rep 2	rep 3	rep 4	rep 5
	fruit fillings	low	780	1090	1050	1490	1420	900	1290	1050	1420	990
	fruit fillings	low	1000	1140	1310	1080	1330	1000	880	870	1100	1080
	fruit fillings	intermediate	49000	40000	44000	42000	37000	45000	59000	26000	38000	55000
	fruit fillings	intermediate	40000	55000	30000	42000	33000	35000	48000	42000	37000	41000
	fruit fillings	high	536000	409000	565000	435000	570000	527000	344000	354000	524000	490000
	fruit fillings	high	504000	437000	519000	473000	489000	543000	398000	430000	505000	405000

B

(Food) Category	2
(Food) Type	1

			Reference method					Alternative method				
Sample Name	(Food) item	Level	rep 1	rep 2	rep 3	rep 4	rep 5	rep 1	rep 2	rep 3	rep 4	rep 5
	dry-fermented sausages	low	1060	1660	1310	1560	1320	1330	1020	1020	1530	1460
	dry-fermented sausages	low	670	790	760	1020	840	1070	1480	1040	970	1000
	dry-fermented sausages	intermediate	520000	310000	510000	270000	370000	317000	190000	450000	230000	366000
	dry-fermented sausages	intermediate	450000	485000	454000	341000	356000	450000	284000	403000	327000	319000
	dry-fermented sausages	high	7100000	8850000	10100000	7600000	8160000	6200000	7490000	8700000	4900000	6300000
	dry-fermented sausages	high	6300000	7870000	9800000	7140000	7350000	6920000	6650000	7640000	5340000	6400000

C

(Food) Category	3
(Food) Type	2

			Reference method					Alternative method				
Sample Name	(Food) item	Level	rep 1	rep 2	rep 3	rep 4	rep 5	rep 1	rep 2	rep 3	rep 4	rep 5
	dry-cured meat products	low	600	750	1040	1130	850	1190	860	960	990	1430
	dry-cured meat products	low	1030	920	700	750	770	1050	1190	1259	1040	910
	dry-cured meat products	interm.	100000	54000	76000	61000	65000	79000	81000	51000	65000	65000
	dry-cured meat products	interm.	82000	74000	75700	90000	95000	68000	58000	67000	65000	64500
	dry-cured meat products	high	1640000	1820000	1680000	2884000	1900000	1895000	2256000	2584000	2525000	1412000
	dry-cured meat products	high	2092000	1610000	1840000	1930000	1741000	1996000	2010000	2315000	2198000	1659000

D

(Food) Category	4
(Food) Type	2

			Reference method					Alternative method				
Sample Name	(Food) item	Level	rep 1	rep 2	rep 3	rep 4	rep 5	rep 1	rep 2	rep 3	rep 4	rep 5
	fruit jams	low	1300	1850	1540	1390	1290	1230	1310	1540	1680	1700
	fruit jams	low	1740	1250	1530	1250	1580	1640	1560	1160	1230	1360
	fruit jams	interm.	45000	37000	33000	31000	33000	26000	32000	26000	28000	21000
	fruit jams	interm.	36500	31000	34400	38200	26000	27700	37000	30000	33000	27000
	fruit jams	high	295000	272000	271000	288000	265000	370000	401000	225000	278000	291000
	fruit jams	high	302000	333000	253000	250000	280000	329000	362000	283000	280000	295000

E

(Food) Category	5
(Food) Type	3

			Reference method					Alternative method				
Sample Name	(Food) item	Level	rep 1	rep 2	rep 3	rep 4	rep 5	rep 1	rep 2	rep 3	rep 4	rep 5
	flours	low	840	1410	1150	1480	940	930	1150	1230	880	1000
	flours	low	1200	900	920	1150	1370	1020	840	1400	1310	1330
	flours	interm.	104000	115000	87000	91000	138000	100000	66000	86000	118000	87000
	flours	interm.	96000	116000	85000	89000	116000	105000	96000	94000	110000	88000
	flours	high	830000	991000	1137000	920000	895000	835000	1011000	1063000	882000	950000
	flours	high	848000	935000	1040000	854000	937000	888000	970000	1010000	926000	880000

F

(Food) Category	6
(Food) Type	1

			Reference method					Alternative method				
Sample Name	(Food) item	Level	rep 1	rep 2	rep 3	rep 4	rep 5	rep 1	rep 2	rep 3	rep 4	rep 5
	candies	low	450	960	900	580	640	940	750	690	540	470
	candies	low	990	870	950	860	890	820	680	800	400	650
	candies	interm.	60000	60000	63000	50000	52000	72000	88000	58000	68000	50000
	candies	interm.	64000	66000	59000	52000	60000	67000	80000	67000	65000	55000
	candies	high	580000	680000	480000	480000	570000	640000	800000	800000	780000	710000
	candies	high	630000	720000	560000	590000	680000	610000	780000	770000	750000	760000

G

(Feed) Category	7
(Feed) Type	2

			Reference method					Alternative method				
Sample Name	(Feed) item	Level	rep 1	rep 2	rep 3	rep 4	rep 5	rep 1	rep 2	rep 3	rep 4	rep 5
	starter feeds	low	1010	1220	1360	870	1130	1320	1180	1020	1200	1260
	starter feeds	low	830	750	1000	1360	1210	840	1110	1340	1170	1420
	starter feeds	interm.	251000	165000	178000	181000	220000	290000	143000	211000	210000	190000
	starter feeds	interm.	228000	189000	221000	164000	193000	243000	171000	175000	185000	176000
	starter feeds	high	1370000	1500000	1110000	1580000	1310000	1610000	1350000	1620000	1490000	1570000
	starter feeds	high	1320000	1490000	1350000	1390000	1330000	1510000	1530000	1540000	1310000	1720000

Table S3. Raw data used for the accuracy profile assessment obtained using **DSBM-I** and DG18 media for the enumeration of **yeasts and moulds** across selected food and feed categories and types. (A) Raw data for Category 1, Type 2, (B) Raw data for Category 2, Type 1, (C) Raw data for Category 3, Type 2, (D) Raw data for Category 4, Type 2, (E) Raw data for Category 5, Type 3, (F) Raw data for Category 6, Type 1, (G) Raw data for Category 7, Type 2. The results are expressed as CFU/g. The food categories and types included in the study are listed in Table 1 of the manuscript. Interm.: intermediate; rep.: replicate.

According to **ISO 16140-2:2014**, the Probability for the Tolerance Interval (**Proba TI**) must be **80%** and the central value must be the **median.**

**Proba TI (beta):**	80%
**Central Value:**	Median

(Food) Category	1
(Food) Type	2

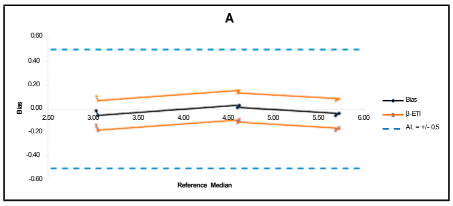

Sample Name	Reference central value	Bias	Lower β-ETI	Upper β-ETI	β-ETI compared to AL=±0.5 Acceptable	β-ETI compared to final AL Acceptable
	3.04	-0.016	-0.140	0.108	YES	YES
	3.06	-0.057	-0.181	0.067	YES	YES
	4.62	0.030	-0.094	0.154	YES	YES
	4.60	0.011	-0.113	0.135	YES	YES
	5.73	-0.039	-0.163	0.085	YES	YES
	5.69	-0.056	-0.180	0.068	YES	YES

	Reference method	Alternative method	SD repeatability of reference method <= 0.125	Final AL
SD Repeatability	0.075	0.086	YES	+/- 0.500

(Food) Category	2
(Food) Type	1

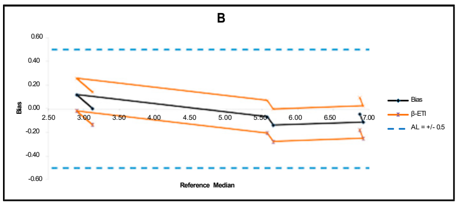

Sample Name	Reference Central value	Bias	Lower β-ETI	Upper β-ETI	β-ETI compared to AL=±0.5 Acceptable	β-ETI compared to final AL Acceptable
	3.12	0.003	-0.134	0.140	YES	YES
	2.90	0.119	-0.018	0.257	YES	YES
	5.57	-0.067	-0.204	0.070	YES	YES
	5.65	-0.139	-0.276	-0.002	YES	YES
	6.91	-0.112	-0.249	0.025	YES	YES
	6.87	-0.043	-0.181	0.094	YES	YES

	Reference method	Alternative method	SD repeatability of reference method <= 0.125	Final AL
SD Repeatability	0.081	0.095	YES	+/- 0.500

(Food) Category	3
(Food) Type	2

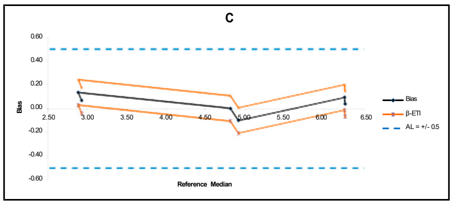

Sample Name	Reference Central value	Bias	Lower β-ETI	Upper β-ETI	β-ETI compared to AL=±0.5 Acceptable	β-ETI compared to final AL Acceptable
	2.93	0.066	-0.041	0.173	YES	YES
	2.89	0.135	0.028	0.242	YES	YES
	4.81	0.000	-0.107	0.107	YES	YES
	4.91	-0.101	-0.208	0.006	YES	YES
	6.26	0.093	-0.014	0.200	YES	YES
	6.26	0.038	-0.068	0.145	YES	YES

	Reference method	Alternative method	SD repeatability of reference method <= 0.125	Final AL
SD Repeatability	0.083	0.074	YES	+/- 0.500

(Food) Category	4
(Food) Type	2

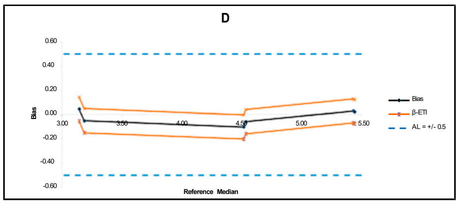

Sample Name	Reference Central value	Bias	Lower β-ETI	Upper β-ETI	β-ETI compared to AL=±0.5 Acceptable	β-ETI compared to final AL Acceptable
	3.14	0.045	-0.055	0.144	YES	YES
	3.18	-0.051	-0.151	0.048	YES	YES
	4.52	-0.104	-0.203	-0.004	YES	YES
	4.54	-0.059	-0.159	0.040	YES	YES
	5.43	0.029	-0.070	0.129	YES	YES
	5.45	0.023	-0.077	0.122	YES	YES

	Reference method	Alternative method	SD repeatability of reference method <= 0.125	Final AL
SD Repeatability	0.058	0.069	YES	+/- 0.500

(Food) Category	5
(Food) Type	3

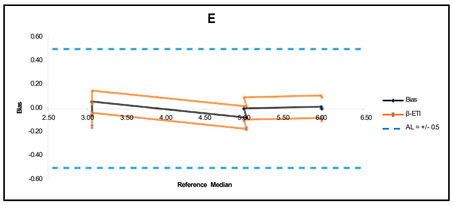

Sample Name	Reference Central value	Bias	Lower β-ETI	Upper β-ETI	β-ETI compared to AL=±0.5 Acceptable	β-ETI compared to final AL Acceptable
	3.06	-0.061	-0.155	0.033	YES	YES
	3.06	0.057	-0.037	0.150	YES	YES
	5.02	-0.078	-0.171	0.016	YES	YES
	4.98	0.000	-0.094	0.094	YES	YES
	5.96	0.014	-0.080	0.108	YES	YES
	5.97	-0.004	-0.098	0.090	YES	YES

	Reference method	Alternative method	SD repeatability of reference method <= 0.125	Final AL
SD Repeatability	0.073	0.065	YES	+/- 0.500

(Food) Category	6
(Food) Type	1

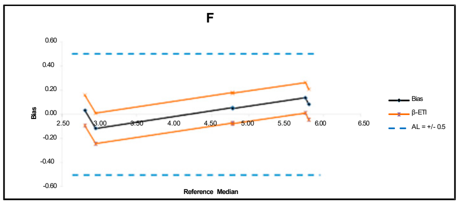

Sample Name	Reference Central value	Bias	Lower β-ETI	Upper β-ETI	β-ETI compared to AL=±0.5 Acceptable	β-ETI compared to final AL Acceptable
	2.81	0.033	-0.093	0.158	YES	YES
	2.95	-0.117	-0.242	0.009	YES	YES
	4.78	0.054	-0.071	0.180	YES	YES
	4.78	0.048	-0.078	0.174	YES	YES
	5.76	0.136	0.011	0.262	YES	YES
	5.80	0.081	-0.044	0.207	YES	YES

	Reference method	Alternative method	SD repeatability of reference method <= 0.125	Final AL
SD Repeatability	0.069	0.087	YES	+/- 0.500

(Feed) Category	7
(Feed) Type	2

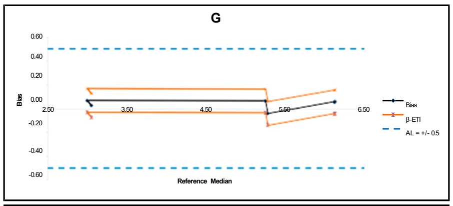

Sample Name	Reference Central value	Bias	Lower β-ETI	Upper β-ETI	β-ETI compared to AL=±0.5 Acceptable	β-ETI compared to final AL Acceptable
	3.05	0.026	-0.074	0.126	YES	YES
	3.00	0.068	-0.031	0.168	YES	YES
	5.26	0.065	-0.035	0.164	YES	YES
	5.29	-0.040	-0.140	0.060	YES	YES
	6.14	0.059	-0.040	0.159	YES	YES
	6.13	0.054	-0.045	0.154	YES	YES

	Reference method	Alternative method	SD repeatability of reference method <= 0.125	Final AL
SD Repeatability	0.071	0.069	YES	+/- 0.500

Figure S1. Bias and β-ETI limits, compliance with predefined acceptability criteria, and graphical presentation of the accuracy profile (AP) study results for the **DSBM-I** medium variation. **The results presented in Figure S1 were derived from the raw data reported in Table S3**. The panels in Figure S1 show the results for the following food and feed categories and types: (A) Category 1, Type 2, (B) Category 2, Type 1, (C) Category 3, Type 2, (D) Category 4, Type 2, (E) Category 5, Type 3, (F) Category 6, Type 1, (G) Category 7, Type 2. Bias is expressed as $\log_{10}$ CFU/g. Black lines indicate bias, orange lines indicate the 80% β-expectation tolerance intervals (β-ETI), and blue dashed lines indicate the acceptability limits (AL) of ±0.5 $\log_{10}$ CFU/g. Two samples were analyzed at each contamination level (low, intermediate, and high), with five replicate determinations performed per sample. The categories and selected product types included in the study are listed in Table 1 of the manuscript.

A

(Food) Category	1
(Food) Type	2

			Reference method					Alternative method				
Sample Name	(Food) item	Level	rep 1	rep 2	rep 3	rep 4	rep 5	rep 1	rep 2	rep 3	rep 4	rep 5
	fruit fillings	low	1380	1400	1680	1760	1580	1040	1590	1160	1480	1390
	fruit fillings	low	1040	1020	1400	950	1120	1470	880	1320	1450	1190
	fruit fillings	interm.	110000	126000	140000	90000	137000	135000	110000	159000	126000	112000
	fruit fillings	interm.	103000	185000	184000	136000	165000	151000	153000	190000	188000	135000
	fruit fillings	high	432000	704000	688000	762000	678000	632000	692000	846000	654000	518000
	fruit fillings	high	456000	642000	710000	564000	610000	698000	589600	761200	810400	603000

B

(Food) Category	2
(Food) Type	1

			Reference method					Alternative method				
Sample Name	(Food) item	Level	rep 1	rep 2	rep 3	rep 4	rep 5	rep 1	rep 2	rep 3	rep 4	rep 5
	dry-fermented sausages	low	940	1380	1230	1300	1530	1220	1190	970	720	1710
	dry-fermented sausages	low	1730	1230	1500	880	1120	1060	1070	1420	1210	1140
	dry-fermented sausages	interm.	775000	450000	590000	548000	626000	560000	360000	540000	480000	427000
	dry-fermented sausages	interm.	491000	498000	542000	489000	493000	674000	526000	568000	525000	595000
	dry-fermented sausages	high	6680000	6916000	5828000	5380000	6260000	4720000	6340000	3860000	7380000	4600000
	dry-fermented sausages	high	7036000	6400000	4800000	9920000	5740000	6800000	7300000	4400000	5550000	6400000

C

(Food) Category	3
(Food) Type	2

			Reference method					Alternative method				
Sample Name	(Food) item	Level	rep 1	rep 2	rep 3	rep 4	rep 5	rep 1	rep 2	rep 3	rep 4	rep 5
	dry-cured meat products	low	1890	1090	1570	1560	1600	840	1440	1370	670	1480
	dry-cured meat products	low	1300	1250	1180	1220	1710	1110	900	1430	1390	1140
	dry-cured meat products	interm.	110000	125000	127000	132000	143000	122000	130000	147000	130000	110000
	dry-cured meat products	interm.	114000	134000	134000	118000	122000	149000	155000	129000	147000	134000
	dry-cured meat products	high	4440000	9040000	6000000	6180000	4860000	3820000	5200000	6380000	5800000	5400000
	dry-cured meat products	high	5320000	7400000	5560000	3440000	6500000	5290000	6550000	5550000	4000000	6100000

D

(Food) Category	4
(Food) Type	2

			Reference method					Alternative method				
Sample Name	(Food) item	Level	rep 1	rep 2	rep 3	rep 4	rep 5	rep 1	rep 2	rep 3	rep 4	rep 5
	fruit jams	low	1300	940	1830	1140	1360	900	1030	1300	910	1560
	fruit jams	low	1540	1420	1370	1320	1520	1490	1080	1260	1220	1550
	fruit jams	interm.	125000	138000	169000	110000	142000	137000	138000	130000	139000	118000
	fruit jams	interm.	135000	147000	181000	135000	154000	174000	187000	173000	163000	153000
	fruit jams	high	554000	700000	896000	652000	884000	580000	430000	430000	690000	580000
	fruit jams	high	520000	808000	720000	704000	880000	500000	500000	700000	460000	690000

E

(Food) Category	5
(Food) Type	3

			Reference method					Alternative method				
Sample Name	(Food) item	Level	rep 1	rep 2	rep 3	rep 4	rep 5	rep 1	rep 2	rep 3	rep 4	rep 5
	flours	low	940	1160	1290	1570	960	1240	1160	1000	1170	1420
	flours	low	1450	1040	1050	1250	1390	1200	840	970	1080	1160
	flours	interm.	115000	95000	90000	138000	130000	83000	71000	81000	79000	81000
	flours	interm.	100000	99000	107000	127000	114000	96000	89000	103000	93000	105000
	flours	high	648000	444000	520000	588000	458000	320000	300000	360000	510000	410000
	flours	high	620000	420000	620000	520000	554000	410000	600000	410000	390000	450000

F

(Food) Category 6	6
(Food) Type 6	1

			Reference method					Alternative method				
Sample Name	(Food) item	Level	rep 1	rep 2	rep 3	rep 4	rep 5	rep 1	rep 2	rep 3	rep 4	rep 5
	candies	low	970	1280	1200	840	760	1330	1460	1720	810	1650
	candies	low	1350	1040	940	1250	1270	1250	1170	1300	1000	1200
	candies	interm.	179000	140000	173000	170000	186000	138000	136000	111000	152000	129000
	candies	interm.	168000	147000	186000	164000	168000	174000	140000	137000	164000	156000
	candies	high	590000	680000	710000	630000	680000	770000	600000	780000	620000	590000
	candies	high	700000	800000	900000	600000	950000	800000	700000	700000	720000	550000

G

(Feed) Category	7
(Feed) Type	2

			Reference method					Alternative method				
Sample Name	(Feed) item	Level	rep 1	rep 2	rep 3	rep 4	rep 5	rep 1	rep 2	rep 3	rep 4	rep 5
	starter feeds	low	1200	800	900	1040	1050	1220	1190	1310	950	1270
	starter feeds	low	950	1170	1200	1100	890	830	1000	1200	1210	1080
	starter feeds	interm.	150000	130000	111000	150000	142000	180000	152000	136000	190000	150000
	starter feeds	interm.	193000	141000	135000	208000	170000	214000	115000	159000	173000	182000
	starter feeds	high	2700000	3010000	4900000	3900000	4700000	2100000	2700000	2850000	4040000	3130000
	starter feeds	high	3130000	2120000	4170000	3100000	4110000	3120000	2110000	3680000	3590000	4000000

Table S4. Raw data used for the accuracy profile assessment obtained using **DSBM-II** and DG18 media for the enumeration of **yeasts and moulds** across selected food and feed categories and types. (A) Raw data for Category 1, Type 2, (B) Raw data for Category 2, Type 1, (C) Raw data for Category 3, Type 2, (D) Raw data for Category 4, Type 2, (E) Raw data for Category 5, Type 3, (F) Raw data for Category 6, Type 1, (G) Raw data for Category 7, Type 2. The results are expressed as CFU/g. The food categories and types included in the study are listed in Table 1 of the manuscript. Interm.: intermediate; rep.: replicate.

According to **ISO 16140-2:2014**, the Probability for the Tolerance Interval (**Proba TI**) must be **80%** and the central value must be the **median.**

**Proba TI (beta):**	80%
**Central Value:**	Median

(Food) Category	1
(Food) Type	2

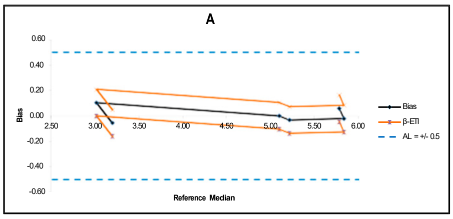

Sample Name	Reference central value	Bias	Lower β-ETI	Upper β-ETI	β-ETI compared to AL=±0.5 Acceptable	β-ETI compared to final AL Acceptable
	3.20	-0.056	-0.161	0.050	YES	YES
	3.02	0.104	-0.002	0.209	YES	YES
	5.10	0.000	-0.105	0.105	YES	YES
	5.22	-0.033	-0.138	0.073	YES	YES
	5.84	-0.022	-0.127	0.083	YES	YES
	5.79	0.059	-0.047	0.164	YES	YES

	Reference method	Alternative method	SD repeatability of reference method <= 0.125	Final AL
SD Repeatability	0.081	0.073	YES	+/- 0.500

(Food) Category	2
(Food) Type	1

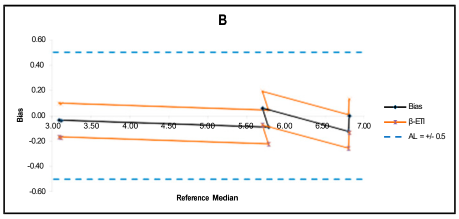

Sample Name	Reference Central value	Bias	Lower β-ETI	Upper β-ETI	β-ETI compared to AL=±0.5 Acceptable	β-ETI compared to final AL Acceptable
	3.11	-0.038	-0.171	0.094	YES	YES
	3.09	-0.033	-0.166	0.100	YES	YES
	5.77	-0.090	-0.222	0.043	YES	YES
	5.69	0.062	-0.071	0.194	YES	YES
	6.80	-0.123	-0.255	0.010	YES	YES
	6.81	0.000	-0.133	0.133	YES	YES

	Reference method	Alternative method	SD repeatability of reference method <= 0.125	Final AL
SD Repeatability	0.084	0.092	YES	+/- 0.500

(Food) Category	3
(Food) Type	2

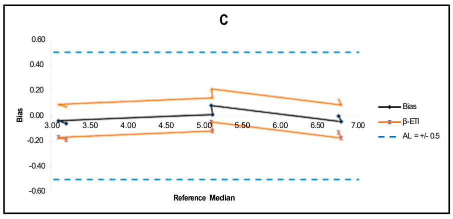

Sample Name	Reference Central value	Bias	Lower β-ETI	Upper β-ETI	β-ETI compared to AL=±0.5 Acceptable	β-ETI compared to final AL Acceptable
	3.20	-0.059	-0.189	0.071	YES	YES
	3.10	-0.040	-0.170	0.090	YES	YES
	5.10	0.010	-0.120	0.140	YES	YES
	5.09	0.081	-0.049	0.211	YES	YES
	6.78	-0.046	-0.176	0.084	YES	YES
	6.75	-0.001	-0.131	0.129	YES	YES

	Reference method	Alternative method	SD repeatability of reference method <= 0.125	Final AL
SD Repeatability	0.086	0.090	YES	+/- 0.500

(Food) Category	4
(Food) Type	2

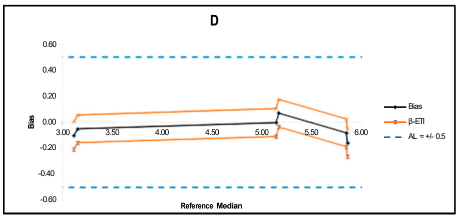

Sample Name	Reference Central value	Bias	Lower β-ETI	Upper β-ETI	β-ETI compared to AL=±0.5 Acceptable	β-ETI compared to final AL Acceptable
	3.11	-0.101	-0.208	0.006	YES	YES
	3.15	-0.052	-0.159	0.055	YES	YES
	5.14	-0.003	-0.110	0.104	YES	YES
	5.17	0.071	-0.036	0.178	YES	YES
	5.85	-0.082	-0.188	0.025	YES	YES
	5.86	-0.158	-0.265	-0.052	YES	YES

	Reference method	Alternative method	SD repeatability of reference method <= 0.125	Final AL
SD Repeatability	0.077	0.074	YES	+/- 0.500

(Food) Category	Category 5
(Food) Type	Type 3

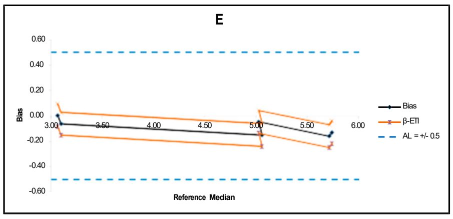

Sample Name	Reference Central value	Bias	Lower β-ETI	Upper β-ETI	β-ETI compared to AL=±0.5 Acceptable	β-ETI compared to final AL Acceptable
	3.06	0.004	-0.086	0.093	YES	YES
	3.10	-0.063	-0.153	0.026	YES	YES
	5.06	-0.152	-0.242	-0.063	YES	YES
	5.03	-0.047	-0.137	0.042	YES	YES
	5.72	-0.160	-0.249	-0.070	YES	YES
	5.74	-0.131	-0.220	-0.041	YES	YES

	Reference method	Alternative method	SD repeatability of reference method <= 0.125	Final AL
SD Repeatability	0.073	0.062	YES	+/- 0.500

(Food) Category	Category 6
(Food) Type	Type 1

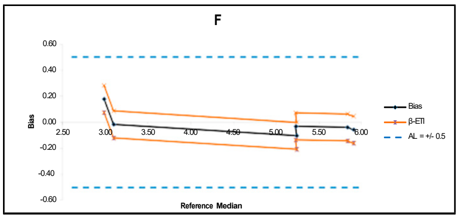

Sample Name	Reference Central value	Bias	Lower β-ETI	Upper β-ETI	β-ETI compared to AL=±0.5 Acceptable	β-ETI compared to final AL Acceptable
	2.99	0.178	0.074	0.282	YES	YES
	3.10	-0.018	-0.122	0.086	YES	YES
	5.24	-0.105	-0.208	-0.001	YES	YES
	5.23	-0.032	-0.136	0.072	YES	YES
	5.83	-0.040	-0.144	0.064	YES	YES
	5.90	-0.058	-0.162	0.046	YES	YES

	Reference method	Alternative method	SD repeatability of reference method <= 0.125	Final AL
SD Repeatability	0.065	0.072	YES	+/- 0.500

(Food) Category	Category 7
(Food) Type	Type 2

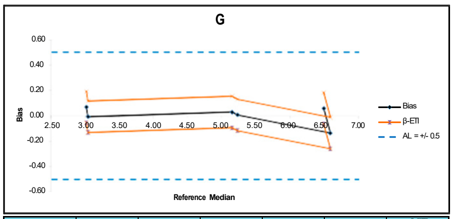

Sample Name	Reference Central value	Bias	Lower β-ETI	Upper β-ETI	β-ETI compared to AL=±0.5 Acceptable	β-ETI compared to final AL Acceptable
	3.02	0.069	-0.055	0.193	YES	YES
	3.04	-0.008	-0.132	0.116	YES	YES
	5.15	0.030	-0.095	0.154	YES	YES
	5.23	0.008	-0.117	0.132	YES	YES
	6.59	-0.136	-0.260	-0.012	YES	YES
	6.50	0.060	-0.065	0.184	YES	YES

Figure S2. Bias and β-ETI limits, compliance with predefined acceptability criteria, and graphical presentation of the accuracy profile (AP) study results for the **DSBM-II** medium variation. **The results presented in Figure S2 were derived from the raw data reported in Table S4.** The panels in Figure S2 show the results for the following food and feed categories and types: (A) Category 1, Type 2, (B) Category 2, Type 1, (C) Category 3, Type 2, (D) Category 4, Type 2, (E) Category 5, Type 3, (F) Category 6, Type 1, (G) Category 7, Type 2. Bias is expressed as $\log_{10}$ CFU/g. Black lines indicate bias, orange lines indicate the 80% β-expectation tolerance intervals (β-ETI), and blue dashed lines indicate the acceptability limits (AL) of ±0.5 $\log_{10}$ CFU/g. Two samples were analyzed at each contamination level (low, intermediate, and high), with five replicate determinations performed per sample. The categories and selected product types included in the study are listed in Table 1.

A

(Food) Category	1
(Food) Type	2

Sample Name	(Food) item	Level	Reference method rep 1	rep 2	rep 3	rep 4	rep 5	Alternative method rep 1	rep 2	rep 3	rep 4	rep 5
	fruit fillings	low	680	900	800	810	1230	770	1200	1150	960	1310
	fruit fillings	low	1200	1320	1150	1050	960	1110	1020	1150	1230	850
	fruit fillings	interm.	120000	136000	130000	114000	129000	100000	123000	150000	117000	110000
	fruit fillings	interm.	103000	149000	124000	149000	135000	165000	143000	142000	123000	116000
	fruit fillings	high	432000	704000	688000	762000	678000	706000	654000	650000	528000	828000
	fruit fillings	high	456000	642000	710000	664000	610000	640000	928000	700000	620000	624000

B

(Food) Category	2
(Food) Type	1

Sample Name	(Food) item	Level	Reference method rep 1	rep 2	rep 3	rep 4	rep 5	Alternative method rep 1	rep 2	rep 3	rep 4	rep 5
	dry-fermented sausages	low	1440	1320	990	1200	1860	800	1440	1260	1310	1600
	dry-fermented sausages	low	1790	1560	1600	920	1240	1300	1220	1390	950	1200
	dry-fermented sausages	interm.	775000	430000	590000	548000	626000	610000	370000	490000	480000	520000
	dry-fermented sausages	interm.	491000	478000	542000	489000	493000	555000	485000	576000	612000	631000
	dry-fermented sausages	high	5380000	7916000	6828000	5380000	6260000	5420000	6560000	8040000	5500000	4720000
	dry-fermented sausages	high	6036000	9100000	4800000	6920000	5740000	4200000	6400000	9140000	6200000	5460000

C

(Food) Category	3
(Food) Type	2

Sample Name	(Food) item	Level	Reference method rep 1	rep 2	rep 3	rep 4	rep 5	Alternative method rep 1	rep 2	rep 3	rep 4	rep 5
	dry-cured meat products	low	1350	1040	1100	1340	1040	730	1430	960	1330	800
	dry-cured meat products	low	1190	1140	1490	850	740	870	700	1130	760	700
	dry-cured meat products	interm.	110000	125000	127000	132000	143000	146000	132000	163000	120000	118000
	dry-cured meat products	interm.	114000	134000	134000	118000	122000	105000	130000	113000	116000	122000
	dry-cured meat products	high	4440000	9040000	6000000	6180000	5860000	4920000	6580000	6380000	5700000	5060000
	dry-cured meat products	high	5320000	7400000	5560000	5440000	6500000	6200000	6200000	6200000	5440000	6200000

D

(Food) Category	4
(Food) Type	2

Sample Name	(Food) item	Level	Reference method rep 1	rep 2	rep 3	rep 4	rep 5	Alternative method rep 1	rep 2	rep 3	rep 4	rep 5
	fruit jams	low	790	1400	1020	1060	830	900	1220	1510	1330	1540
	fruit jams	low	1700	1250	1430	1280	960	1460	960	1070	1090	1180
	fruit jams	interm.	90000	128000	149000	110000	127000	140000	140000	131000	140000	115000
	fruit jams	interm.	95000	135000	132000	135000	134000	145000	152000	161000	159000	133000
	fruit jams	high	554000	700000	796000	652000	714000	710000	684000	696000	720000	514000
	fruit jams	high	520000	768000	720000	674000	780000	700000	550000	520000	344000	656000

E

(Food) Category	5
(Food) Type	3

Sample Name	(Food) item	Level	Reference method rep 1	rep 2	rep 3	rep 4	rep 5	Alternative method rep 1	rep 2	rep 3	rep 4	rep 5
	flours	low	1270	1000	1080	1090	870	1100	1410	1430	1300	1050
	flours	low	1380	1280	740	1070	1230	1060	1150	1020	1150	1130
	flours	interm.	120000	90000	90000	138000	130000	105000	111000	74000	101000	92000
	flours	interm.	110000	99000	87000	146000	134000	103000	122000	88000	114000	107000
	flours	high	648000	344000	520000	588000	458000	500000	392000	590000	534000	572000
	flours	high	620000	420000	520000	520000	454000	554000	416400	617600	462800	490000

F

(Food) Category	6
(Food) Type	1

Sample Name	(Food) item	Level	Reference method rep 1	rep 2	rep 3	rep 4	rep 5	Alternative method rep 1	rep 2	rep 3	rep 4	rep 5
	candies	low	870	940	1000	1030	1500	890	1400	1100	990	1250
	candies	low	1490	1100	1020	1090	1280	1140	970	780	1310	1290
	candies	interm.	179000	140000	147000	170000	186000	112000	140000	160000	110000	141000
	candies	interm.	158000	117000	186000	134000	138000	130000	135000	143000	131000	132000
	candies	high	590000	680000	710000	630000	660000	680000	704000	664000	700000	680000
	candies	high	700000	1000000	900000	600000	950000	620000	640000	540000	530000	816000

G

(Feed) Category	7
(Feed) Type	2

Sample Name	(Food) item	Level	Reference method rep 1	rep 2	rep 3	rep 4	rep 5	Alternative method rep 1	rep 2	rep 3	rep 4	rep 5
	starter feeds	low	1460	1620	1190	1530	1630	950	1260	1400	1130	1850
	starter feeds	low	580	1010	1030	1090	1150	1800	1260	1040	1820	1310
	starter feeds	interm.	150000	110000	111000	150000	142000	139000	68000	109000	140000	138500
	starter feeds	interm.	193000	141000	135000	208000	170000	179000	163000	158000	167000	134000
	starter feeds	high	2700000	3010000	4900000	3900000	4700000	2195000	2320000	2730000	3897000	3290000
	starter feeds	high	3130000	2120000	2170000	3100000	4110000	3620000	3110000	4150000	3110000	4940000

Table S5. Raw data used for the accuracy profile assessment obtained using **DSBM-III** and DG18 media for the enumeration of **yeasts and moulds** across selected food and feed categories and types. (A) Raw data for Category 1, Type 2, (B) Raw data for Category 2, Type 1, (C) Raw data for Category 3, Type 2, (D) Raw data for Category 4, Type 2, (E) Raw data for Category 5, Type 3, (F) Raw data for Category 6, Type 1, (G) Raw data for Category 7, Type 2. The results are expressed as CFU/g. The food categories and types included in the study are listed in Table 1 of the manuscript. Interm.: intermediate; rep.: replicate.

According to **ISO 16140-2:2014**, the Probability for the Tolerance Interval (**Proba TI**) must be **80%** and the central value must be the **median.**

**Proba TI (beta):**	80%
**Central Value:**	Median

(Food) Category	1
(Food) Type	2

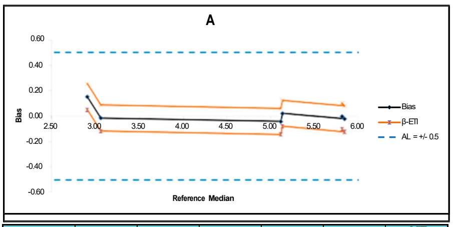

Sample Name	Reference central value	Bias	Lower β-ETI	Upper β-ETI	β-ETI compared to AL=±0.5 Acceptable	β-ETI compared to final AL Acceptable
	2.91	0.152	0.050	0.255	YES	YES
	3.06	-0.015	-0.118	0.087	YES	YES
	5.11	-0.042	-0.145	0.060	YES	YES
	5.13	0.022	-0.081	0.124	YES	YES
	5.84	-0.022	-0.125	0.080	YES	YES
	5.81	-0.001	-0.104	0.101	YES	YES

	Reference method	Alternative method	SD repeatability of reference method <= 0.125	Final AL
SD Repeatability	0.074	0.071	YES	+/- 0.500

(Food) Category	2
(Food) Type	1

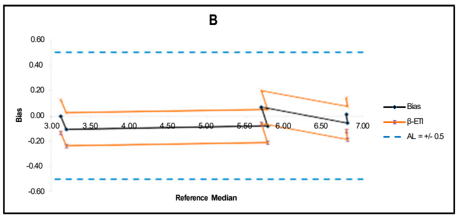

Sample Name	Reference Central value	Bias	Lower β-ETI	Upper β-ETI	β-ETI compared to AL=±0.5 Acceptable	β-ETI compared to final AL Acceptable
	3.12	-0.003	-0.133	0.127	YES	YES
	3.19	-0.107	-0.237	0.023	YES	YES
	5.77	-0.081	-0.211	0.049	YES	YES
	5.69	0.069	-0.061	0.199	YES	YES
	6.80	-0.056	-0.186	0.074	YES	YES
	6.78	0.012	-0.118	0.142	YES	YES

	Reference method	Alternative method	SD repeatability of reference method <= 0.125	Final AL
SD Repeatability	0.090	0.090	YES	+/- 0.500

(Food) Category	3
(Food) Type	2

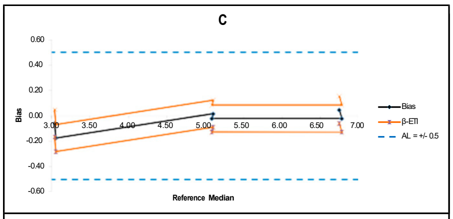

Sample Name	Reference Central value	Bias	Lower β-ETI	Upper β-ETI	β-ETI compared to AL=±0.5 Acceptable	β-ETI compared to final AL Acceptable
	3.04	-0.059	-0.166	0.048	YES	YES
	3.06	-0.176	-0.283	-0.069	YES	YES
	5.10	0.017	-0.090	0.124	YES	YES
	5.09	-0.022	-0.129	0.085	YES	YES
	6.78	-0.022	-0.129	0.085	YES	YES
	6.75	0.047	-0.060	0.154	YES	YES

	Reference method	Alternative method	SD repeatability of reference method <= 0.125	Final AL
SD Repeatability	0.078	0.074	YES	+/- 0.500

(Food) Category	4
(Food) Type	2

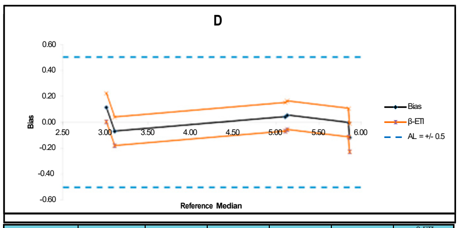

Sample Name	Reference Central value	Bias	Lower β-ETI	Upper β-ETI	β-ETI compared to AL=±0.5 Acceptable	β-ETI compared to final AL Acceptable
	3.01	0.115	0.006	0.225	YES	YES
	3.11	-0.070	-0.179	0.040	YES	YES
	5.10	0.042	-0.067	0.152	YES	YES
	5.13	0.055	-0.055	0.164	YES	YES
	5.85	-0.002	-0.112	0.107	YES	YES
	5.86	-0.117	-0.227	-0.007	YES	YES

	Reference method	Alternative method	SD repeatability of reference method <= 0.125	Final AL
SD Repeatability	0.079	0.076	YES	+/- 0.500

(Food) Category	5
(Food) Type	3

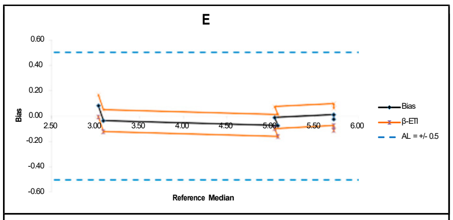

Sample Name	Reference Central value	Bias	Lower β-ETI	Upper β-ETI	β-ETI compared to AL=±0.5 Acceptable	β-ETI compared to final AL Acceptable
	3.03	0.081	-0.006	0.167	YES	YES
	3.09	-0.037	-0.123	0.050	YES	YES
	5.08	-0.075	-0.161	0.012	YES	YES
	5.04	-0.012	-0.099	0.075	YES	YES
	5.72	0.012	-0.075	0.098	YES	YES
	5.72	-0.026	-0.112	0.061	YES	YES

	Reference method	Alternative method	SD repeatability of reference method <= 0.125	Final AL
SD Repeatability	0.089	0.060	YES	+/- 0.500

(Food) Category	6
(Food) Type	1

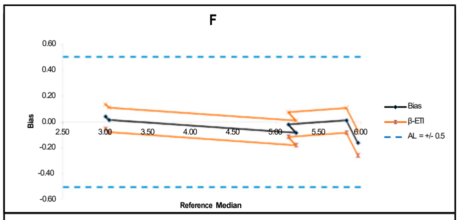

Sample Name	Reference Central value	Bias	Lower β-ETI	Upper β-ETI	β-ETI compared to AL=±0.5 Acceptable	β-ETI compared to final AL Acceptable
	3.00	0.041	-0.054	0.137	YES	YES
	3.04	0.016	-0.080	0.111	YES	YES
	5.23	-0.084	-0.180	0.011	YES	YES
	5.14	-0.019	-0.115	0.076	YES	YES
	5.82	0.013	-0.082	0.108	YES	YES
	5.95	-0.162	-0.257	-0.067	YES	YES

	Reference method	Alternative method	SD repeatability of reference method <= 0.125	Final AL
SD Repeatability	0.073	0.066	YES	+/- 0.500

(Food) Category	7
(Food) Type	2

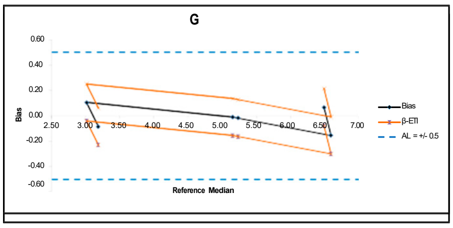

Sample Name	Reference Central value	Bias	Lower β-ETI	Upper β-ETI	β-ETI compared to AL=±0.5 Acceptable	β-ETI compared to final AL Acceptable
	3.18	-0.084	-0.230	0.061	YES	YES
	3.01	0.104	-0.041	0.250	YES	YES
	5.15	-0.011	-0.157	0.135	YES	YES
	5.23	-0.018	-0.164	0.128	YES	YES

	Reference method	Alternative method	SD repeatability of reference method <= 0.125	Final AL
SD Repeatability	0.098	0.101	YES	+/- 0.500

Figure S3. Bias and β-ETI limits, compliance with predefined acceptability criteria, and graphical presentation of the accuracy profile (AP) study results for the **DSBM-III** medium variation. **The results presented in Figure S3 were derived from the raw data reported in Table S5**. The panels in Figure S3 show the results for the following food and feed categories and types: (A) Category 1, Type 2, (B) Category 2, Type 1, (C) Category 3, Type 2, (D) Category 4, Type 2, (E) Category 5, Type 3, (F) Category 6, Type 1, (G) Category 7, Type 2. Bias is expressed as log_{10} CFU/g. Black lines indicate bias, orange lines indicate the 80% β-expectation tolerance intervals (β-ETI), and blue dashed lines indicate the acceptability limits (AL) of ±0.5 log_{10} CFU/g. Two samples were analyzed at each contamination level (low, intermediate, and high), with five replicate determinations performed per sample. The categories and selected product types included in the study are listed in Table 1 of the manuscript.

According to **ISO 16140-2:2014**, the Probability for the Tolerance Interval (**Proba TI**) must be **80%** and the central value must be the **median.**

**Proba TI (beta):**	80%
**Central Value:**	Median

(Food) Category	1
(Food) Type	2

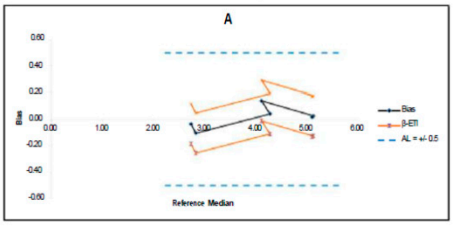

Sample Name	Reference central value	Bias	Lower β-ETI	Upper β-ETI	β-ETI compared to AL=±0.5 Acceptable	β-ETI compared to final AL Acceptable
	2.73	-0.033	-0.185	0.118	YES	YES
	2.83	-0.102	-0.253	0.050	YES	YES
	4.28	0.043	-0.108	0.195	YES	YES
	4.11	0.141	-0.010	0.293	YES	YES
	5.13	0.028	-0.124	0.180	YES	YES
	5.10	0.020	-0.132	0.172	YES	YES

	Reference method	Alternative method	SD repeatability of reference method <= 0.125	Final AL
SD Repeatability	0.103	0.105	YES	+/- 0.500

(Food) Category	2
(Food) Type	1

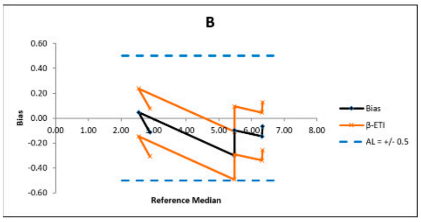

Sample Name	Reference Central value	Bias	Lower β-ETI	Upper β-ETI	β-ETI compared to AL=±0.5 Acceptable	β-ETI compared to final AL Acceptable
	2.89	-0.114	-0.306	0.078	YES	YES
	2.54	0.047	-0.145	0.239	YES	YES
	5.48	-0.301	-0.493	-0.109	YES	YES
	5.48	-0.097	-0.289	0.095	YES	YES
	6.32	-0.146	-0.338	0.046	YES	YES
	6.34	-0.064	-0.256	0.128	YES	YES

	Reference method	Alternative method	SD repeatability of reference method <= 0.125	Final AL
SD Repeatability	0.109	0.133	YES	+/- 0.500

(Food) Category	3
(Food) Type	2

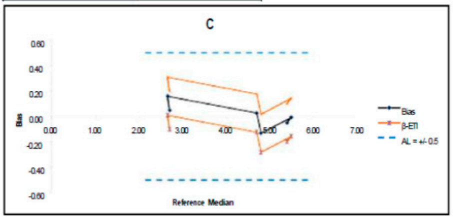

Sample Name	Reference Central value	Bias	Lower β-ETI	Upper β-ETI	β-ETI compared to AL=±0.5 Acceptable	β-ETI compared to final AL Acceptable
	2.70	0.049	-0.101	0.199	YES	YES
	2.65	0.160	0.010	0.310	YES	YES
	4.69	0.026	-0.124	0.176	YES	YES
	4.79	-0.132	-0.282	0.018	YES	YES
	5.48	-0.007	-0.157	0.143	YES	YES
	5.38	-0.050	-0.200	0.101	YES	YES

	Reference method	Alternative method	SD repeatability of reference method <= 0.125	Final AL
SD Repeatability	0.108	0.104	YES	+/- 0.500

(Food) Category	4
(Food) Type	2

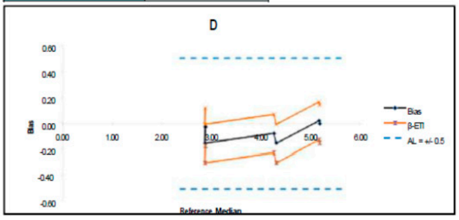

Sample Name	Reference Central value	Bias	Lower β-ETI	Upper β-ETI	β-ETI compared to AL=±0.5 Acceptable	β-ETI compared to final AL Acceptable
	2.87	-0.030	-0.176	0.115	YES	YES
	2.86	-0.156	-0.302	-0.010	YES	YES
	4.26	-0.079	-0.225	0.067	YES	YES
	4.30	-0.155	-0.301	-0.009	YES	YES
	5.16	0.018	-0.128	0.163	YES	YES
	5.18	-0.006	-0.152	0.140	YES	YES

	Reference method	Alternative method	SD repeatability of reference method <= 0.125	Final AL
SD Repeatability	0.102	0.101	YES	+/- 0.500

(Food) Category	5
(Food) Type	3

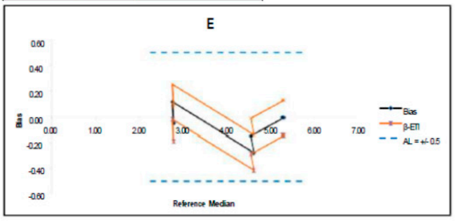

Sample Name	Reference Central value	Bias	Lower β-ETI	Upper β-ETI	β-ETI compared to AL=±0.5 Acceptable	β-ETI compared to final AL Acceptable
	2.78	-0.054	-0.191	0.083	YES	YES
	2.75	0.115	-0.022	0.252	YES	YES
	4.60	-0.280	-0.417	-0.143	YES	YES
	4.53	-0.151	-0.288	-0.014	YES	YES
	5.29	-0.009	-0.146	0.128	YES	YES
	5.25	-0.007	-0.145	0.130	YES	YES

	Reference method	Alternative method	SD repeatability of reference method <= 0.125	Final AL
SD Repeatability	0.102	0.095	YES	+/- 0.500

(Food) Category	6
(Food) Type	1

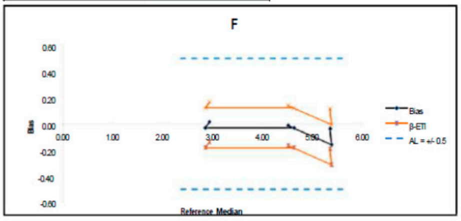

Sample Name	Reference Central value	Bias	Lower β-ETI	Upper β-ETI	β-ETI compared to AL=±0.5 Acceptable	β-ETI compared to final AL Acceptable
	2.94	0.015	-0.138	0.168	YES	YES
	2.85	-0.032	-0.185	0.121	YES	YES
	4.63	-0.031	-0.184	0.122	YES	YES
	4.52	-0.013	-0.166	0.140	YES	YES
	5.39	-0.160	-0.314	-0.007	YES	YES
	5.38	-0.037	-0.190	0.116	YES	YES

	Reference method	Alternative method	SD repeatability of reference method <= 0.125	Final AL
SD Repeatability	0.088	0.106	YES	+/- 0.500

(Feed) Category	7
(Feed) Type	2

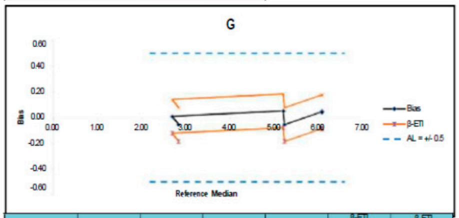

Sample Name	Reference Central value	Bias	Lower β-ETI	Upper β-ETI	β-ETI compared to AL=±0.5 Acceptable	β-ETI compared to final AL Acceptable
	2.83	-0.054	-0.186	0.077	YES	YES
	2.69	0.009	-0.123	0.140	YES	YES
	5.20	0.051	-0.080	0.183	YES	YES
	5.23	-0.054	-0.186	0.077	YES	YES
	6.08	0.048	-0.083	0.179	YES	YES
	6.08	0.041	-0.090	0.173	YES	YES

	Reference method	Alternative method	SD repeatability of reference method <= 0.125	Final AL
SD Repeatability	0.087	0.091	YES	+/- 0.500

Figure S4. Bias and β-ETI limits, compliance with predefined acceptability criteria, and graphical presentation of the accuracy profile (AP) study results for **moulds** enumeration using the **DSBM-I** medium variation. The panels in Figure S4 show the results for the following food and feed categories and types: (A) Category 1, Type 2, (B) Category 2, Type 1, (C) Category 3, Type 2, (D) Category 4, Type 2, (E) Category 5, Type 3, (F) Category 6, Type 1, (G) Category 7, Type 2. Bias is expressed as $\log_{10}$ CFU/g. Black lines indicate bias, orange lines indicate the 80% β-expectation tolerance intervals (β-ETI), and blue dashed lines indicate the acceptability limits (AL) of ±0.5 $\log_{10}$ CFU/g. Two samples were analyzed at each contamination level (low, intermediate, and high), with five replicate determinations performed per sample. The categories and selected product types included in the study are listed in Table 1 of the manuscript.

According to **ISO 16140-2:2014**, the Probability for the Tolerance Interval (**Proba TI**) must be **80%** and the central value must be the **median.**

**Proba TI (beta):**	80%
**Central Value:**	Median

(Food) Category	1
(Food) Type	2

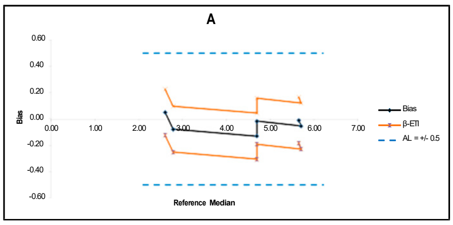

Sample Name	Reference central value	Bias	Lower β-ETI	Upper β-ETI	β-ETI compared to AL=±0.5 Acceptable	β-ETI compared to final AL Acceptable
	2.60	0.051	-0.124	0.226	YES	YES
	2.79	-0.078	-0.252	0.097	YES	YES
	4.70	-0.131	-0.305	0.044	YES	YES
	4.70	-0.018	-0.192	0.157	YES	YES
	5.71	-0.054	-0.229	0.120	YES	YES
	5.65	-0.010	-0.184	0.165	YES	YES

	Reference method	Alternative method	SD repeatability of reference method <= 0.125	Final AL
SD Repeatability	0.112	0.121	YES	+/- 0.500

(Food) Category	2
(Food) Type	1

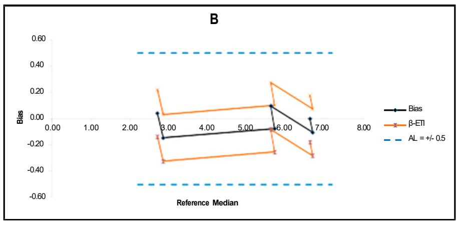

Sample Name	Reference Central value	Bias	Lower β-ETI	Upper β-ETI	β-ETI compared to AL=±0.5 Acceptable	β-ETI compared to final AL Acceptable
	2.70	0.041	-0.136	0.219	YES	YES
	2.85	-0.146	-0.324	0.031	YES	YES
	5.70	-0.076	-0.253	0.102	YES	YES
	5.60	0.097	-0.081	0.274	YES	YES
	6.67	-0.104	-0.281	0.074	YES	YES
	6.60	0.000	-0.178	0.178	YES	YES

	Reference method	Alternative method	SD repeatability of reference method <= 0.125	Final AL
SD Repeatability	0.120	0.123	YES	+/- 0.500

(Food) Category	3
(Food) Type	2

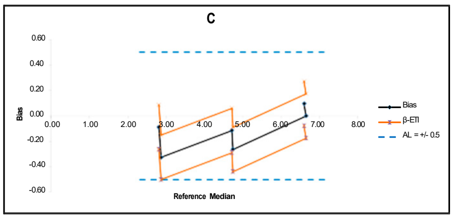

Sample Name	Reference Central value	Bias	Lower β-ETI	Upper β-ETI	β-ETI compared to AL=±0.5 Acceptable	β-ETI compared to final AL Acceptable
	2.81	-0.089	-0.263	0.086	YES	YES
	2.87	-0.325	-0.500	-0.150	YES	YES
	4.72	-0.114	-0.289	0.061	YES	YES
	4.74	-0.263	-0.438	-0.089	YES	YES
	6.65	0.000	-0.175	0.175	YES	YES
	6.60	0.097	-0.078	0.272	YES	YES

	Reference method	Alternative method	SD repeatability of reference method <= 0.125	Final AL
SD Repeatability	0.120	0.121	YES	+/- 0.500

(Food) Category	4
(Food) Type	2

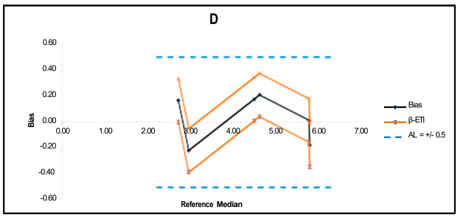

Sample Name	Reference Central value	Bias	Lower β-ETI	Upper β-ETI	β-ETI compared to AL=±0.5 Acceptable	β-ETI compared to final AL Acceptable
	2.71	0.167	0.000	0.335	YES	YES
	2.95	-0.217	-0.384	-0.050	YES	YES
	4.48	0.176	0.009	0.344	YES	YES
	4.60	0.211	0.043	0.378	YES	YES
	5.76	0.015	-0.153	0.182	YES	YES
	5.78	-0.176	-0.344	-0.009	YES	YES

	Reference method	Alternative method	SD repeatability of reference method <= 0.125	Final AL
SD Repeatability	0.118	0.116	YES	+/- 0.500

(Food) Category	5
(Food) Type	3

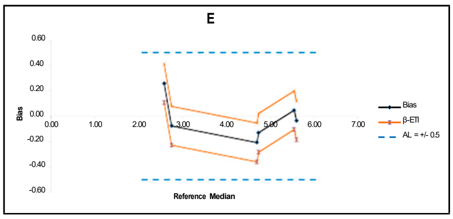

Sample Name	Reference Central value	Bias	Lower β-ETI	Upper β-ETI	β-ETI compared to AL=±0.5 Acceptable	β-ETI compared to final AL Acceptable
	2.58	0.259	0.106	0.412	YES	YES
	2.75	-0.076	-0.229	0.077	YES	YES
	4.70	-0.208	-0.361	-0.055	YES	YES
	4.73	-0.130	-0.283	0.023	YES	YES
	5.54	0.047	-0.106	0.200	YES	YES
	5.60	-0.034	-0.187	0.119	YES	YES

	Reference method	Alternative method	SD repeatability of reference method <= 0.125	Final AL
SD Repeatability	0.117	0.106	YES	+/- 0.500

(Food) Category	6
(Food) Type	1

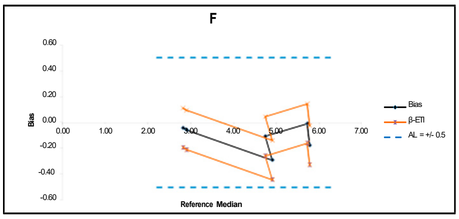

Sample Name	Reference Central value	Bias	Lower β-ETI	Upper β-ETI	β-ETI compared to AL=±0.5 Acceptable	β-ETI compared to final AL Acceptable
	2.82	-0.041	-0.193	0.110	YES	YES
	2.90	-0.058	-0.210	0.094	YES	YES
	4.90	-0.290	-0.442	-0.139	YES	YES
	4.74	-0.107	-0.258	0.045	YES	YES
	5.72	-0.008	-0.160	0.143	YES	YES
	5.78	-0.176	-0.328	-0.025	YES	YES

	Reference method	Alternative method	SD repeatability of reference method <= 0.125	Final AL
SD Repeatability	0.110	0.105	YES	+/- 0.500

(Feed) Category	7
(Feed) Type	2

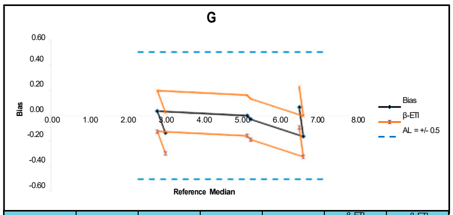

Sample Name	Reference Central value	Bias	Lower β-ETI	Upper β-ETI	β-ETI compared to AL=±0.5 Acceptable	β-ETI compared to final AL Acceptable
	2.98	-0.137	-0.297	0.023	YES	YES
	2.77	0.035	-0.125	0.196	YES	YES
	5.11	0.000	-0.160	0.160	YES	YES
	5.20	-0.028	-0.188	0.132	YES	YES
	6.58	-0.165	-0.325	-0.005	YES	YES
	6.48	0.067	-0.093	0.227	YES	YES

	Reference method	Alternative method	SD repeatability of reference method <= 0.125	Final AL
SD Repeatability	0.115	0.111	YES	+/- 0.500

Figure S5. Bias and β-ETI limits, compliance with predefined acceptability criteria, and graphical presentation of the accuracy profile (AP) study results for **moulds** enumeration using the **DSBM-II** medium variation. The panels in Figure S5 show the results for the following food and feed categories and types: (A) Category 1, Type 2, (B) Category 2, Type 1, (C) Category 3, Type 2, (D) Category 4, Type 2, (E) Category 5, Type 3, (F) Category 6, Type 1, (G) Category 7, Type 2. Bias is expressed as $\log_{10}$ CFU/g. Black lines indicate bias, orange lines indicate the 80% β-expectation tolerance intervals (β-ETI), and blue dashed lines indicate the acceptability limits (AL) of ±0.5 $\log_{10}$ CFU/g. Two samples were analyzed at each contamination level (low, intermediate, and high), with five replicate determinations performed per sample. The categories and selected product types included in the study are listed in Table 1 of the manuscript.

According to **ISO 16140-2:2014**, the Probability for the Tolerance Interval (**Proba TI**) must be **80%** and the central value must be the **median.**

**Proba TI (beta):**	80%
**Central Value:**	Median

(Food) Category	1
(Food) Type	2

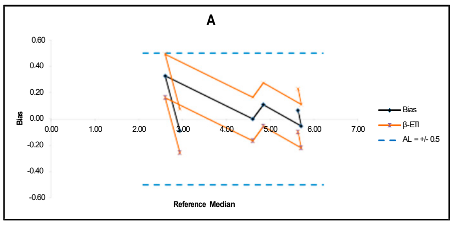

Sample Name	Reference central value	Bias	Lower β-ETI	Upper β-ETI	β-ETI compared to AL=±0.5 Acceptable	β-ETI compared to final AL Acceptable
	2.93	-0.089	-0.254	0.075	YES	YES
	2.60	0.327	0.163	0.492	YES	YES
	4.60	0.000	-0.165	0.165	YES	YES
	4.85	0.109	-0.055	0.274	YES	YES
	5.71	-0.054	-0.219	0.110	YES	YES
	5.63	0.066	-0.099	0.230	YES	YES

	Reference method	Alternative method	SD repeatability of reference method <= 0.125	Final AL
SD Repeatability	0.112	0.114	YES	+/- 0.500

(Food) Category	2
(Food) Type	1

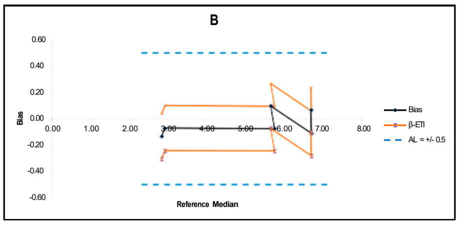

Sample Name	Reference Central value	Bias	Lower β-ETI	Upper β-ETI	β-ETI compared to AL=±0.5 Acceptable	β-ETI compared to final AL Acceptable
	2.81	-0.134	-0.304	0.036	YES	YES
	2.89	-0.073	-0.243	0.098	YES	YES
	5.70	-0.076	-0.246	0.095	YES	YES
	5.60	0.097	-0.073	0.267	YES	YES
	6.64	-0.112	-0.282	0.058	YES	YES
	6.63	0.066	-0.105	0.236	YES	YES

	Reference method	Alternative method	SD repeatability of reference method <= 0.125	Final AL
SD Repeatability	0.102	0.118	YES	+/- 0.500

(Food) Category	3
(Food) Type	2

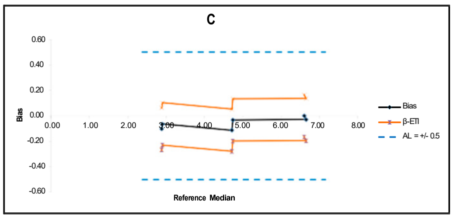

Sample Name	Reference Central value	Bias	Lower β-ETI	Upper β-ETI	β-ETI compared to AL=±0.5 Acceptable	β-ETI compared to final AL Acceptable
	2.88	-0.103	-0.269	0.063	YES	YES
	2.90	-0.064	-0.230	0.102	YES	YES
	4.72	-0.114	-0.280	0.052	YES	YES
	4.74	-0.033	-0.199	0.133	YES	YES
	6.65	-0.030	-0.196	0.136	YES	YES
	6.60	0.000	-0.166	0.166	YES	YES

	Reference method	Alternative method	SD repeatability of reference method <= 0.125	Final AL
SD Repeatability	0.113	0.115	YES	+/- 0.500

(Food) Category	4
(Food) Type	2

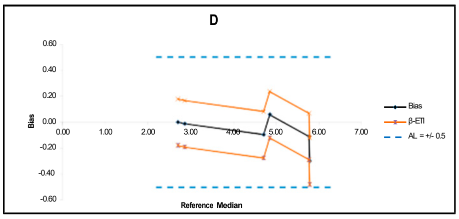

Sample Name	Reference Central value	Bias	Lower β-ETI	Upper β-ETI	β-ETI compared to AL=±0.5 Acceptable	β-ETI compared to final AL Acceptable
	2.70	0.000	-0.179	0.179	YES	YES
	2.86	-0.012	-0.191	0.167	YES	YES
	4.70	-0.097	-0.276	0.082	YES	YES
	4.85	0.058	-0.121	0.237	YES	YES
	5.76	-0.110	-0.289	0.069	YES	YES
	5.78	-0.301	-0.480	-0.122	YES	YES

	Reference method	Alternative method	SD repeatability of reference method <= 0.125	Final AL
SD Repeatability	0.122	0.124	YES	+/- 0.500

(Food) Category	5
(Food) Type	3

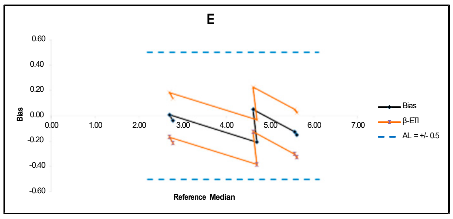

Sample Name	Reference Central value	Bias	Lower β-ETI	Upper β-ETI	β-ETI compared to AL=±0.5 Acceptable	β-ETI compared to final AL Acceptable
	2.78	-0.038	-0.214	0.138	YES	YES
	2.70	0.009	-0.168	0.185	YES	YES
	4.70	-0.208	-0.384	-0.031	YES	YES
	4.61	0.050	-0.126	0.226	YES	YES
	5.56	-0.125	-0.301	0.051	YES	YES
	5.61	-0.150	-0.327	0.026	YES	YES

	Reference method	Alternative method	SD repeatability of reference method <= 0.125	Final AL
SD Repeatability	0.118	0.122	YES	+/- 0.500

(Food) Category	6
(Food) Type	1

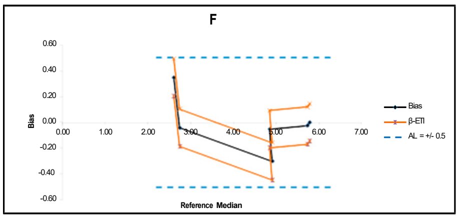

Sample Name	Reference Central value	Bias	Lower β-ETI	Upper β-ETI	β-ETI compared to AL=±0.5 Acceptable	β-ETI compared to final AL Acceptable
	2.60	0.347	0.203	0.492	YES	YES
	2.74	-0.041	-0.186	0.103	YES	YES
	4.90	-0.301	-0.445	-0.157	YES	YES
	4.85	-0.053	-0.197	0.092	YES	YES
	5.72	-0.025	-0.170	0.119	YES	YES
	5.78	0.000	-0.144	0.144	YES	YES

	Reference method	Alternative method	SD repeatability of reference method <= 0.125	Final AL
SD Repeatability	0.107	0.100	YES	+/- 0.500

(Feed) Category	7
(Feed) Type	2

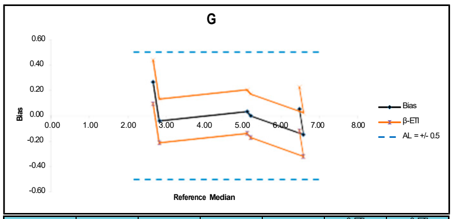

Sample Name	Reference Central value	Bias	Lower β-ETI	Upper β-ETI	β-ETI compared to AL=±0.5 Acceptable	β-ETI compared to final AL Acceptable
	2.66	0.267	0.095	0.438	YES	YES
	2.83	-0.041	-0.213	0.131	YES	YES
	5.11	0.032	-0.140	0.204	YES	YES
	5.20	0.000	-0.172	0.172	YES	YES
	6.58	-0.148	-0.320	0.023	YES	YES
	6.48	0.054	-0.117	0.226	YES	YES

	Reference method	Alternative method	SD repeatability of reference method <= 0.125	Final AL
SD Repeatability	0.118	0.119	YES	+/- 0.500

Figure S6. Bias and β-ETI limits, compliance with predefined acceptability criteria, and graphical presentation of the accuracy profile (AP) study results for **moulds** enumeration using the **DSBM-III** medium variation. The panels in Figure S6 show the results for the following food and feed categories and types: (A) Category 1, Type 2, (B) Category 2, Type 1, (C) Category 3, Type 2, (D) Category 4, Type 2, (E) Category 5, Type 3, (F) Category 6, Type 1, (G) Category 7, Type 2. Bias is expressed as $\log_{10}$ CFU/g. Black lines indicate bias, orange lines indicate the 80% β-expectation tolerance intervals (β-ETI), and blue dashed lines indicate the acceptability limits (AL) of ±0.5 $\log_{10}$ CFU/g. Two samples were analyzed at each contamination level (low, intermediate, and high), with five replicate determinations performed per sample. The categories and selected product types included in the study are listed in Table 1 of the manuscript.

Yeasts	cfu/g Reference method (R)	cfu/g Alternative method (A)	Moulds	cfu/g Reference method (R)	cfu/g Alternative method (A)	Yeasts and Moulds		cfu/g Reference method (R)	cfu/g Alternative method (A)	log10 cfu/g Reference method (R)	log10 cfu/g Alternative method (A)
Category 1	100	100	Category 1	200	100	Category 1	Type 1: Hard cheese	300	200	2.47712125	2.30103
	600	600		100	200			700	800	2.84509804	2.90308999
	17000000	18000000		100	100			17000100	18000100	7.23045148	7.25527492
	14000000	13000000		200	400			14000200	13000400	7.14613424	7.11395672
	32000000	30000000		200	300			32000200	30000300	7.50515269	7.4771256
	200000	280000		1500000	1670000		Type 2: Fruit fillings (artificially contaminated)	1700000	1950000	6.23044892	6.29003461
	200000	200000		1500000	1450000			1700000	1650000	6.23044892	6.21748394
	360000	300000		3500000	2000000			3860000	2300000	6.5865873	6.36172784
	16000	20000		140000	110000			156000	130000	5.1931246	5.11394335
	56000	53000		300000	240000			356000	293000	5.55145	5.46686762
	4700000	3700000		1500	1100		Type 3: Cured meat products	4701500	3701100	6.67223644	6.56833082
	58000	62000		1500	1900			59500	63900	4.77451697	4.80550086
	35000	37000		1300	600			36300	37600	4.55990663	4.57518784
	9200000	8100000		1800	1100			9201800	8101100	6.96387279	6.90854399
	17000	23000		1100	700			18100	23700	4.25767857	4.37474835
										2	2
										8	8
Category 2	6300000	6900000	Category 2	6000000	6400000	Category 2	Type 1: Fermented sausages	12300000	13300000	7.08990511	7.12385164
	800000	700000		780000	750000			1580000	1450000	6.19865709	6.161368
	400000	410000		470000	400000			870000	810000	5.93951925	5.90848502
	200	300		300	600			500	900	2.69897	2.95424251
	100	200		400	100			500	300	2.69897	2.47712125
	200	600		200	100		Type 2: Meat products (bacon, pancetta, dry-cured pork	400	700	2.60205999	2.84509804
	100	300		100	300			200	600	2.30103	2.77815125
	400	200		100	200			500	400	2.69897	2.60205999
	200	500		200	300			400	800	2.60205999	2.90308999
	200	300		200	100			400	400	2.60205999	2.60205999
	10000	9000		140000	40000		Type 3: meat products (artificially contaminated)	150000	49000	5.17609126	4.69019608
	1000	4000		43000	46000			44000	50000	4.64345268	4.69897
	6000	2000		21000	23000			27000	25000	4.43136376	4.39794001
	4000	1000		9000	11000			13000	12000	4.11394335	4.07918125
	200	100		14000	8000			14200	8100	4.15228834	3.90848502
										2	2
										8	8
Category 3	2720000	2480000	Category 3	2720000	2480000	Category 3	Type 1: Fermented sausages	5440000	4960000	6.7355989	6.69548168
	100	100		100	100			200	200	2.30103	2.30103
	33000	27000		33000	27000			66000	54000	4.81954394	4.73239376
	100000	200000		100000	200000			200000	400000	5.30103	5.60205999
	90000	110000		90000	110000			180000	220000	5.25527251	5.34242268
	300	400		300	400		Type 2: Meat products (bacon, dry-cured pork neck)	600	800	2.77815125	2.90308999
	200	300		200	300			400	600	2.60205999	2.77815125
	650000	800000		650000	800000			1300000	1600000	6.11394335	6.20411998
	200	400		200	400			400	800	2.60205999	2.90308999
	200	100		200	100			400	200	2.60205999	2.30103
	100000	190000		2700000	2900000		Type 3: Marmalade (artificially contaminated)	2800000	3090000	6.44715803	6.48995848
	130000	80000		1800000	1640000			1930000	1720000	6.28555731	6.23552845
	270000	180000		2600000	2680000			2870000	2860000	6.4578819	6.45636603
	200000	180000		3000000	2790000			3200000	2970000	6.50514998	6.47275645
	140000	200000		2600000	2430000			2740000	2630000	6.43775056	6.41995575
										2	2
										8	8
Category 4	7200	8500	Category 4	7200	8500	Category 4	Type 1: Meat products (bacon, homemade sausage,	14400	17000	4.15836249	4.23044892
	100	200		100	300			200	500	2.30103	2.69897
	200	100		400	100			600	200	2.77815125	2.30103
	100	300		100	500			200	800	2.30103	2.90308999
	100	100		200	200			300	300	2.47712125	2.47712125
	100000	150000		2700000	2700000		Type 2: Marmalade (artificially contaminated)	2800000	2850000	6.44715803	6.45484486
	130000	210000		1800000	2600000			1930000	2810000	6.28555731	6.44870632
	270000	60000		2600000	2500000			2870000	2560000	6.4578819	6.40823997
	200000	180000		3000000	2800000			3200000	2980000	6.50514998	6.47421626
	140000	130000		2600000	2100000			2740000	2230000	6.43775056	6.34830486
	500	100		1500	1000		Type 3: Confectionery products (artificially contaminated)	2000	1100	3.30103	3.04139269
	400	200		900	1700			1300	1900	3.11394335	3.2787536
	600	200		1400	1400			2000	1600	3.30103	3.20411998
	300	300		2100	1100			2400	1400	3.38021124	3.14612804
	600	500		2200	2200			2800	2700	3.44715803	3.43136376
										2	2
										8	8
Category 5	13000	30000	Category 5	118000	115000	Category 5	Type 1: Beans (artificially contaminated)	131000	145000	5.1172713	5.161368
	12000	13000		64000	61000			76000	74000	4.88081359	4.86923172
	4000	8000		21000	35000			25000	43000	4.39794001	4.63346846
	3000	5000		17000	13000			20000	18000	4.30103	4.25527251
	2700	4000		18000	19000			20700	23000	4.31597035	4.36172784
	10000	30000		60000	90000		Type 2 Dried/candied fruit (artificially contaminated)	70000	120000	4.84509804	5.07918125
	9000	10000		47000	54000			56000	64000	4.74818803	4.80617997
	4000	4000		34000	16000			38000	20000	4.5797836	4.30103
	2000	1000		31000	16000			33000	17000	4.51851394	4.23044892
	4000	2000		14000	20000			18000	22000	4.25527251	4.34242268
	33000	30000		90000	60000		Type 3: Flours (artificially contaminated)	123000	90000	5.08990511	4.95424251
	9000	300		79000	32000			88000	32300	4.94448267	4.50920252
	7000	4000		18000	17000			25000	21000	4.39794001	4.32221929
	1600	2000		8000	4000			9600	6000	3.98227123	3.77815125
	1500	1600		7000	5000			8500	6600	3.92941893	3.81954394
										2	2
										8	8
Category 6	130000	160000	Category 6	3000000	2400000	Category 6	Type 1: Candies (artificially contaminated)	3130000	2560000	6.49554434	6.40823997
	100000	50000		2100000	1500000			2200000	1550000	6.34242268	6.1903317
	40000	40000		1000000	1100000			1040000	1140000	6.01703334	6.05690485
	25000	21000		430000	510000			455000	531000	5.6580114	5.72509452
	12000	17000		263000	290000			275000	307000	5.43933269	5.48713838
	300	200		600	700		Type 2: Dried/candied fruit (artificially contaminated)	900	900	2.95424251	2.95424251
	400	400		1100	600			1500	1000	3.17609126	3
	100	100		800	900			900	1000	2.95424251	3
	100	100		1300	900			1400	1000	3.14612804	3
	200	100		1000	800			1200	900	3.07918125	2.95424251
	200000	100000		4700000	4000000		Type 3: Flours (artificially contaminated)	4900000	4100000	6.69019608	6.61278386
	50000	50000		1600000	1200000			1650000	1250000	6.21748394	6.09691001
	60000	20000		1300000	950000			1360000	970000	6.13353891	5.98677173
	14000	11000		280000	290000			294000	301000	5.46834733	5.4785665
	6000	5000		95000	65000			101000	70000	5.00432137	4.84509804
										2	2
										8	8
Category 7	300	400	Category 7	1100	900	Category 7	Type 1: Pet food sausages (artificially contaminated)	1400	1300	3.14612804	3.11394335
	400	200		1900	1500			2300	1700	3.36172784	3.23044892
	200	200		1700	1300			1900	1500	3.2787536	3.17609126
	200	1100		2000	1100			2200	2200	3.34242268	3.34242268
	100	200		1500	1000			1600	1200	3.20411998	3.07918125
	2400	2400		2400	2400		Type 2: Starter cultures/mixes (artificially contaminated)	4800	4800	3.68124124	3.68124124
	100	300		100	400			200	700	2.30103	2.84509804
	5800000	4500000		5800000	4500000			11600000	9000000	7.06445799	6.95424251
	600000	300000		600000	300000			1200000	600000	6.07918125	5.77815125
	210000	80000		210000	80000			420000	160000	5.62324929	5.20411998
	40000	30000		240000	180000		Type 3: Feed grains (artificially contaminated)	280000	210000	5.44715803	5.32221929
	22000	12000		51000	52000			73000	64000	4.86332286	4.80617997
	8000	5000		16000	21000			24000	26000	4.38021124	4.41497335
	6000	3300		19000	18000			25000	21300	4.39794001	4.3283796
	700	1400		17000	17000			17700	18400	4.24797327	4.26481782
										2	2
										8	8

T	n	1+1/n	SQRT (1+1/n)	Category	log10 cfu/g Reference method (R)	log10 cfu/g Alternative method (A)	Mean (Average)	Difference (D=A-R)	Aver. Dall	Zero line	Upper 95% confidence limit of agreement	Lower 95% confidence limit of agreement
1.983	105	1.009524	1.004751	Category 1 0,91 ≤ aw ≤ 0,95	2.47712125	2.30103	2.38907563	-0.176091259	-0.02191	0	0.347	-0.391
					2.84509804	2.90308999	2.87409401	0.057991947	-0.02191	0	0.347	-0.391
					7.23045148	7.25527492	7.2428632	0.024823442	-0.02191	0	0.347	-0.391
					7.14613424	7.11395672	7.13004548	-0.032177525	-0.02191	0	0.347	-0.391
					7.50515269	7.4771256	7.49113915	-0.028027095	-0.02191	0	0.347	-0.391
					6.23044892	6.29003461	6.26024177	0.05958569	-0.02191	0	0.347	-0.391
					6.23044892	6.21748394	6.22396643	-0.012964977	-0.02191	0	0.347	-0.391
					6.5865873	6.36172784	6.47415757	-0.224859469	-0.02191	0	0.347	-0.391
					5.1931246	5.11394335	5.15353398	-0.079181246	-0.02191	0	0.347	-0.391
					5.55145	5.46686762	5.50915881	-0.084582378	-0.02191	0	0.347	-0.391
					6.67223644	6.56833082	6.62028363	-0.103905621	-0.02191	0	0.347	-0.391
					4.77451697	4.80550086	4.79000891	0.030983892	-0.02191	0	0.347	-0.391
					4.55990663	4.57518784	4.56754723	0.01528122	-0.02191	0	0.347	-0.391
					6.96387279	6.90854399	6.93620839	-0.055328797	-0.02191	0	0.347	-0.391
					4.25767857	4.37474835	4.31621346	0.117069771	-0.02191	0	0.347	-0.391
				Category 2 0,87 < aw ≤ 0,91	7.08990511	7.12385164	7.10687838	0.03394653	-0.02191	0	0.347	-0.391
					6.19865709	6.161368	6.18001254	-0.037289085	-0.02191	0	0.347	-0.391
					5.93951925	5.90848502	5.92400214	-0.031034234	-0.02191	0	0.347	-0.391
					2.69897	2.95424251	2.82660626	0.255272505	-0.02191	0	0.347	-0.391
					2.69897	2.47712125	2.58804563	-0.22184875	-0.02191	0	0.347	-0.391
					2.60205999	2.84509804	2.72357902	0.243038049	-0.02191	0	0.347	-0.391
					2.30103	2.77815125	2.53959062	0.477121255	-0.02191	0	0.347	-0.391
					2.69897	2.60205999	2.650515	-0.096910013	-0.02191	0	0.347	-0.391
					2.60205999	2.90308999	2.75257499	0.301029996	-0.02191	0	0.347	-0.391
					2.60205999	2.60205999	2.60205999	0	-0.02191	0	0.347	-0.391
					5.17609126	4.69019608	4.93314367	-0.485895179	-0.02191	0	0.347	-0.391
					4.64345268	4.69897	4.67121134	0.055517328	-0.02191	0	0.347	-0.391
					4.43136376	4.39794001	4.41465189	-0.033423755	-0.02191	0	0.347	-0.391
					4.11394335	4.07918125	4.0965623	-0.034762106	-0.02191	0	0.347	-0.391
					4.15228834	3.90848502	4.03038668	-0.243803326	-0.02191	0	0.347	-0.391
				Category 3 0,80 < aw ≤ 0,87	6.7355989	6.69548168	6.71554029	-0.040117223	-0.02191	0	0.347	-0.391
					2.30103	2.30103	2.30103	0	-0.02191	0	0.347	-0.391
					4.81954394	4.73239376	4.77596885	-0.087150176	-0.02191	0	0.347	-0.391
					5.30103	5.60205999	5.45154499	0.301029996	-0.02191	0	0.347	-0.391
					5.25527251	5.34242268	5.29884759	0.087150176	-0.02191	0	0.347	-0.391
					2.77815125	2.90308999	2.84062062	0.124938737	-0.02191	0	0.347	-0.391
					2.60205999	2.77815125	2.69010562	0.176091259	-0.02191	0	0.347	-0.391
					6.11394335	6.20411998	6.15903167	0.09017663	-0.02191	0	0.347	-0.391
					2.60205999	2.90308999	2.75257499	0.301029996	-0.02191	0	0.347	-0.391
					2.60205999	2.30103	2.45154499	-0.301029996	-0.02191	0	0.347	-0.391
					6.44715803	6.48995848	6.46855826	0.042800448	-0.02191	0	0.347	-0.391
					6.28555731	6.23552845	6.26054288	-0.050028862	-0.02191	0	0.347	-0.391
					6.4578819	6.45636603	6.45712396	-0.001515864	-0.02191	0	0.347	-0.391
					6.50514998	6.47275645	6.48895321	-0.032393529	-0.02191	0	0.347	-0.391
					6.43775056	6.41995575	6.42885316	-0.017794814	-0.02191	0	0.347	-0.391
				Category 4 0,75 < aw ≤ 0,80	4.15836249	4.23044892	4.19440571	0.072086429	-0.02191	0	0.347	-0.391
					2.30103	2.69897	2.5	0.397940009	-0.02191	0	0.347	-0.391
					2.77815125	2.30103	2.53959062	-0.477121255	-0.02191	0	0.347	-0.391
					2.30103	2.90308999	2.60205999	0.602059991	-0.02191	0	0.347	-0.391
					2.47712125	2.47712125	2.47712125	0	-0.02191	0	0.347	-0.391
					6.44715803	6.45484486	6.45100145	0.007686829	-0.02191	0	0.347	-0.391
					6.28555731	6.44870632	6.36713181	0.163149011	-0.02191	0	0.347	-0.391
					6.4578819	6.40823997	6.43306093	-0.049641931	-0.02191	0	0.347	-0.391
					6.50514998	6.47421626	6.48968312	-0.030933714	-0.02191	0	0.347	-0.391
					6.43775056	6.34830486	6.39302771	-0.0894457	-0.02191	0	0.347	-0.391
					3.30103	3.04139269	3.17121134	-0.259637311	-0.02191	0	0.347	-0.391
					3.11394335	3.2787536	3.19634848	0.164810249	-0.02191	0	0.347	-0.391
					3.30103	3.20411998	3.25257499	-0.096910013	-0.02191	0	0.347	-0.391
					3.38021124	3.14612804	3.26316964	-0.234083206	-0.02191	0	0.347	-0.391
					3.44715803	3.43136376	3.4392609	-0.015794267	-0.02191	0	0.347	-0.391
				Category 5 0,65 < aw ≤ 0,75	5.1172713	5.161368	5.13931965	0.044096707	-0.02191	0	0.347	-0.391
					4.88081359	4.86923172	4.87502266	-0.011581873	-0.02191	0	0.347	-0.391
					4.39794001	4.63346846	4.51570423	0.235528447	-0.02191	0	0.347	-0.391
					4.30103	4.25527251	4.27815125	-0.045757491	-0.02191	0	0.347	-0.391
					4.31597035	4.36172784	4.33884909	0.045757491	-0.02191	0	0.347	-0.391
					4.84509804	5.07918125	4.96213964	0.234083206	-0.02191	0	0.347	-0.391
					4.74818803	4.80617997	4.777184	0.057991947	-0.02191	0	0.347	-0.391
					4.5797836	4.30103	4.4404068	-0.278753601	-0.02191	0	0.347	-0.391
					4.51851394	4.23044892	4.37448143	-0.288065018	-0.02191	0	0.347	-0.391
					4.25527251	4.34242268	4.29884759	0.087150176	-0.02191	0	0.347	-0.391
					5.08990511	4.95424251	5.02207381	-0.135662602	-0.02191	0	0.347	-0.391
					4.94448267	4.50920252	4.7268426	-0.43528015	-0.02191	0	0.347	-0.391
					4.39794001	4.32221929	4.36007965	-0.075720714	-0.02191	0	0.347	-0.391
					3.98227123	3.77815125	3.88021124	-0.204119983	-0.02191	0	0.347	-0.391
					3.92941893	3.81954394	3.87448143	-0.10987499	-0.02191	0	0.347	-0.391
				Category 6 0,60 < aw ≤ 0,65	6.49554434	6.40823997	6.45189215	-0.087304372	-0.02191	0	0.347	-0.391
					6.34242268	6.1903317	6.26637719	-0.152090983	-0.02191	0	0.347	-0.391
					6.01703334	6.05690485	6.0369691	0.039871512	-0.02191	0	0.347	-0.391
					5.6580114	5.72509452	5.69155296	0.067083124	-0.02191	0	0.347	-0.391
					5.43933269	5.48713838	5.46323553	0.047805682	-0.02191	0	0.347	-0.391
					2.95424251	2.95424251	2.95424251	0	-0.02191	0	0.347	-0.391
					3.17609126	3	3.08804563	-0.176091259	-0.02191	0	0.347	-0.391
					2.95424251	3	2.97712125	0.045757491	-0.02191	0	0.347	-0.391
					3.14612804	3	3.07306402	-0.146128036	-0.02191	0	0.347	-0.391
					3.07918125	2.95424251	3.01671188	-0.124938737	-0.02191	0	0.347	-0.391
					6.69019608	6.61278386	6.65148997	-0.077412223	-0.02191	0	0.347	-0.391
					6.21748394	6.09691001	6.15719698	-0.120573931	-0.02191	0	0.347	-0.391
					6.13353891	5.98677173	6.06015532	-0.146767174	-0.02191	0	0.347	-0.391
					5.46834733	5.4785665	5.47345691	0.010219165	-0.02191	0	0.347	-0.391
					5.00432137	4.84509804	4.92470971	-0.159223334	-0.02191	0	0.347	-0.391
				Category 7 0,60 < aw ≤ 0,65	3.14612804	3.11394335	3.13003569	-0.032184683	-0.02191	0	0.347	-0.391
					3.36172784	3.23044892	3.29608838	-0.131278915	-0.02191	0	0.347	-0.391
					3.2787536	3.17609126	3.22742243	-0.102662342	-0.02191	0	0.347	-0.391
					3.34242268	3.34242268	3.34242268	0	-0.02191	0	0.347	-0.391
					3.20411998	3.07918125	3.14165061	-0.124938737	-0.02191	0	0.347	-0.391
					3.68124124	3.68124124	3.68124124	0	-0.02191	0	0.347	-0.391
					2.30103	2.84509804	2.57306402	0.544068044	-0.02191	0	0.347	-0.391
					7.06445799	6.95424251	7.00935025	-0.11021548	-0.02191	0	0.347	-0.391
					6.07918125	5.77815125	5.92866625	-0.301029996	-0.02191	0	0.347	-0.391
					5.62324929	5.20411998	5.41368464	-0.419129308	-0.02191	0	0.347	-0.391
					5.44715803	5.32221929	5.38468866	-0.124938737	-0.02191	0	0.347	-0.391
					4.86332286	4.80617997	4.83475142	-0.057142886	-0.02191	0	0.347	-0.391
					4.38021124	4.41497335	4.39759229	0.034762106	-0.02191	0	0.347	-0.391
					4.39794001	4.3283796	4.36315981	-0.069560405	-0.02191	0	0.347	-0.391
					4.24797327	4.26481782	4.25639554	0.016844557	-0.02191	0	0.347	-0.391
				Aver. Dall				-0.021909329				
				SDall				0.185135739				

Table S6. Raw data and statistical analysis of the agreement between the reference DG18 medium and the alternative **DSBM-I** medium variation for the enumeration of yeasts and moulds across selected food and feed categories and types.

Yeasts	cfu/g Reference method (R)	cfu/g Alternative method (A)	Moulds	cfu/g Reference method (R)	cfu/g Alternative method (A)	Yeasts and Moulds		cfu/g Reference method (R)	cfu/g Alternative method (A)	log10 cfu/g Reference method (R)	log10 cfu/g Alternative method (A)	T
Category 1	100	200	Category 1	200	300	Category 1	Type 1: Hard cheese	300	500	2.4771212	2.69897	1.983
	600	700		100	400			700	1100	2.8450980	3.0413926	
	17000000	17500000		100	200			17000100	17500200	7.2304514	7.2430450	
	14000000	16000000		400	100			14000400	16000100	7.1461404	7.204122	
	32000000	33000000		200	500			32000200	33000500	7.5051528	7.5440742	
	200000	100000		1500000	1600000		Type 2: Fruit fillings (artificially contaminated)	1700000	1700000	6.2304489	6.2304489	
	200000	200000		1500000	1420000			1700000	1620000	6.2304489	6.2095150	
	360000	300000		3300000	3000000			3660000	3300000	6.563587	6.5185139	
	16000	22000		140000	150000			156000	172000	5.193124	5.2355284	
	36000	38000		300000	280000			336000	318000	5.5314	5.5024271	
	4700000	4100000		1500	2200		Type 3: Cured meat products	4701500	4102200	6.6722364	6.6130168	
	58000	60000		1500	2100			59500	62100	4.7745169	4.793091	
	35000	39000		1300	1100			36300	40100	4.5599066	4.6031443	
	9200000	8800000		1800	1700			9201800	8801700	6.9638727	6.9445663	
	17000	21000		1100	1100			18100	22100	4.2576785	4.3443922	
[illegible]	[illegible]	[illegible]	[illegible]	[illegible]	[illegible]	[illegible]	[illegible]	[illegible]	[illegible]	[illegible]	[illegible]	[illegible]

Table S7. Raw data and statistical analysis of the agreement between the reference DG18 medium and the alternative **DSBM-II** medium variation for the enumeration of yeasts and moulds across selected food and feed categories and types.

Yeasts	cfu/g		Moulds	cfu/g		Yeasts and Moulds		cfu/g		log10 cfu/g	
	Reference method (R)	Alternative method (A)		Reference method (R)	Alternative method (A)			Reference method (R)	Alternative method (A)	Reference method (R)	Alternative method (A)
Category 1	100	300	Category 1	100	300	Category 1	Type 1: Hard cheese	200	600	2.30103	2.77815125
	600	900		200	200			800	1100	2.90308999	3.04139269
	17000000	14000000		400	200			17000400	14000200	7.23045914	7.14613424
	14000000	16000000		100	400			14000100	16000400	7.14613114	7.20413084
	32000000	31000000		300	100			32000300	31000100	7.50515405	7.49136309
	200000	200000		1500000	1480000		Type 2: Fruit fillings (artificially contaminated)	1700000	1680000	6.23044892	6.22530928
	200000	250000		1500000	1550000			1700000	1800000	6.23044892	6.25527251
	360000	300000		3500000	3300000			3860000	3600000	6.5865873	6.5563025
	16000	22000		140000	100000			156000	122000	5.1931246	5.08635983
	56000	50000		300000	380000			356000	430000	5.55145	5.63346846
	4700000	5400000		1500	900		Type 3: Cured meat products	4701500	5400900	6.67223644	6.73246614
	5800000	6100000		1500	1100			5801500	6101100	6.7635403	6.78540814
	250000	290000		1300	300			251300	290300	5.40019249	5.46284704
	9200000	8900000		1800	1800			9201800	8901800	6.96387279	6.94947783
	17000	23000		1100	1000			18100	24000	4.25767857	4.38021124
										2	2
										8	8
Category 2	6300000	6700000	Category 2	5900000	6700000	Category 2	Type 1: Fermented sausages	12200000	13400000	7.08635983	7.1271048
	800000	740000		850000	820000			1650000	1560000	6.21748394	6.1931246
	400000	450000		420000	460000			820000	910000	5.91381385	5.95904139
	200	100		200	200			400	300	2.60205999	2.47712125
	100	300		400	600			500	900	2.69897	2.95424251
	200	100		100	300		Type 2: Meat products (bacon, pancetta, dry-cured pork loin,	300	400	2.47712125	2.60205999
	100	400		300	200			400	600	2.60205999	2.77815125
	100	300		400	200			500	500	2.69897	2.69897
	200	200		100	200			300	400	2.47712125	2.60205999
	200	100		200	100			400	200	2.60205999	2.30103
	10000	8000		140000	139000		Type 3: meat products (artificially contaminated)	150000	147000	5.17609126	5.16731733
	1000	3000		43000	47000			44000	50000	4.64345268	4.69897
	6000	2000		21000	31000			27000	33000	4.43136376	4.51851394
	4000	3000		9000	23000			13000	26000	4.11394335	4.41497335
	200	300		14000	21000			14200	21300	4.15228834	4.3283796
										2	2
										8	8
Category 3	2720000	2650000	Category 3	2500000	2530000	Category 3	Type 1: Fermented sausages	5220000	5180000	6.7176705	6.71432976
	100	400		200	400			300	800	2.47712125	2.90308999
	33000	35000		30000	28000			63000	63000	4.79934055	4.79934055
	100000	120000		150000	130000			250000	250000	5.39794001	5.39794001
	90000	100000		100000	92000			190000	192000	5.2787536	5.28330123
	300	500		200	100		Type 2: Meat products (bacon, dry-cured pork neck)	500	600	2.69897	2.77815125
	200	200		300	100			500	300	2.69897	2.47712125
	650000	710000		710000	750000			1360000	1460000	6.13353891	6.16435286
	200	100		100	300			300	400	2.47712125	2.60205999
	200	400		300	500			500	900	2.69897	2.95424251
	100000	180000		2700000	2830000		Type 3: Marmalade (artificially contaminated)	2800000	3010000	6.44715803	6.4785665
	130000	170000		1800000	2600000			1930000	2770000	6.28555731	6.44247977
	270000	120000		2600000	2170000			2870000	2290000	6.4578819	6.35983548
	200000	170000		3000000	2470000			3200000	2640000	6.50514998	6.42160393
	140000	150000		2600000	2320000			2740000	2470000	6.43775056	6.39269695
										2	2
										8	8
Category 4	7200	7000	Category 4	7600	8000	Category 4	Type 1: Meat products (bacon, homemade sausage, kulen)	14800	15000	4.17026172	4.17609126
	100	300		100	400			200	700	2.30103	2.84509804
	200	200		300	300			500	500	2.69897	2.69897
	100	200		200	300			300	500	2.47712125	2.69897
	100	400		300	500			400	900	2.60205999	2.95424251
	100000	140000		2700000	2700000		Type 2: Marmalade (artificially contaminated)	2800000	2840000	6.44715803	6.45331834
	130000	190000		1800000	2200000			1930000	2390000	6.28555731	6.3783979
	270000	180000		2600000	1900000			2870000	2080000	6.4578819	6.31806333
	200000	300000		3000000	2700000			3200000	3000000	6.50514998	6.47712125
	140000	120000		2600000	2100000			2740000	2220000	6.43775056	6.34635297
	500	300		1500	1700		Type 3: Confectionery products (artificially contaminated)	2000	2000	3.30103	3.30103
	400	700		900	1500			1300	2200	3.11394335	3.34242268
	600	100		1400	1600			2000	1700	3.30103	3.23044892
	300	100		2100	1300			2400	1400	3.38021124	3.14612804
	600	1000		2200	2200			2800	3200	3.44715803	3.50514998
										2	2
										8	8
Category 5	13000	13000	Category 5	118000	130000	Category 5	Type 1: Beans (artificially contaminated)	131000	143000	5.1172713	5.15533604
	12000	14000		64000	56000			76000	70000	4.88081359	4.84509804
	4000	7000		21000	24000			25000	31000	4.39794001	4.49136169
	3000	4000		17000	12000			20000	16000	4.30103	4.20411998
	2700	2200		18000	17700			20700	19900	4.31597035	4.29885308
	10000	12000		60000	130000		Type 2: Dried/candied fruit (artificially contaminated))	70000	142000	4.84509804	5.15228834
	9000	20000		47000	45000			56000	65000	4.74818803	4.81291336
	4000	8000		34000	26000			38000	34000	4.5797836	4.53147892
	2000	4000		31000	27000			33000	31000	4.51851394	4.49136169
	4000	1000		14000	10000			18000	11000	4.25527251	4.04139269
	33000	15000		90000	130000		Type 3: Flours (artificially contaminated)	123000	145000	5.08990511	5.161368
	9000	200		79000	11000			88000	11200	4.94448267	4.04921802
	7000	2000		18000	9000			25000	11000	4.39794001	4.04139269
	1600	2900		8000	5000			9600	7900	3.98227123	3.89762709
	1500	1900		7000	3000			8500	4900	3.92941893	3.69019608
										2	2
										8	8
Category 6	130000	11000	Category 6	3000000	1800000	Category 6	Type 1: Candies (artificially contaminated)	3130000	1811000	6.49554434	6.25791845
	100000	70000		2100000	1400000			2200000	1470000	6.34242268	6.16731733
	40000	20000		1000000	600000			1040000	620000	6.01703334	5.79239169
	25000	24000		430000	390000			455000	414000	5.6580114	5.61700034
	12000	7000		263000	240000			275000	247000	5.43933269	5.39269695
	300	200		600	500		Type 2: Dried/candied fruit (artificially contaminated)	900	700	2.95424251	2.84509804
	400	200		1100	1200			1500	1400	3.17609126	3.14612804
	100	300		800	1700			900	2000	2.95424251	3.30103
	100	200		1300	300			1400	500	3.14612804	2.69897
	200	200		1000	500			1200	700	3.07918125	2.84509804
	200000	200000		4700000	3000000		Type 3: Flours (artificially contaminated)	4900000	3200000	6.69019608	6.50514998
	50000	90000		1600000	900000			1650000	990000	6.21748394	5.99563519
	60000	30000		1300000	1500000			1360000	1530000	6.13353891	6.18469143
	14000	10000		280000	260000			294000	270000	5.46834733	5.43136376
	6000	6000		95000	60000			101000	66000	5.00432137	4.81954394
										2	2
										8	8
Category 7	300	100	Category 7	1100	1000	Category 7	Type 1: Pet food sausages (artificially contaminated)	1400	1100	3.14612804	3.04139269
	400	300		1900	1400			2300	1700	3.36172784	3.23044892
	200	100		1700	1600			1900	1700	3.2787536	3.23044892
	200	100		2000	1400			2200	1500	3.34242268	3.17609126
	100	200		1500	1000			1600	1200	3.20411998	3.07918125
	2400	2600		3000	2500		Type 2: Starter cultures/mixes (artificially contaminated)	5400	5100	3.73239376	3.70757018
	100	300		400	100			500	400	2.69897	2.60205999
	5800000	5200000		5100000	4900000			10900000	10100000	7.0374265	7.00432137
	600000	700000		550000	620000			1150000	1320000	6.06069784	6.12057393
	210000	200000		1500000	190000			1710000	390000	6.23299611	5.59106461
	40000	26000		240000	210000		Type 3: Feed grains (artificially contaminated)	280000	236000	5.44715803	5.372912
	22000	12000		51000	60000			73000	72000	4.86332286	4.8573325
	8000	7000		16000	30000			24000	37000	4.38021124	4.56820172
	6000	3000		19000	20000			25000	23000	4.39794001	4.36172784
	700	4000		17000	15000			17700	19000	4.24797327	4.2787536
										2	2
										8	8

T	n	1+1/n	SQRT (1+1/n)	Category	log10 cfu/g Reference method (R)	log10 cfu/g Alternative method (A)	Mean (Average)	Difference (D=A-R)	Aver. Dall	Zero line	Upper 95% confidence limit of agreement	Lower 95% confidence limit of agreement
1.983	105	1.009524	1.004751	Category 1 0,91 ≤ aw ≤ 0,95	2.30103	2.77815125	2.53959062	0.477121255	-0.0105	0	0.380	-0.402
					2.90309	3.041392685	2.97224134	0.138302698	-0.0105	0	0.380	-0.402
					7.2304591	7.14613424	7.18829669	-0.0843249	-0.0105	0	0.380	-0.402
					7.1461311	7.20413084	7.17513099	0.057999702	-0.0105	0	0.380	-0.402
					7.505154	7.491363095	7.49825857	-0.013790955	-0.0105	0	0.380	-0.402
					6.2304489	6.225309282	6.2278791	-0.00513964	-0.0105	0	0.380	-0.402
					6.2304489	6.255272505	6.24286071	0.024823584	-0.0105	0	0.380	-0.402
					6.5865873	6.556302501	6.5714449	-0.030284804	-0.0105	0	0.380	-0.402
					5.1931246	5.086359831	5.13974221	-0.106764768	-0.0105	0	0.380	-0.402
					5.55145	5.633468456	5.59245923	0.082018458	-0.0105	0	0.380	-0.402
					6.6722364	6.732466136	6.70235129	0.060229696	-0.0105	0	0.380	-0.402
					6.7635403	6.785408143	6.77447422	0.021867847	-0.0105	0	0.380	-0.402
					5.4001925	5.462847036	5.43151976	0.062654547	-0.0105	0	0.380	-0.402
					6.9638728	6.949477833	6.95667531	-0.014394957	-0.0105	0	0.380	-0.402
					4.2576786	4.380211242	4.31894491	0.122532667	-0.0105	0	0.380	-0.402
				Category 2 0,87 < aw ≤ 0,91	7.0863598	7.127104798	7.10673231	0.040744968	-0.0105	0	0.380	-0.402
					6.2174839	6.193124598	6.20530427	-0.024359346	-0.0105	0	0.380	-0.402
					5.9138139	5.959041392	5.93642762	0.04522754	-0.0105	0	0.380	-0.402
					2.60206	2.477121255	2.53959062	-0.124938737	-0.0105	0	0.380	-0.402
					2.69897	2.954242509	2.82660626	0.255272505	-0.0105	0	0.380	-0.402
					2.4771213	2.602059991	2.53959062	0.124938737	-0.0105	0	0.380	-0.402
					2.60206	2.77815125	2.69010562	0.176091259	-0.0105	0	0.380	-0.402
					2.69897	2.698970004	2.69897	0	-0.0105	0	0.380	-0.402
					2.4771213	2.602059991	2.53959062	0.124938737	-0.0105	0	0.380	-0.402
					2.60206	2.301029996	2.45154499	-0.301029996	-0.0105	0	0.380	-0.402
					5.1760913	5.167317335	5.1717043	-0.008773924	-0.0105	0	0.380	-0.402
					4.6434527	4.698970004	4.67121134	0.055517328	-0.0105	0	0.380	-0.402
					4.4313638	4.51851394	4.47493885	0.087150176	-0.0105	0	0.380	-0.402
					4.1139434	4.414973348	4.26445835	0.301029996	-0.0105	0	0.380	-0.402
					4.1522883	4.328379603	4.24033397	0.176091259	-0.0105	0	0.380	-0.402
				Category 3 0,80 < aw ≤ 0,87	6.7176705	6.71432976	6.71600013	-0.003340743	-0.0105	0	0.380	-0.402
					2.4771213	2.903089987	2.69010562	0.425968732	-0.0105	0	0.380	-0.402
					4.7993405	4.799340549	4.79934055	0	-0.0105	0	0.380	-0.402
					5.39794	5.397940009	5.39794001	0	-0.0105	0	0.380	-0.402
					5.2787536	5.283301229	5.28102741	0.004547628	-0.0105	0	0.380	-0.402
					2.69897	2.77815125	2.73856063	0.079181246	-0.0105	0	0.380	-0.402
					2.69897	2.477121255	2.58804563	-0.22184875	-0.0105	0	0.380	-0.402
					6.1335389	6.164352856	6.14894588	0.030813947	-0.0105	0	0.380	-0.402
					2.4771213	2.602059991	2.53959062	0.124938737	-0.0105	0	0.380	-0.402
					2.69897	2.954242509	2.82660626	0.255272505	-0.0105	0	0.380	-0.402
					6.447158	6.478566496	6.46286226	0.031408464	-0.0105	0	0.380	-0.402
					6.2855573	6.442479769	6.36401854	0.15692246	-0.0105	0	0.380	-0.402
					6.4578819	6.359835482	6.40885869	-0.098046414	-0.0105	0	0.380	-0.402
					6.50515	6.421603927	6.46337695	-0.083546051	-0.0105	0	0.380	-0.402
					6.4377506	6.392696953	6.41522376	-0.04505361	-0.0105	0	0.380	-0.402
				Category 4 0,75 < aw ≤ 0,80	4.1702617	4.176091259	4.17317649	0.005829544	-0.0105	0	0.380	-0.402
					2.30103	2.84509804	2.57306402	0.544068044	-0.0105	0	0.380	-0.402
					2.69897	2.698970004	2.69897	0	-0.0105	0	0.380	-0.402
					2.4771213	2.698970004	2.58804563	0.22184875	-0.0105	0	0.380	-0.402
					2.60206	2.954242509	2.77815125	0.352182518	-0.0105	0	0.380	-0.402
					6.447158	6.45331834	6.45023819	0.006160309	-0.0105	0	0.380	-0.402
					6.2855573	6.378397901	6.3319776	0.092840592	-0.0105	0	0.380	-0.402
					6.4578819	6.318063335	6.38797262	-0.139818562	-0.0105	0	0.380	-0.402
					6.50515	6.477121255	6.49113562	-0.028028724	-0.0105	0	0.380	-0.402
					6.4377506	6.346352974	6.39205177	-0.091397588	-0.0105	0	0.380	-0.402
					3.30103	3.301029996	3.30103	0	-0.0105	0	0.380	-0.402
					3.1139434	3.342422681	3.22818302	0.228479329	-0.0105	0	0.380	-0.402
					3.30103	3.230448921	3.26573946	-0.070581074	-0.0105	0	0.380	-0.402
					3.3802112	3.146128036	3.26316964	-0.234083206	-0.0105	0	0.380	-0.402
					3.447158	3.505149978	3.476154	0.057991947	-0.0105	0	0.380	-0.402
				Category 5 0,65 < aw ≤ 0,75	5.1172713	5.155336037	5.13630367	0.038064742	-0.0105	0	0.380	-0.402
					4.8808136	4.84509804	4.86295582	-0.035715552	-0.0105	0	0.380	-0.402
					4.39794	4.491361694	4.44465085	0.093421685	-0.0105	0	0.380	-0.402
					4.30103	4.204119983	4.25257499	-0.096910013	-0.0105	0	0.380	-0.402
					4.3159703	4.298853076	4.30741171	-0.017117269	-0.0105	0	0.380	-0.402
					4.845098	5.152288344	4.99869319	0.307190304	-0.0105	0	0.380	-0.402
					4.748188	4.812913357	4.78055069	0.06472533	-0.0105	0	0.380	-0.402
					4.5797836	4.531478917	4.55563126	-0.04830468	-0.0105	0	0.380	-0.402
					4.5185139	4.491361694	4.50493782	-0.027152246	-0.0105	0	0.380	-0.402
					4.2552725	4.041392685	4.1483326	-0.21387982	-0.0105	0	0.380	-0.402
					5.0899051	5.161368002	5.12563656	0.071462891	-0.0105	0	0.380	-0.402
					4.9444827	4.049218023	4.49685035	-0.895264649	-0.0105	0	0.380	-0.402
					4.39794	4.041392685	4.21966635	-0.356547324	-0.0105	0	0.380	-0.402
					3.9822712	3.897627091	3.93994916	-0.084644142	-0.0105	0	0.380	-0.402
					3.9294189	3.69019608	3.8098075	-0.239222846	-0.0105	0	0.380	-0.402
				Category 6 0,60 < aw ≤ 0,65	6.4955443	6.25791845	6.37673139	-0.237625887	-0.0105	0	0.380	-0.402
					6.3424227	6.167317335	6.25487001	-0.175105346	-0.0105	0	0.380	-0.402
					6.0170333	5.792391689	5.90471251	-0.22464165	-0.0105	0	0.380	-0.402
					5.6580114	5.617000341	5.63750587	-0.041011056	-0.0105	0	0.380	-0.402
					5.4393327	5.392696953	5.41601482	-0.046635741	-0.0105	0	0.380	-0.402
					2.9542425	2.84509804	2.89967027	-0.109144469	-0.0105	0	0.380	-0.402
					3.1760913	3.146128036	3.16110965	-0.029963223	-0.0105	0	0.380	-0.402
					2.9542425	3.301029996	3.12763625	0.346787486	-0.0105	0	0.380	-0.402
					3.146128	2.698970004	2.92254902	-0.447158031	-0.0105	0	0.380	-0.402
					3.0791812	2.84509804	2.96213964	-0.234083206	-0.0105	0	0.380	-0.402
					6.6901961	6.505149978	6.59767303	-0.185046102	-0.0105	0	0.380	-0.402
					6.2174839	5.995635195	6.10655957	-0.22184875	-0.0105	0	0.380	-0.402
					6.1335389	6.184691431	6.15911517	0.051152522	-0.0105	0	0.380	-0.402
					5.4683473	5.431363764	5.44985555	-0.036983566	-0.0105	0	0.380	-0.402
					5.0043214	4.819543936	4.91193265	-0.184777438	-0.0105	0	0.380	-0.402
				Category 7 0,60 < aw ≤ 0,65	3.146128	3.041392685	3.09376036	-0.104735351	-0.0105	0	0.380	-0.402
					3.3617278	3.230448921	3.29608838	-0.131278915	-0.0105	0	0.380	-0.402
					3.2787536	3.230448921	3.25460126	-0.04830468	-0.0105	0	0.380	-0.402
					3.3424227	3.176091259	3.25925697	-0.166331422	-0.0105	0	0.380	-0.402
					3.20412	3.079181246	3.14165061	-0.124938737	-0.0105	0	0.380	-0.402
					3.7323938	3.707570176	3.71998197	-0.024823584	-0.0105	0	0.380	-0.402
					2.69897	2.602059991	2.650515	-0.096910013	-0.0105	0	0.380	-0.402
					7.0374265	7.004321374	7.02087394	-0.033105124	-0.0105	0	0.380	-0.402
					6.0606978	6.120573931	6.09063589	0.059876091	-0.0105	0	0.380	-0.402
					6.2329961	5.591064607	5.91203036	-0.641931503	-0.0105	0	0.380	-0.402
					5.447158	5.372912003	5.41003502	-0.074246028	-0.0105	0	0.380	-0.402
					4.8633229	4.857332496	4.86032768	-0.005990364	-0.0105	0	0.380	-0.402
					4.3802112	4.568201724	4.47420648	0.187990482	-0.0105	0	0.380	-0.402
					4.39794	4.361727836	4.37983392	-0.036212173	-0.0105	0	0.380	-0.402
					4.2479733	4.278753601	4.26336343	0.030780335	-0.0105	0	0.380	-0.402
				Aver. Dall				-0.010501696				
				SDall				0.196243841				

Table S8. Raw data and statistical analysis of the agreement between the reference DG18 medium and the alternative **DSBM-III** medium variation for the enumeration of yeasts and moulds across selected food and feed categories and types.

According to **ISO 16140-2:2014**, the Probability for the Tolerance Interval (**Proba TI**) must be **80%** and the central value must be the **median.**

**Proba TI (beta):**	80%
**Central Value:**	Median

(Food) Category	1
(Food) Type	2

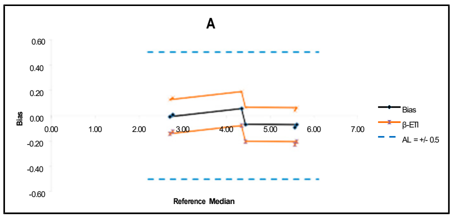

Sample Name	Reference central value	Bias	Lower β-ETI	Upper β-ETI	β-ETI compared to AL=±0.5 Acceptable	β-ETI compared to final AL Acceptable
	2.77	0.007	-0.127	0.142	YES	YES
	2.71	-0.009	-0.143	0.126	YES	YES
	4.34	0.056	-0.079	0.190	YES	YES
	4.43	-0.070	-0.204	0.065	YES	YES
	5.60	-0.071	-0.205	0.064	YES	YES
	5.56	-0.094	-0.228	0.040	YES	YES

	Reference method	Alternative method	SD repeatability of reference method <= 0.125	Final AL
SD Repeatability	0.093	0.093	YES	+/- 0.500

(Food) Category	2
(Food) Type	1

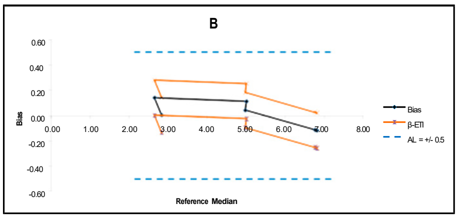

Sample Name	Reference Central value	Bias	Lower β-ETI	Upper β-ETI	β-ETI compared to AL=±0.5 Acceptable	β-ETI compared to final AL Acceptable
	2.83	0.006	-0.132	0.145	YES	YES
	2.64	0.142	0.003	0.280	YES	YES
	5.00	0.114	-0.025	0.253	YES	YES
	4.97	0.044	-0.095	0.182	YES	YES
	6.81	-0.116	-0.255	0.023	YES	YES
	6.76	-0.110	-0.249	0.028	YES	YES

	Reference method	Alternative method	SD repeatability of reference method <= 0.125	Final AL
SD Repeatability	0.093	0.096	YES	+/- 0.500

(Food) Category	3
(Food) Type	2

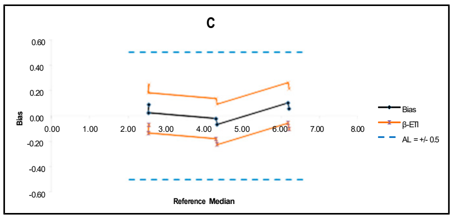

Sample Name	Reference Central value	Bias	Lower β-ETI	Upper β-ETI	β-ETI compared to AL=±0.5 Acceptable	β-ETI compared to final AL Acceptable
	2.54	0.089	-0.069	0.248	YES	YES
	2.53	0.024	-0.135	0.182	YES	YES
	4.30	-0.022	-0.181	0.137	YES	YES
	4.32	-0.067	-0.226	0.092	YES	YES
	6.18	0.103	-0.056	0.261	YES	YES
	6.20	0.056	-0.103	0.215	YES	YES

	Reference method	Alternative method	SD repeatability of reference method <= 0.125	Final AL
SD Repeatability	0.121	0.110	YES	+/- 0.500

(Food) Category	4
(Food) Type	2

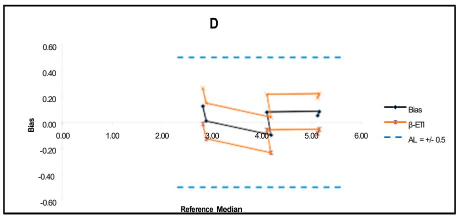

Sample Name	Reference Central value	Bias	Lower β-ETI	Upper β-ETI	β-ETI compared to AL=±0.5 Acceptable	β-ETI compared to final AL Acceptable
	2.81	0.127	-0.011	0.264	YES	YES
	2.88	0.011	-0.126	0.148	YES	YES
	4.18	-0.097	-0.234	0.040	YES	YES
	4.10	0.079	-0.058	0.216	YES	YES
	5.15	0.084	-0.053	0.221	YES	YES
	5.11	0.053	-0.084	0.191	YES	YES

	Reference method	Alternative method	SD repeatability of reference method <= 0.125	Final AL
SD Repeatability	0.088	0.095	YES	+/- 0.500

(Food) Category	5
(Food) Type	3

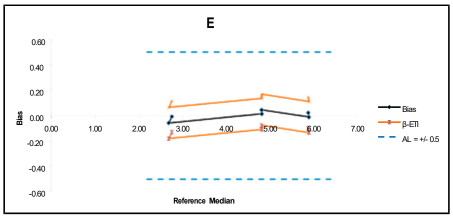

Sample Name	Reference Central value	Bias	Lower β-ETI	Upper β-ETI	β-ETI compared to AL=±0.5 Acceptable	β-ETI compared to final AL Acceptable
	2.75	-0.008	-0.131	0.115	YES	YES
	2.68	-0.058	-0.181	0.065	YES	YES
	4.81	0.013	-0.109	0.136	YES	YES
	4.80	0.046	-0.077	0.168	YES	YES
	5.88	-0.012	-0.134	0.111	YES	YES
	5.86	0.023	-0.100	0.146	YES	YES

	Reference method	Alternative method	SD repeatability of reference method <= 0.125	Final AL
SD Repeatability	0.093	0.085	YES	+/- 0.500

(Food) Category	6
(Food) Type	1

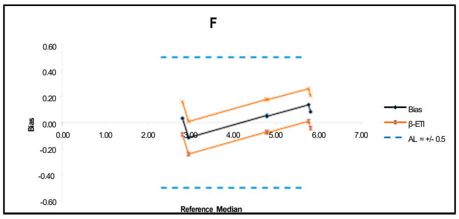

Sample Name	Reference Central value	Bias	Lower β-ETI	Upper β-ETI	β-ETI compared to AL=±0.5 Acceptable	β-ETI compared to final AL Acceptable
	2.81	0.033	-0.093	0.158	YES	YES
	2.95	-0.117	-0.242	0.009	YES	YES
	4.78	0.054	-0.071	0.180	YES	YES
	4.78	0.048	-0.078	0.174	YES	YES
	5.76	0.136	0.011	0.262	YES	YES
	5.80	0.081	-0.044	0.207	YES	YES

	Reference method	Alternative method	SD repeatability of reference method <= 0.125	Final AL
SD Repeatability	0.069	0.087	YES	+/- 0.500

(Feed) Category	7
(Feed) Type	2

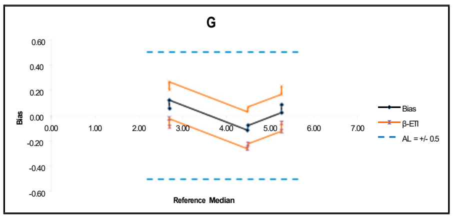

Sample Name	Reference Central value	Bias	Lower β-ETI	Upper β-ETI	β-ETI compared to AL=±0.5 Acceptable	β-ETI compared to final AL Acceptable
	2.70	0.057	-0.089	0.203	YES	YES
	2.69	0.123	-0.023	0.269	YES	YES
	4.48	-0.115	-0.261	0.030	YES	YES
	4.49	-0.076	-0.222	0.069	YES	YES
	5.26	0.023	-0.122	0.169	YES	YES
	5.26	0.087	-0.059	0.233	YES	YES

	Reference method	Alternative method	SD repeatability of reference method <= 0.125	Final AL
SD Repeatability	0.102	0.101	YES	+/- 0.500

Figure S7. Bias and β-ETI limits, compliance with predefined acceptability criteria, and graphical presentation of the accuracy profile (AP) study results for **yeast** enumeration using the **DSBM-I** medium variation. The panels in Figure S7 show the results for the following food and feed categories and types: (A) Category 1, Type 2, (B) Category 2, Type 1, (C) Category 3, Type 2, (D) Category 4, Type 2, (E) Category 5, Type 3, (F) Category 6, Type 1, (G) Category 7, Type 2. Bias is expressed as $\log_{10}$ CFU/g. Black lines indicate bias, orange lines indicate the 80% β-expectation tolerance intervals (β-ETI), and blue dashed lines indicate the acceptability limits (AL) of ±0.5 $\log_{10}$ CFU/g. Two samples were analyzed at each contamination level (low, intermediate, and high), with five replicate determinations performed per sample. The categories and selected product types included in the study are listed in Table 1 of the manuscript.

According to **ISO 16140-2:2014**, the Probability for the Tolerance Interval (**Proba TI**) must be **80%** and the central value must be the **median.**

**Proba TI (beta):**	80%
**Central Value:**	Median

(Food) Category	1
(Food) Type	2

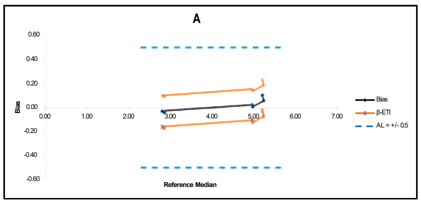

Sample Name	Reference central value	Bias	Lower β-ETI	Upper β-ETI	β-ETI compared to AL=±0.5 Acceptable	β-ETI compared to final AL Acceptable
	2.85	-0.039	-0.169	0.091	YES	YES
	2.79	-0.029	-0.159	0.101	YES	YES
	4.95	0.023	-0.106	0.153	YES	YES
	4.96	0.009	-0.121	0.139	YES	YES
	5.23	0.058	-0.072	0.188	YES	YES
	5.19	0.104	-0.026	0.233	YES	YES

	Reference method	Alternative method	SD repeatability of reference method <= 0.125	Final AL
SD Repeatability	0.080	0.090	YES	+/- 0.500

(Food) Category	2
(Food) Type	1

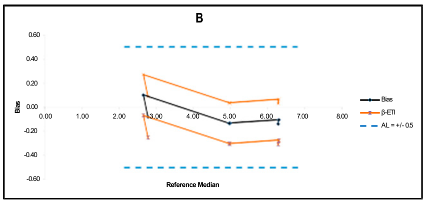

Sample Name	Reference Central value	Bias	Lower β-ETI	Upper β-ETI	β-ETI compared to AL=±0.5 Acceptable	β-ETI compared to final AL Acceptable
	2.78	-0.079	-0.248	0.090	YES	YES
	2.65	0.103	-0.066	0.272	YES	YES
	4.98	-0.133	-0.302	0.036	YES	YES
	4.96	-0.131	-0.300	0.038	YES	YES
	6.30	-0.104	-0.272	0.065	YES	YES
	6.28	-0.137	-0.306	0.032	YES	YES

	Reference method	Alternative method	SD repeatability of reference method <= 0.125	Final AL
SD Repeatability	0.109	0.117	YES	+/- 0.500

(Food) Category	3
(Food) Type	2

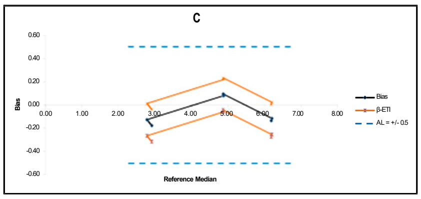

Sample Name	Reference Central value	Bias	Lower β-ETI	Upper β-ETI	β-ETI compared to AL=±0.5 Acceptable	β-ETI compared to final AL Acceptable
	2.91	-0.176	-0.315	-0.038	YES	YES
	2.78	-0.125	-0.264	0.014	YES	YES
	4.88	0.079	-0.059	0.218	YES	YES
	4.87	0.095	-0.044	0.233	YES	YES
	6.19	-0.114	-0.253	0.025	YES	YES
	6.18	-0.135	-0.273	0.004	YES	YES

	Reference method	Alternative method	SD repeatability of reference method <= 0.125	Final AL
SD Repeatability	0.100	0.096	YES	+/- 0.500

(Food) Category	4
(Food) Type	2

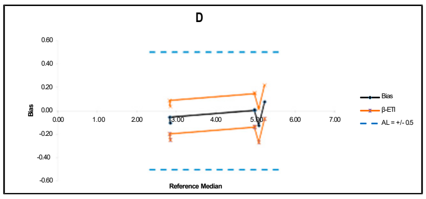

Sample Name	Reference Central value	Bias	Lower β-ETI	Upper β-ETI	β-ETI compared to AL=±0.5 Acceptable	β-ETI compared to final AL Acceptable
	2.85	-0.105	-0.248	0.038	YES	YES
	2.83	-0.054	-0.197	0.089	YES	YES
	4.99	0.004	-0.138	0.147	YES	YES
	4.96	0.009	-0.134	0.152	YES	YES
	5.08	-0.125	-0.268	0.018	YES	YES
	5.23	0.076	-0.067	0.219	YES	YES

	Reference method	Alternative method	SD repeatability of reference method <= 0.125	Final AL
SD Repeatability	0.101	0.099	YES	+/- 0.500

(Food) Category	5
(Food) Type	3

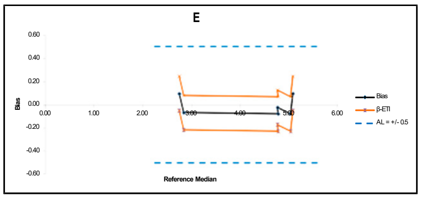

Sample Name	Reference Central value	Bias	Lower β-ETI	Upper β-ETI	β-ETI compared to AL=±0.5 Acceptable	β-ETI compared to final AL Acceptable
	2.75	0.097	-0.052	0.246	YES	YES
	2.84	-0.068	-0.217	0.081	YES	YES
	4.78	-0.079	-0.228	0.070	YES	YES
	4.76	-0.023	-0.172	0.126	YES	YES
	5.03	-0.079	-0.228	0.070	YES	YES
	5.08	0.097	-0.052	0.246	YES	YES

	Reference method	Alternative method	SD repeatability of reference method <= 0.125	Final AL
SD Repeatability	0.100	0.103	YES	+/- 0.500

(Food) Category	6
(Food) Type	1

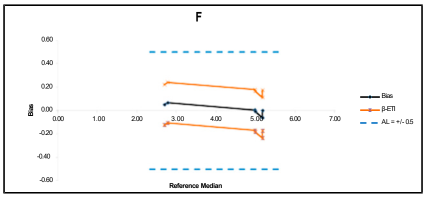

Sample Name	Reference Central value	Bias	Lower β-ETI	Upper β-ETI	β-ETI compared to AL=±0.5 Acceptable	β-ETI compared to final AL Acceptable
	2.70	0.049	-0.124	0.222	YES	YES
	2.78	0.067	-0.106	0.240	YES	YES
	4.98	0.005	-0.169	0.178	YES	YES
	4.99	-0.009	-0.182	0.164	YES	YES
	5.18	-0.062	-0.235	0.111	YES	YES
	5.18	0.000	-0.173	0.173	YES	YES

	Reference method	Alternative method	SD repeatability of reference method <= 0.125	Final AL
SD Repeatability	0.110	0.120	YES	+/- 0.500

(Feed) Category	7
(Feed) Type	2

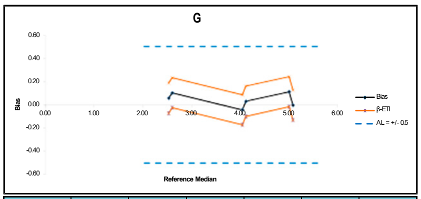

Sample Name	Reference Central value	Bias	Lower β-ETI	Upper β-ETI	β-ETI compared to AL=±0.5 Acceptable	β-ETI compared to final AL Acceptable
	2.53	0.060	-0.070	0.190	YES	YES
	2.60	0.106	-0.024	0.235	YES	YES
	4.04	-0.041	-0.171	0.089	YES	YES
	4.11	0.032	-0.098	0.162	YES	YES
	5.00	0.114	-0.016	0.244	YES	YES
	5.08	0.000	-0.130	0.130	YES	YES

	Reference method	Alternative method	SD repeatability of reference method <= 0.125	Final AL
SD Repeatability	0.109	0.090	YES	+/- 0.500

Figure S8. Bias and β-ETI limits, compliance with predefined acceptability criteria, and graphical presentation of the accuracy profile (AP) study results for **yeast** enumeration using the **DSBM-II** medium variation. The panels in Figure S8 show the results for the following food and feed categories and types: (A) Category 1, Type 2, (B) Category 2, Type 1, (C) Category 3, Type 2, (D) Category 4, Type 2, (E) Category 5, Type 3, (F) Category 6, Type 1, (G) Category 7, Type 2. Bias is expressed as log_{10} CFU/g. Black lines indicate bias, orange lines indicate the 80% β-expectation tolerance intervals (β-ETI), and blue dashed lines indicate the acceptability limits (AL) of ±0.5 log_{10} CFU/g. Two samples were analyzed at each contamination level (low, intermediate, and high), with five replicate determinations performed per sample. The categories and selected product types included in the study are listed in Table 1 of the manuscript.

According to **ISO 16140-2:2014**, the Probability for the Tolerance Interval (**Proba TI**) must be **80%** and the central value must be the **median.**

**Proba TI (beta):**	80%
**Central Value:**	Median

Global Results

(Food) Category	1
(Food) Type	2

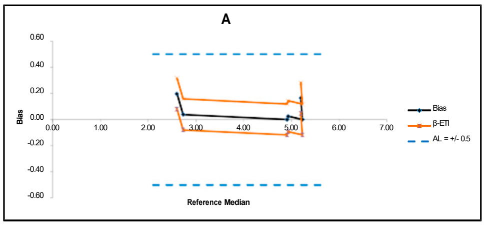

Sample Name	Reference central value	Bias	Lower β-ETI	Upper β-ETI	β-ETI compared to AL=±0.5 Acceptable	β-ETI compared to final AL Acceptable
	2.60	0.197	0.079	0.316	YES	YES
	2.73	0.038	-0.080	0.157	YES	YES
	4.90	0.000	-0.118	0.118	YES	YES
	4.93	0.025	-0.094	0.143	YES	YES
	5.23	0.000	-0.118	0.118	YES	YES
	5.19	0.165	0.046	0.283	YES	YES

	Reference method	Alternative method	SD repeatability of reference method <= 0.125	Final AL
SD Repeatability	0.097	0.082	YES	+/- 0.500

(Food) Category	2
(Food) Type	1

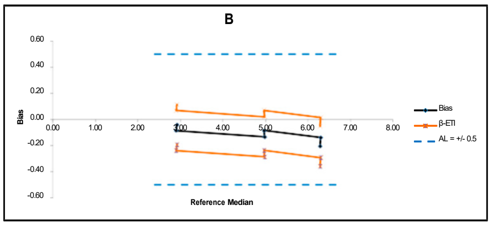

Sample Name	Reference Central value	Bias	Lower β-ETI	Upper β-ETI	β-ETI compared to AL=±0.5 Acceptable	β-ETI compared to final AL Acceptable
	2.91	-0.039	-0.192	0.114	YES	YES
	2.90	-0.085	-0.238	0.068	YES	YES
	4.98	-0.133	-0.286	0.020	YES	YES
	4.96	-0.083	-0.236	0.070	YES	YES
	6.30	-0.138	-0.291	0.015	YES	YES
	6.28	-0.204	-0.357	-0.051	YES	YES

	Reference method	Alternative method	SD repeatability of reference method <= 0.125	Final AL
SD Repeatability	0.097	0.106	YES	+/- 0.500

(Food) Category	3
(Food) Type	2

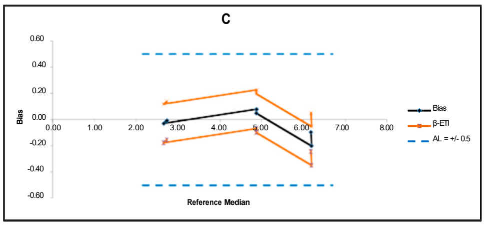

Sample Name	Reference Central value	Bias	Lower β-ETI	Upper β-ETI	β-ETI compared to AL=±0.5 Acceptable	β-ETI compared to final AL Acceptable
	2.73	-0.008	-0.155	0.139	YES	YES
	2.65	-0.030	-0.177	0.117	YES	YES
	4.88	0.079	-0.068	0.226	YES	YES
	4.87	0.050	-0.097	0.197	YES	YES
	6.19	-0.202	-0.349	-0.055	YES	YES
	6.18	-0.097	-0.244	0.050	YES	YES

	Reference method	Alternative method	SD repeatability of reference method <= 0.125	Final AL
SD Repeatability	0.101	0.102	YES	+/- 0.500

(Food) Category	4
(Food) Type	2

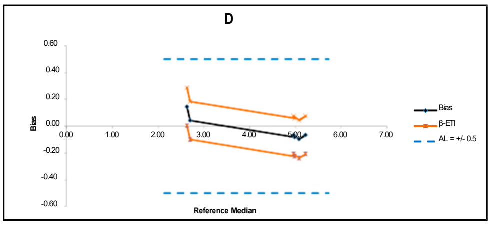

Sample Name	Reference Central value	Bias	Lower β-ETI	Upper β-ETI	β-ETI compared to AL=±0.5 Acceptable	β-ETI compared to final AL Acceptable
	2.63	0.145	0.003	0.286	YES	YES
	2.70	0.041	-0.100	0.183	YES	YES
	4.99	-0.084	-0.225	0.058	YES	YES
	4.96	-0.066	-0.208	0.075	YES	YES
	5.08	-0.097	-0.238	0.045	YES	YES
	5.23	-0.067	-0.208	0.075	YES	YES

	Reference method	Alternative method	SD repeatability of reference method <= 0.125	Final AL
SD Repeatability	0.117	0.098	YES	+/- 0.500

(Food) Category	5
(Food) Type	3

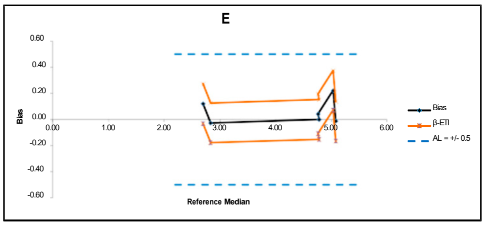

Sample Name	Reference Central value	Bias	Lower β-ETI	Upper β-ETI	β-ETI compared to AL=±0.5 Acceptable	β-ETI compared to final AL Acceptable
	2.70	0.121	-0.031	0.272	YES	YES
	2.83	-0.026	-0.178	0.125	YES	YES
	4.78	0.000	-0.152	0.152	YES	YES
	4.76	0.043	-0.109	0.194	YES	YES
	5.03	0.222	0.070	0.373	YES	YES
	5.08	-0.013	-0.165	0.138	YES	YES

	Reference method	Alternative method	SD repeatability of reference method <= 0.125	Final AL
SD Repeatability	0.103	0.105	YES	+/- 0.500

(Food) Category	6
(Food) Type	1

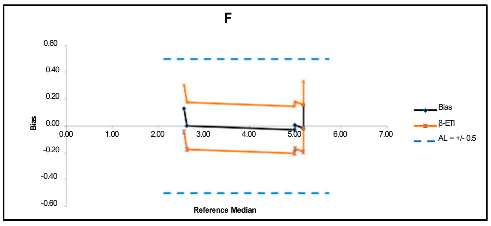

Sample Name	Reference Central value	Bias	Lower β-ETI	Upper β-ETI	β-ETI compared to AL=±0.5 Acceptable	β-ETI compared to final AL Acceptable
	2.57	0.131	-0.044	0.305	YES	YES
	2.63	0.000	-0.175	0.175	YES	YES
	4.98	-0.028	-0.203	0.147	YES	YES
	4.99	0.009	-0.166	0.183	YES	YES
	5.18	-0.018	-0.192	0.157	YES	YES
	5.18	0.158	-0.016	0.333	YES	YES

	Reference method	Alternative method	SD repeatability of reference method <= 0.125	Final AL
SD Repeatability	0.121	0.121	YES	+/- 0.500

(Feed) Category	7
(Feed) Type	2

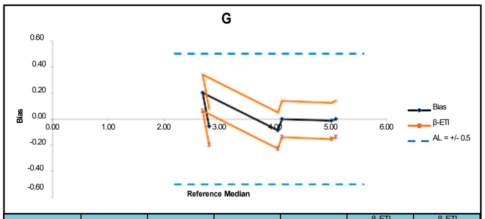

Sample Name	Reference Central value	Bias	Lower β-ETI	Upper β-ETI	β-ETI compared to AL=±0.5 Acceptable	β-ETI compared to final AL Acceptable
	2.81	-0.058	-0.197	0.081	YES	YES
	2.69	0.202	0.063	0.340	YES	YES
	4.04	-0.087	-0.226	0.051	YES	YES
	4.11	0.000	-0.139	0.139	YES	YES
	5.00	-0.013	-0.152	0.125	YES	YES
	5.08	0.000	-0.139	0.139	YES	YES

	Reference method	Alternative method	SD repeatability of reference method <= 0.125	Final AL
SD Repeatability	0.089	0.096	YES	+/- 0.500

Figure S9. Bias and β-ETI limits, compliance with predefined acceptability criteria, and graphical presentation of the accuracy profile (AP) study results for **yeast** enumeration using the **DSBM-III** medium variation. The panels in Figure S8 show the results for the following food and feed categories and types: (A) Category 1, Type 2, (B) Category 2, Type 1, (C) Category 3, Type 2, (D) Category 4, Type 2, (E) Category 5, Type 3, (F) Category 6, Type 1, (G) Category 7, Type 2. Bias is expressed as log_{10} CFU/g. Black lines indicate bias, orange lines indicate the 80% β-expectation tolerance intervals (β-ETI), and blue dashed lines indicate the acceptability limits (AL) of ±0.5 log_{10} CFU/g. Two samples were analyzed at each contamination level (low, intermediate, and high), with five replicate determinations performed per sample. The categories and selected product types included in the study are listed in Table 1 of the manuscript.

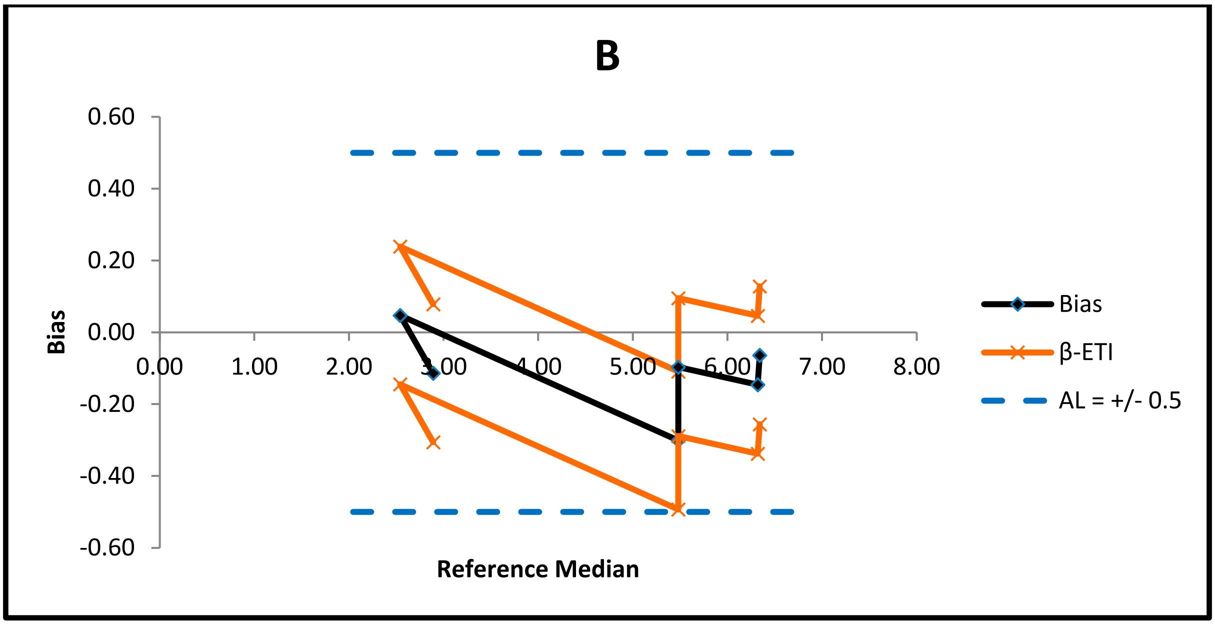

Figure S4 panel B
